# First-in-class ultralong-target-residence-time p38α inhibitors as a mitosis-targeted therapy for colorectal cancer

**DOI:** 10.1038/s43018-024-00899-7

**Published:** 2025-01-16

**Authors:** Ramona Rudalska, Jule Harbig, Michael Forster, Pascal Woelffing, Aylin Esposito, Mark Kudolo, Adelina Botezatu, Vanessa Haller, Nicole Janssen, Samuel Holzmayer, Philipp Nahidino, Omelyan Trompak, Tatu Pantsar, Thales Kronenberger, Can Yurttas, Elke Rist, Alexander N. R. Weber, Marc H. Dahlke, German Ott, Alfred Koenigsrainer, Ulrich Rothbauer, Melanie Maerklin, Thomas Muerdter, Matthias Schwab, Stephan Singer, Lars Zender, Stefan Laufer, Daniel Dauch

**Affiliations:** 1 Department of Medical Oncology and Pneumology, University Hospital Tübingen, Tübingen, Germany; 2 IFIT Cluster of Excellence EXC 2180 ‘Image-Guided and Functionally Instructed Tumor Therapies’, University of Tübingen, Tübingen, Germany; 3 Department of Pharmaceutical Chemistry, University of Tübingen, Tübingen, Germany; 4 Dr. Margarete Fischer-Bosch Institute of Clinical Pharmacology, Stuttgart and University of Tübingen, Tübingen, Germany; 5 Clinical Collaboration Unit Translational Immunology, German Cancer Consortium (DKTK), Department of Internal Medicine, University Hospital Tübingen, Tübingen, Germany; 6 School of Pharmacy, Faculty of Health Sciences, University of Eastern Finland, Kuopio, Finland; 7 Department of General, Visceral and Transplant Surgery, University Hospital Tübingen, Tübingen, Germany; 8 Department of Immunology, University of Tübingen, Tübingen, Germany; 9 Department of General and Visceral Surgery, Robert Bosch Hospital, Stuttgart, Germany; 10 Department of Clinical Pathology, Robert Bosch Hospital, Stuttgart, Germany; 11 NMI Natural and Medical Sciences Institute at the University of Tübingen, Reutlingen, Germany; 12 Department of Pharmaceutical Biotechnology, University of Tübingen, Tübingen, Germany; 13 Departments of Clinical Pharmacology, and of Biochemistry and Pharmacy, University of Tübingen, Tübingen, Germany; 14 German Cancer Research Consortium (DKTK), Partner Site Tübingen, German Cancer Research Center (DKFZ), Heidelberg, Germany; 15 Institute of Pathology and Neuropathology, University Hospital Tübingen, Tübingen, Germany; 16 Tübingen Center for Academic Drug Discovery and Development (TüCAD2), Tübingen, Germany

**Keywords:** Colorectal cancer, Targeted therapies, Cancer therapeutic resistance, Drug development, Cancer

## Abstract

Colorectal cancer (CRC) constitutes the second leading cause of cancer-related death worldwide and advanced CRCs are resistant to targeted therapies, chemotherapies and immunotherapies. p38α (*Mapk14*) has been suggested as a therapeutic target in CRC; however, available p38α inhibitors only allow for insufficient target inhibition. Here we describe a unique class of p38α inhibitors with ultralong target residence times (designated ULTR-p38i) that robustly inhibit p38α downstream signaling and induce distinct biological phenotypes. ULTR-p38i monotherapy triggers an uncontrolled mitotic entry by activating Cdc25 and simultaneously blocking Wee1. Consequently, CRC cells undergo mitotic catastrophe, resulting in apoptosis or senescence. ULTR-p38i exhibit high selectivity, good pharmaco-kinetic properties and no measurable toxicity with strong therapeutic effects in patient-derived CRC organoids and syngeneic CRC mouse models. Conceptually, our study suggests ultralong-target-residence-time kinase inhibitors as an alternative to covalent inhibitors, which, because of the lack of cysteine residues, cannot be generated for many kinase cancer targets.

## Abstract (web summary)

Rudalska et al. describe a novel class of p38α inhibitors with increased target residence time. They explore the drugs’ specificity, pharmacokinetics and toxicity profile and show that they are efficacious in the context of colorectal cancer.

## Main

The ability of solid tumors to develop resistance to targeted cancer therapies remains a major obstacle to the efficient treatment of persons with cancer. Despite major advances in target identification and an increasing number of clinical trials, the therapeutic success of targeted therapies in solid tumor entities remains very limited^1,2^.

With more than 1.8 million new cases and around 900,000 deaths annually, colorectal cancer (CRC) constitutes the third most commonly diagnosed malignancy and the second leading cause of cancer-related death worldwide^3^. While endoscopic excision and surgical resection of localized tumors and polyps represents an efficient treatment, the therapeutic options for persons with advanced CRC remain very limited^4,5^. CRCs show strong resistance to targeted therapies and even personalized treatment approaches show poor results^5,6^. In addition, immunotherapies are mostly ineffective in CRC^7,8^. Only microsatellite-instable, mismatch-repair-deficient CRCs, which account for only ~15% of all CRCs^9^, may respond to checkpoint-blocking antibodies such as anti-programmed cell death protein 1 or anti-cytotoxic T lymphocyte-associated protein 4 (refs. ^7,10^). Therefore, treatment of persons with advanced CRC still relies on oxaliplatin- and irinotecan-based chemotherapies in combination with monoclonal antibodies directed against vascular endothelial growth factor receptor (VEGFR) or epidermal growth factor receptor (EGFR). However, these combinatorial therapies only moderately improve the survival of persons with metastatic CRC (median overall survival between 24 and 27 months)^5^. It, thus, becomes clear that better therapeutic options for persons with CRC are urgently needed.

p38α belongs to the mitogen-activated protein kinase (MAPK) family and can be activated by environmental stress and proinflammatory cytokines^11,12^. Because of its role in inflammation, autophagy, oncogenic signaling pathways and the tumor microenvironment, a potential role of p38α in CRC development was suggested^13^ and a genetic loss of the p38α gene *Mapk14* markedly reduced tumor development in a mouse model of colitis-associated CRC^14^.

Strategies to pharmacologically inhibit p38α were initially pursued to block inflammation in chronic inflammatory diseases^15,16^. While first-generation type I or type II p38α inhibitors failed in clinics because of either structure-based liver toxicity or adverse effects caused by poor kinome selectivity, second-generation type I inhibitors, such as skepinone-L (SKL), PH-797804 or LY2228820 (ralimetinib), showed no structure-based toxicity, good pharmacokinetic properties and a better selectivity profile^17–19^. These compounds were also suggested for the treatment of different tumor entities, often as components of combinatorial therapies. LY2228820 has been tested in different phase 1 or 2 clinical trials against ovarian cancer, glioblastoma and metastatic breast cancer (NCT02322853, NCT02364206, NCT01663857, NCT01393990 and NCT02860780)^20,21^. Moreover, we found that SKL and PH-797804 increased the therapeutic efficacy of the multikinase inhibitor sorafenib in liver cancer^22^. However, to date, no p38α inhibitor has been approved for the treatment of persons with cancer.

Here, we report the development and characterization of a unique class of p38α inhibitors (type 1.5 binders) with ultralong target residence times (TRTs; very low *k*_off_ values), designated ULTR-p38i. These compounds, in strong contrast to first-generation and second-generation p38α inhibitors, faithfully resemble phenotypes obtained after genetic knockdown of *Mapk14* and show marked therapeutic efficacy in CRC mouse models and patient-derived CRC organoids as a monotherapy. Mechanistic studies revealed that a sustained inhibition of p38α simultaneously blocks Wee1 and induces Cdc25 activity, resulting in an uncontrolled mitotic entry, mitotic catastrophe, aneuploidy or polyploidy and apoptosis or senescence of CRC cells. Conceptually, ultralong-TRT kinase inhibitors represent a promising alternative to covalent inhibitors, which, because of the lack of cysteine residues, cannot be generated for many kinase cancer targets.

## Results

### Type 1.5 p38α inhibitors show therapeutic efficacy in CRC

**Fig. 1:**
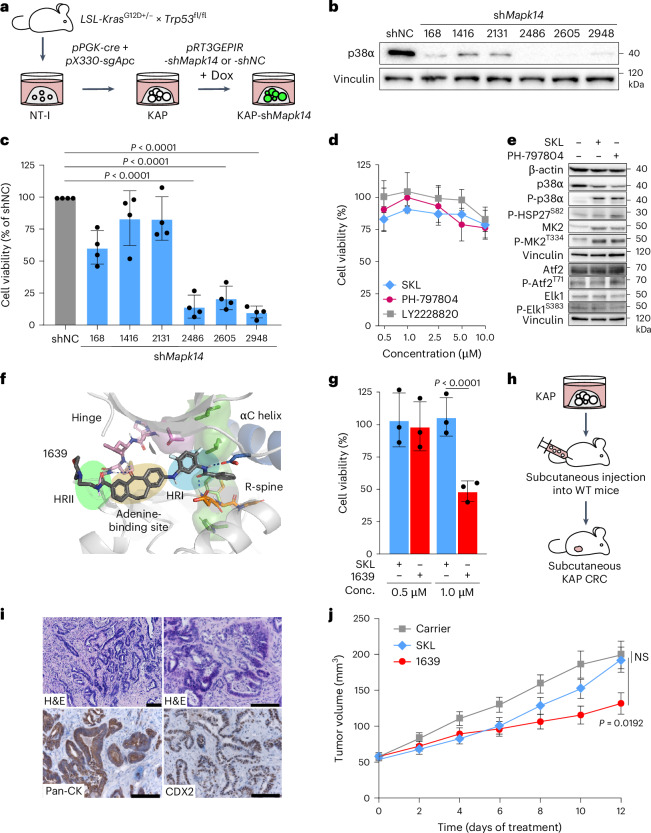
CRC organoids are resistant to type I but sensitive to type 1.5 p38α inhibitors. **a**, Generation of KAP organoids expressing *Mapk14* shRNAs. **b**, Knockdown test of *Mapk14* shRNAs. Representative western blot analysis of p38α in KAP^2D^ cells upon 6 days of treatment with doxycycline (Dox; cropped blot images, *n* = 3 biologically independent experiments). **c**, Cell viability analysis in KAP-sh*Mapk14* and KAP-shNC organoids upon 12 days of treatment with doxycycline (*n* = 4 biologically independent experiments; data are presented as the mean ± s.d.). Statistical significance was calculated using an ANOVA and Dunnett’s multiple-comparisons test (*P* < 0.0001). **d**, Cell viability analysis in KAP organoids upon 4 days of treatment with SKL, PH-797804, LY2228820 or DMSO (*n* = 3 biologically independent experiments; data are presented as the mean ± s.d.). **e**, Representative western blot analysis of KAP^2D^ cells upon 1 day of treatment with 5 µM SKL, PH-797804 or DMSO (cropped blot images; *n* = 3 biologically independent experiments). **f**, Schematic picture showing the binding of 1639 to HRI, HRII and R-spine of the p38α kinase. **g**, Cell viability analysis of KAP organoids upon 4 days of treatment with SKL, 1639 or DMSO (*n* = 3 biologically independent experiments; data are presented as the mean ± s.d.). Statistical significance was calculated using a two-tailed Student’s *t*-test (*P* < 0.0001). Conc., concentration. **h**, Generation of a CRC mouse model based on subcutaneous injection of KAP organoids into wild-type (WT) mice. **i**, Representative pictures of hematoxylin and eosin (H&E) and immunohistochemical staining for pan-CK and CDX2 in KAP subcutaneous tumors, 19 days after tumor initiation (*n* = 4 tumors per group). Scale bars, 100 µm. **j**, Treatment of subcutaneous KAP CRCs with SKL, 1639 or carrier (*n* = 10 tumors per group; data are presented as the mean ± s.e.m.). Statistical significance was calculated using an ANOVA and Dunnett’s multiple-comparisons test (*P* = 0.0192). NS, not significant. Treatment was started 1 week after organoid transplantation. The experiments in **b**, **d**, **e** and **g** were independently performed three times, the experiment in **c** was independently performed four times and the stainings in **i** were independently performed twice, all with similar results. Source data

**Extended Data Fig. 1:**
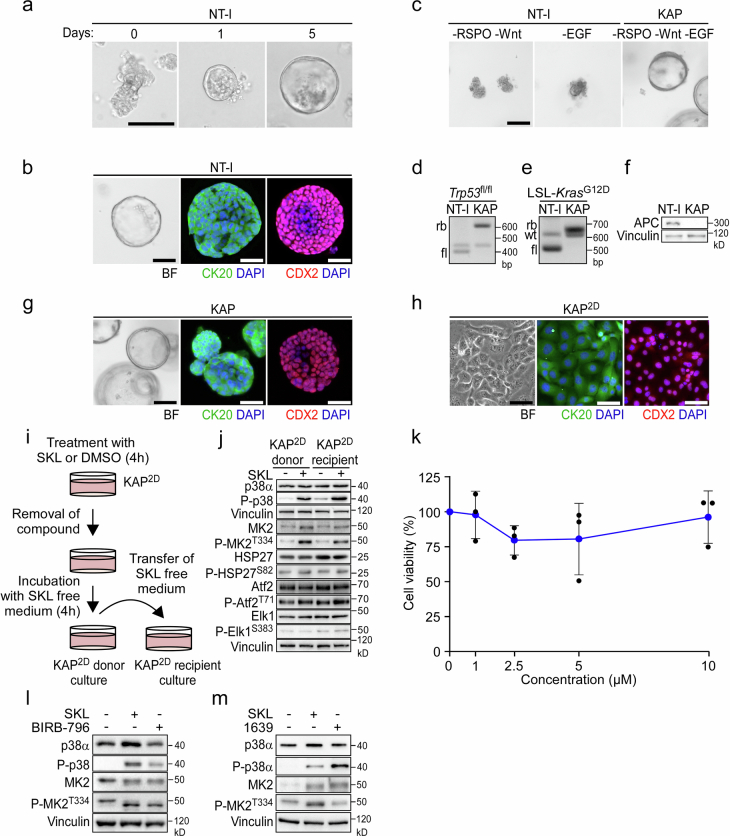
Generation and treatment of CRC cultures. **a**, Representative pictures of NT-I colonoids after 0, 1 and 5 d of isolation. Scale bar, 100 µm. **b**, Representative pictures of NT-I colonoids stained for CK20 and CDX2 (*n* = 2 biologically independent experiments). Scale bars, 100 µm. **c**, Representative pictures of NT-I and KAP organoids upon withdrawal of R-Spondin-1 (RSPO), Wnt and EGF. Scale bar, 100 µm. **d**, Representative PCR analysis of *cre* recombinase-mediated modifications of *Trp53* in KAP organoids (in comparison to NT-I, *n* = 2 biologically independent experiments). 370 bp = floxed (fl), 612 bp = recombined (rb) allele. **e**, Representative PCR analysis of *cre*-mediated modifications of LSL-*Kras*^G12D^ in KAP organoids (in comparison to NT-I, *n* = 2 biologically independent experiments). 500 bp = floxed (fl), 622 bp = WT and 650 bp = recombined (rb) allele. **f**, Representative western blot analysis of Apc in NT-I and KAP organoids (cropped blot images, *n* = 3 biologically independent experiments). **g**, Representative pictures of KAP organoids stained for CK20 and CDX2 (*n* = 2 biologically independent experiments). Scale bars, 50 µm. **h**, Representative pictures of KAP^2D^ cells stained for CK20 and CDX2 (*n* = 2 biologically independent experiments). Scale bars, 100 µm. **i**, Schematic outline of medium exchange experiment in KAP^2D^. **j**, Representative western blot analysis in KAP^2D^ donor- and KAP^2D^- recipient cells (cropped blot images, *n* = 3 biologically independent experiments). **k**, Cell viability analysis in KAP organoids upon 4 d of treatment with BIRB-796 or DMSO (*n* = 3 biologically independent experiments, data are presented as mean values +/- SD). **l**, Representative western blot analysis of p38α, P-p38α^T180/Y182^, MK2 and P-MK2^T334^ in KAP^2D^ upon 1 d of treatment with 5 µM SKL, BIRB-796 or DMSO (cropped blot images, *n* = 3 biologically independent experiments). **m**, Representative western blot analysis of p38α, P-p38α^T180/Y182^, MK2 and P-MK2^T334^ in KAP^2D^ cells upon 1 d of treatment with 1 µM SKL, 1639 or DMSO (cropped blot images, *n* = 3 biologically independent experiments). The experiments in Extended Data Fig. 1b, d, e, g, h were independently performed twice and the experiments in Extended Data Fig. 1f, j, k, l, m were independently performed three times, all with similar results. Source data

To study the role of p38α in colitis-independent CRC, we generated murine organotypic CRC cultures. We established colonoids from untransformed colon epithelial cells of LSL-*Kras*^G12D+/−^ × *Trp53*^fl/fl^ mice (NT-I) and transformed these into CRC in vitro by *cre*-mediated recombination of LSL-*Kras*^G12D^ and *Trp53*^fl/fl^ alleles and CRISPR–Cas9-mediated gene editing of *Apc* (Fig. 1a and Extended Data Fig. 1a–f). Resulting *Kras*^G12D^;*Apc*^+/−^;*Trp53*^−/−^ organoids (hereafter referred to as KAP) showed expression of established CRC markers (cytokeratin 20 (CK20) and homeobox protein CDX2; Extended Data Fig. 1g) and were also used to generate a CK20^+^CDX2^+^ two-dimensional CRC cell line (KAP^2D^; Extended Data Fig. 1h).

To determine the therapeutic potential of p38α inhibition in CRC, we conducted short hairpin RNA (shRNA)-mediated knockdown of *Mapk14* in CRC organoids. Six tetracycline-inducible shRNAs targeting different mRNA regions of *Mapk14* and a nontargeting control shRNA (shNC) were retrovirally delivered into KAP organoids and KAP^2D^ cells and shRNA expression was induced with doxycycline (Fig. 1a). Western blot analyses revealed a marked reduction in p38α protein levels by three shRNAs (2486, 2605 and 2948; Fig. 1b), whereas the other shRNAs only showed moderate *Mapk14* suppression. While a partial knockdown of *Mapk14* barely influenced the viability of CRC cells, pronounced induction of cell death in KAP organoids was found upon efficient *Mapk14* knockdown (Fig. 1c).

We next tested the outcome of pharmacological p38α inhibition in CRC. The established type I p38α inhibitors SKL, PH-797804 and LY2228820 (refs. ^17–19^) were used to conduct viability assays in KAP organoids. However, treatment with these inhibitors resulted in only a very weak induction of cell death, even when high concentrations were used (Fig. 1d).

To analyze whether type I p38α inhibitors are able to block p38α signaling in CRC, the activities of p38α and its downstream targets heat-shock protein 27 (Hsp27), MAPK-activated protein kinase 2 (MK2), activating transcription factor 2 (Atf2) and ETS transcription factor Elk1 (refs. ^16,23,24^) were determined by western blot analyses in KAP^2D^ cells. Interestingly, we found activation of p38α (P-p38α^T180/Y182^) and MK2 (P-MK2^T334^) upon treatment and no inhibition of Hsp27 (P-Hsp27^S82^), Atf2 (P-Atf2^T71^) and Elk1 (P-Elk1^S383^) could be observed (Fig. 1e). By means of a medium-exchange experiment, for which KAP^2D^ cells (KAP^2D^ donor cells) were treated either with SKL or DMSO, then incubated with SKL-free medium and finally transferred to untreated recipient cells (Extended Data Fig. 1i), we found that the inhibitor-induced p38α–MK2 hyperactivation was indirect and mediated by secreted factors (Extended Data Fig. 1j).

In contrast to type I inhibitors, type II kinase inhibitors bind only to inactive kinases and may allow for stabilizing the inactive kinase conformation^25^. However, the type II p38α inhibitor BIRB-796 (ref. ^26^) was likewise not capable of preventing p38α reactivation in CRC and also failed to inhibit p38α downstream signaling and induce cell death in KAP organoids (Extended Data Fig. 1k,l).

To allow for a more efficient inhibition of p38α, we recently developed p38α inhibitors that bind to hydrophobic regions I and II (HRI and HRII) and interact with the regulatory spine (R-spine) of the kinase^27,28^ (Fig. 1f). These agents show high specificities, increased TRTs, low adenosine triphosphate (ATP) competition and the ability to bind to the active and inactive conformation of the kinase^27,29^. Because these compounds show features of type I and II inhibitors, they were designated as type 1.5 p38α inhibitors. A prototypic 1.5 p38α compound (1639; Fig. 1f) showed a very low half-maximal inhibitory concentration (IC_50_) value (<3 nM, on isolated kinase), an increased TRT (184 s; *k*_off_ = 5.43 × 10^−3^ s^−1^) in contrast to SKL (TRT = 88 s; *k*_off_ = 1.14 × 10^−2^ s^−1^) and good pharmacokinetic properties upon oral administration in mice (*C*^max^ = 6.7 µM, *t*_1/2_ = 119.7 min). To determine whether 1639 harbors increased therapeutic efficacy in CRC, this compound was tested side by side with SKL in KAP organoids. Although 1639 still triggered p38α hyperactivation, we found reduced MK2^T334^ phosphorylation (Extended Data Fig. 1m) and a moderate but significant induction of cell death in 1639-treated KAP organoid cultures (Fig. 1g).

To compare the therapeutic efficacy of SKL and 1639 in vivo, we generated an organoid-based syngeneic CRC mouse model. Dissociated KAP organoids were subcutaneously injected into C57BL/6 wild-type mice, resulting in a rapid formation of macroscopically visible tumors within 1 week (Fig. 1h). Immunohistochemical characterization of tumor samples revealed a histological phenotype resembling well to moderately differentiated human CRC with a pronounced stroma compartment (Fig. 1i). Upon tumor formation (7 days after cell injection), mice were treated daily with vehicle, SKL or 1639. While 1639 decelerated CRC development in contrast to SKL (Fig. 1j), this compound was not able to fully block CRC tumor expansion over time (Fig. 1j). Overall, these data indicate that type 1.5 p38α kinase inhibitors with increased TRTs may harbor potential for CRC treatment; however, further optimized compounds are needed to increase therapeutic efficacy against CRC.

### Generation and screening of ULTR-p38i

**Fig. 2:**
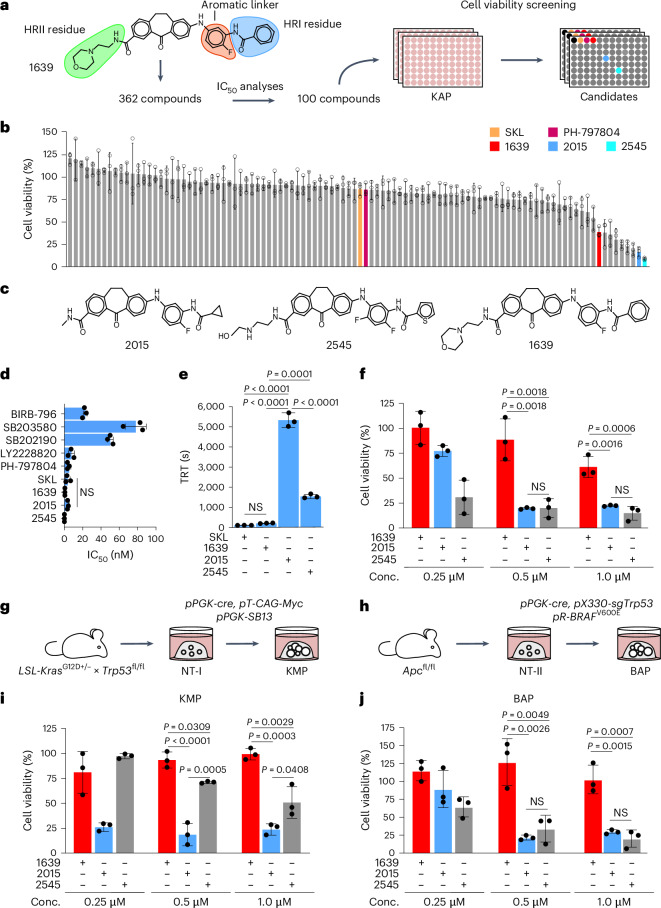
Generation and screening of ULTR-p38i. **a**, Generation of type 1.5 inhibitors and compound screen in KAP organoids. **b**, Cell viability analysis upon 4 days of treatment with the compound library or DMSO (compound concentration = 1 µM; *n* = 3 biologically independent experiments; data are presented as the mean ± s.d.). **c**, Chemical structures of 2015 and 2545 in comparison to 1639. **d**, Quantification of IC_50_ values of different p38α inhibitors on isolated protein (*n* = 3 independent ELISA assays; data are presented as the mean ± s.d.). Statistical significance was calculated using a two-tailed Student’s *t*-test. **e**, Quantification of TRTs of SKL, 1639, 2015 and 2545 (*n* = 3 independent TRT assays; data are presented as the mean ± s.d.). Statistical significance was calculated using an ANOVA and Tukey’s multiple-comparisons test (*P* ≤ 0.0001). **f**, Cell viability analysis in KAP organoids upon 4 days of treatment with 1639, 2015, 2545 or DMSO (*n* = 3 biologically independent experiments; data are presented as the mean ± s.d.). Statistical significance was calculated using an ANOVA and Tukey’s multiple-comparisons test (*P* < 0.005). **g**,**h**, Generation of KMP (**g**) and BAP (**h**) organoids. **i**, Cell viability analysis in KMP organoids upon 4 days of treatment with 1639, 2015, 2545 or DMSO (*n* = 3 biologically independent experiments; data are presented as the mean ± s.d.). Statistical significance was calculated using an ANOVA and Tukey’s multiple-comparisons test (*P* < 0.05). **j**, Cell viability analysis in BAP organoids upon 4 days of treatment with 1639, 2015, 2545 or DMSO (*n* = 3 biologically independent experiments; data are presented as the mean ± s.d.). Statistical significance was calculated using an ANOVA and Tukey’s multiple-comparisons test (*P* < 0.005). The experiments in **b**, **d**–**f**, **i** and **j** were independently performed three times, with similar results. Source data

To enhance p38α inhibition in CRC, 1639 was used as a lead compound to develop type 1.5 p38α inhibitors with further increased TRTs. We generated derivatives of 1639 with modifications in the aromatic linker or the residues binding to HRI or HRII. Overall, 362 compounds were generated and initially tested for their ability to block p38α’s enzymatic activity. The 100 most promising compounds were selected for a drug screen in KAP organoids (Fig. 2a and Supplementary Table 1). While most compounds showed little or no therapeutic effect at 1 µM concentration, we identified eight potential inhibitors that were more efficient in inducing cell death than 1639 (Fig. 2b and Supplementary Table 2).

**Extended Data Fig. 2:**
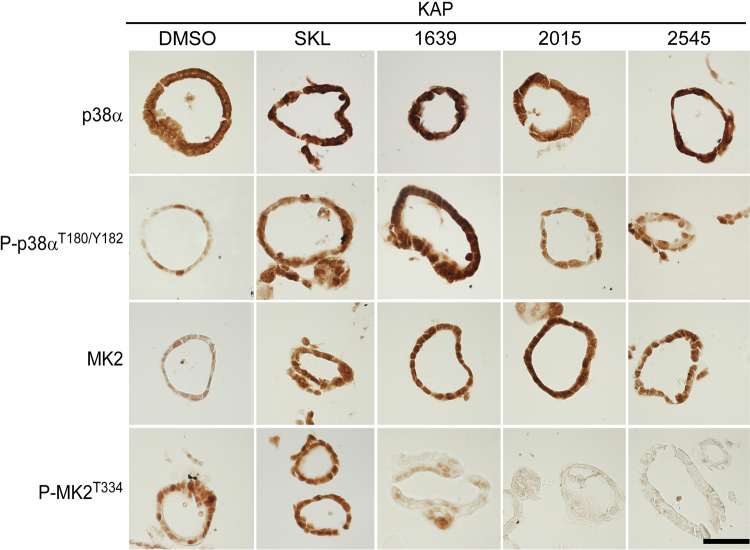
Analyzing an ULTRi-p38i therapy in CRC organoids. Representative pictures of KAP organoids stained for p38α, P-p38α^T180/Y182^, MK2 and P-MK2^T334^ after 1 d of treatment with 1 µM SKL, 1639, 2015, 2545 or DMSO (*n* = 3 cultures per condition). Scale bar, 50 µm. The experiments were independently performed twice with similar results.

The two top-scoring candidates (2015 and 2545; Fig. 2c) efficiently blocked p38α’s enzymatic activity, with IC_50_ values in the low nanomolar range (Fig. 2d), and showed strongly extended TRTs in comparison to 1639 (Fig. 2e). Compound 2015 showed an ultralong TRT of 5,270 s (*k*_off_ = 1.90 × 10^−4^ s^−1^; Fig. 2e) and was, therefore, designated as an ULTR-p38i. In validation experiments, 2015 and 2545 were administered at different concentrations to KAP organoids and both compounds showed increased cell death induction and reduced MK2^T334^ phosphorylation in comparison to 1639 (Fig. 2f and Extended Data Fig. 2).

**Extended Data Fig. 3:**
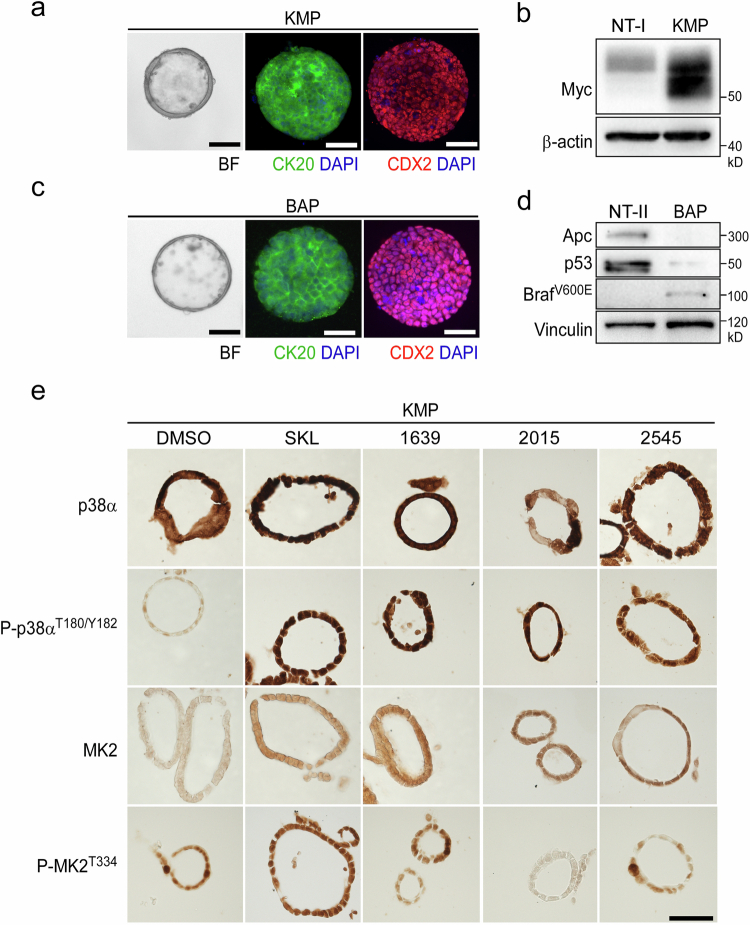
Generation of *Myc-* and *BRAF*^V600E^-driven, murine CRC organoids. **a**, Representative pictures of KMP organoids stained for CK20 and CDX2 (*n* = 2 biologically independent experiments). Scale bars, 100 µm. **b**, Representative western blot analysis in NT-I and KMP organoids (cropped blot images, *n* = 3 biologically independent experiments). **c**, Representative pictures of BAP organoids stained for CK20 and CDX2 (*n* = 2 biologically independent experiments). Scale bars, 100 µm. **d**, Representative western blot analysis in NT-II and BAP organoids (cropped blot images, *n* = 3 biologically independent experiments). **e**, Representative pictures of KMP organoids stained for p38α, P-p38α^T180/Y182^, MK2 and P-MK2^T334^ after 1 d of treatment with 1 µM SKL, 1639, 2015, 2545 or DMSO (*n* = 3 cultures per condition). Scale bar, 50 µm. The experiments in Extended Data Fig. 3a, c, e were independently performed twice and the experiments in Extended Data Fig. 3b, d were independently performed three times, all with similar results.

To validate the therapeutic potential of 2015 and 2545 in CRC organoids with different genetic backgrounds, we generated additional murine CRC organoid cultures. Organoids that were driven by overexpression of oncogenic *Myc* (*Kras*^G12D^;*Myc*^OE^;*Trp53*^−/−^; KMP) were generated by a *cre*-mediated recombination of LSL-*Kras*^G12D^ and *Trp53*^fl/fl^ alleles in NT-I colonoids, followed by a transposon-mediated integration of ectopically expressed oncogenic *Myc* (*Myc*^OE^; Fig. 2g and Extended Data Fig. 3a,b). To generate CRC organoids with a constitutive active *BRAF* mutation and a heterozygous deletion of *Trp53* (*BRAF*^V600E^;*Apc*^−/−^;*Trp53*^+/−^; BAP), we generated *Apc*^fl/fl^ (NT-II) colonoids and achieved a full loss of *Apc* by *cre*-mediated recombination and heterozygous depletion of *Trp53* by CRISPR–Cas9-mediated gene editing in this culture. Ectopically expressed *BRAF*^V600E^ was integrated through retroviral gene delivery (Fig. 2h and Extended Data Fig. 3c,d).

While cell viability assays revealed no therapeutic effects of 1639, these cultures showed increased responsiveness to 2015 and 2545 (Fig. 2i,j). In KMP organoids, 2015 showed better therapeutic effects and further reduced MK2^T334^ phosphorylation in comparison to 2545 (Fig. 2i,j and Extended Data Fig. 3e).

### ULTR-p38i are efficient in human CRCs and CRC mouse models

**Fig. 3:**
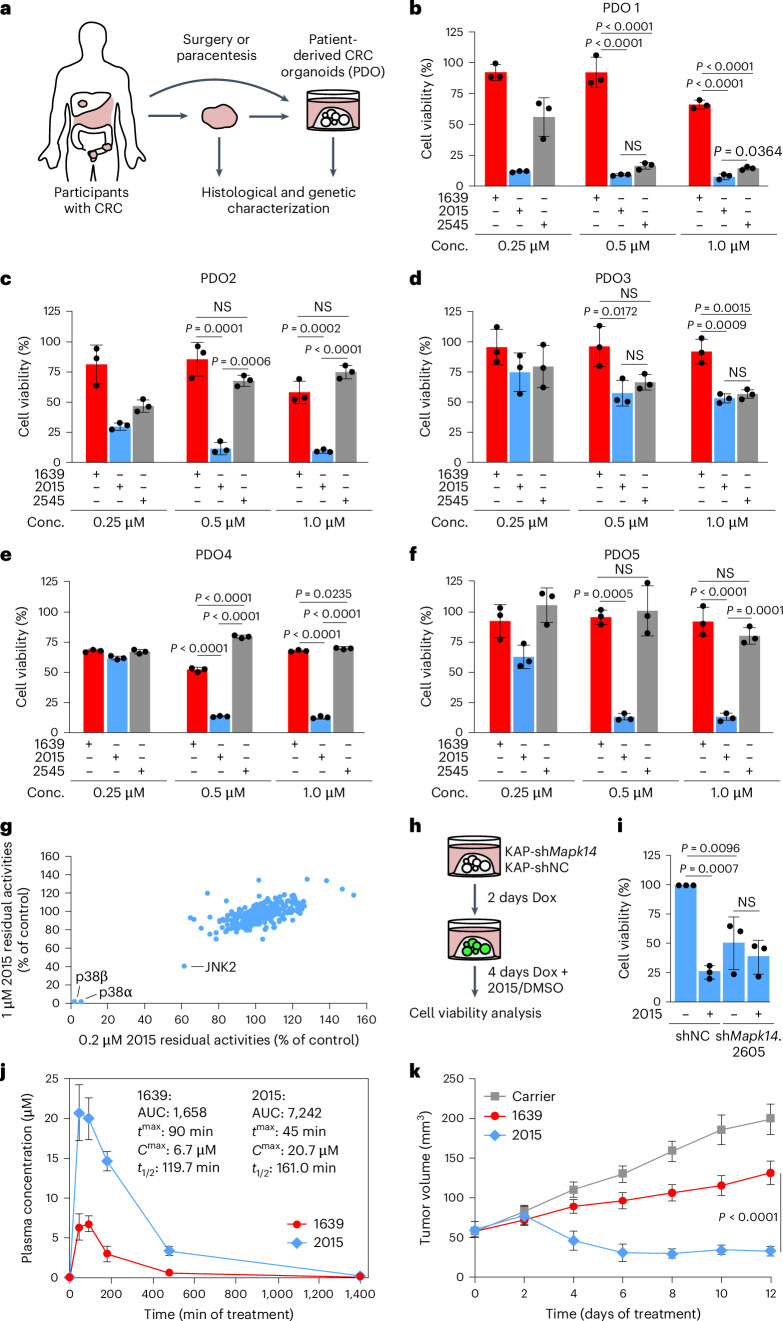
ULTR-p38i are effective in human CRC organoids and CRC mouse models. **a**, Generation of patient-derived CRC organoids from primary or secondary tumor tissue upon surgery or paracentesis. **b**–**f**, Cell viability analysis in the patient-derived organoid cultures PDO1 (**b**), PDO2 (**c**), PDO3 (**d**), PDO4 (**e**) and PDO5 (**f**) upon 4 days of treatment with 1639, 2015, 2545 or DMSO (*n* = 3 biologically independent experiments; data are presented as the mean ± s.d.). Statistical significance was calculated using an ANOVA and Tukey’s multiple-comparisons test (*P* < 0.05). **g**, Selectivity profiling of 2015 in a panel of 340 kinases (Reaction Biology Europe, compound concentration = 1 µM and 0.2 µM). **h**,**i**, Experimental setup for treatment of KAP-sh*Mapk14* and KAP-shNC organoids with 2015 or DMSO (**h**) and cell viability analysis (**i**) upon 6 days of treatment with doxycycline and 4 days of treatment with 2015 or DMSO (*n* = 3 biologically independent experiments; data are presented as the mean ± s.d.). Statistical significance was calculated using an ANOVA and Tukey’s multiple-comparisons test (*P* < 0.01). **j**, In vivo pharmacokinetic studies in mice treated with 2015 or 1639 (*n* = 3 mice per group; data are presented as the mean ± s.e.m.). **k**, Treatment of subcutaneous KAP tumors with 2015 in comparison to 1639 and carrier-treated tumors (Fig. 1j; *n* = 10 tumors per group; data are presented as the mean ± s.e.m.). Statistical significance was calculated using a two-tailed Student’s *t*-test (*P* < 0.0001). Treatment was started 1 week after organoid transplantation. The experiments in **b**–**f** and **i** were independently performed three times, with similar results. Source data

**Extended Data Fig. 4:**
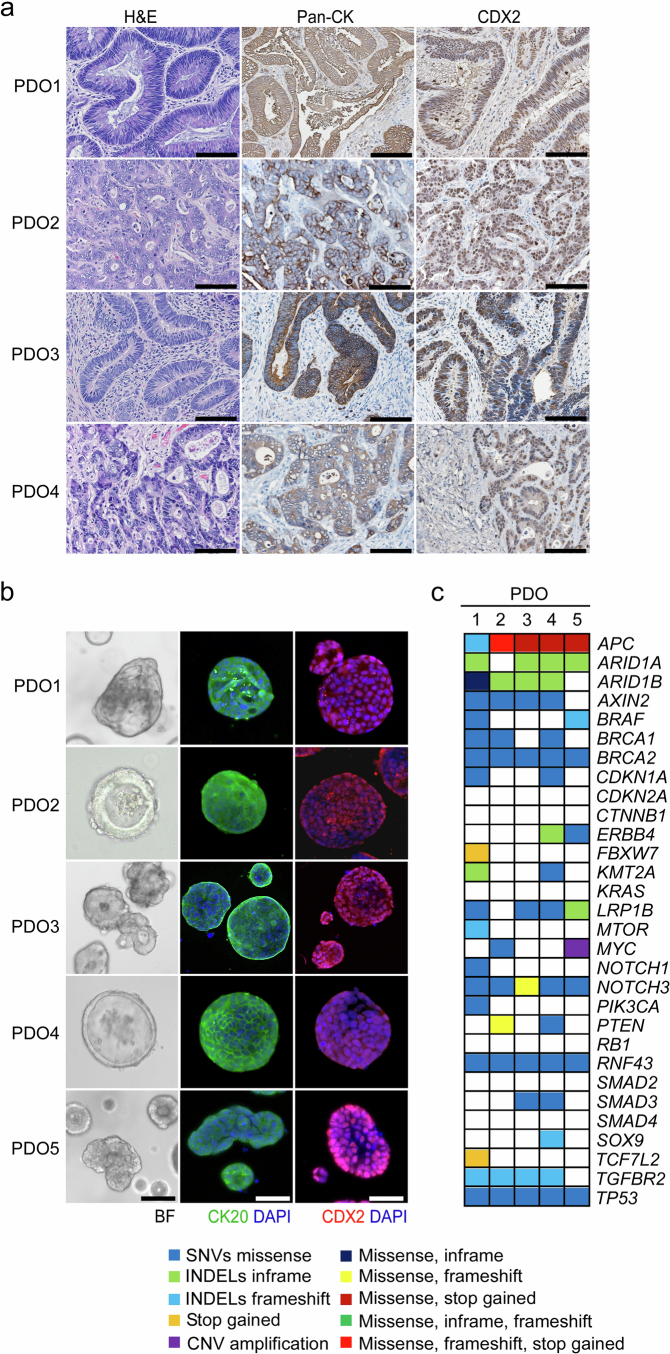
Characterization of patient-derived CRC organoids. **a**, Representative pictures of H + E and immunohistochemical staining for Pan-CK and CDX2 in human CRC tissues that were used for culture generation (PDO1-4) (*n* = 2 biologically independent experiments). Scale bars, 100 µm. **b**, Representative pictures of patient-derived CRC organoid cultures (PDO1-5) stained for CK20 and CDX2 (*n* = 2 biologically independent experiments). Scale bars, 100 µm. **c**, Panel sequencing of patient-derived CRC organoids (PDO1-5). Shown are INDELS (inframe, frameshift, stop gained), SNVs (missense, stop gained) and CNVs for selected, CRC related genes. For all data, see Supplementary Tables 4–13. The experiments in Extended Data Fig. 4a, b were independently performed twice with similar results.

To assess the potential of ULTR-p38i in human CRC, we generated and characterized human CRC organoid cultures. These cultures were derived from primary or secondary colorectal tumor tissue after rectum resection (PDO1), laparoscopic sigmoid resection (PDO3), liver metastasis resection (PDO2 and PDO4) or paracentesis (ascites in a case with peritoneal CRC metastasis; PDO5) (Fig. 3a, Supplementary Table 3 and Extended Data Fig. 4a). All tumoroid cultures showed high CK20 and CDX2 expression, even after several passages (Extended Data Fig. 4b). Analysis of single-nucleotide variants (SNVs), insertions and deletions (indels) and copy number variants (CNVs) for CRC-related genes revealed loss-of-function mutations in the *APC* and *TP53* loci of all cultures. In addition, we found high genetic variability across the different human CRC organoids. For example, we identified a *BRAF*^V600E^ mutation in PDO1, a *PTEN* frameshift mutation in PDO2 and an *MYC* amplification in PDO5 organoids (Extended Data Fig. 4c and Supplementary Tables 4–13).

Upon characterization, we tested the therapeutic effect of 2015 and 2545 in these human CRC organoid cultures by cell viability assays. While the therapeutic efficacy of 2545 differs between the different human CRC cultures, we found a pronounced therapeutic effect of 2015 in all human CRCs (Fig. 3b–f).

On the basis of these results, we selected 2015 as a frontrunner compound for further validation experiments and analyzed the selectivity profile of this compound in a panel of 340 kinases (wild-type kinase panel, Reaction Biology Europe). We found that 2015 specifically inhibited p38α and p38β, two p38 isoforms that exhibit high structural homology^30^. Apart from a moderate influence on JNK2, no other kinase was notably affected by the inhibitor (Fig. 3g and Supplementary Tables 14 and 15). To ensure that the therapeutic effect of 2015 is fully p38α dependent, we analyzed whether shRNA-mediated knockdown of *Mapk14* prevents a therapeutic response to 2015 (Fig. 3h). Indeed, we found that KAP organoids with doxycycline-induced *Mapk14*-shRNA expression (KAP-sh*Mapk14*.2605; Fig. 1b) did not respond to 2015 (Fig. 3i).

To probe the applicability of 2015 in vivo, we performed pharmacokinetic studies in mice. An oral administration of 2015 at a dose of 20 mg kg^−1^ mouse body weight resulted in a high plasma concentration (*C*^max^ = 20.7 µM) and a good stability in mice (*t*_1/2_ = 161 min; Fig. 3j).

**Extended Data Fig. 5:**
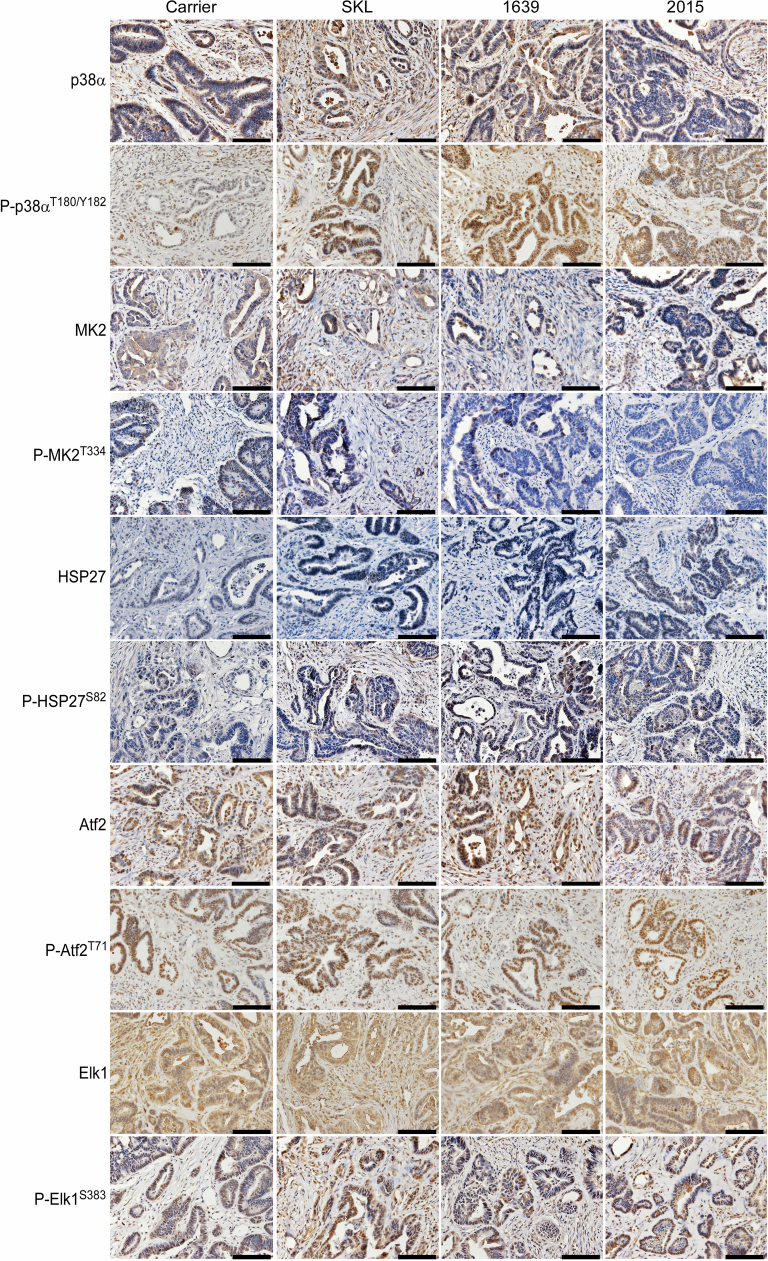
Analyzing p38α-MK2 signaling in subcutaneous CRCs upon treatment with p38i. Representative pictures of KAP subcutaneous tumors stained for p38α, P-p38α^T180/Y182^, MK2, P-MK2^T334^, HSP27, P-HSP27^S82^, Atf2, P-Atf2^T71^, Elk1, P-Elk1^S383^ after 12 d of treatment with SKL, 1639, 2015 or carrier (n = 4 tumors per group). Scale bars, 100 µm. The stainings were independently performed twice with similar results.

Subsequently, we tested 2015 in the previously described CRC mouse model of KAP organoid-based subcutaneous tumor growth. Importantly, we found that 2015 strongly reduced MK2^T334^ phosphorylation in the tumor cells in vivo (Extended Data Fig. 5) and triggered a pronounced remission of CRC (Fig. 3k). Upon continuous treatment, 2015 resulted in a stable disease in remaining tumor tissue (Fig. 3k). Overall, our data indicate that the ULTR-p38i 2015 represents a very selective and efficient therapeutic agent for the treatment of CRC.

### ULTR-p38i induce mitotic catastrophe in CRC

**Fig. 4:**
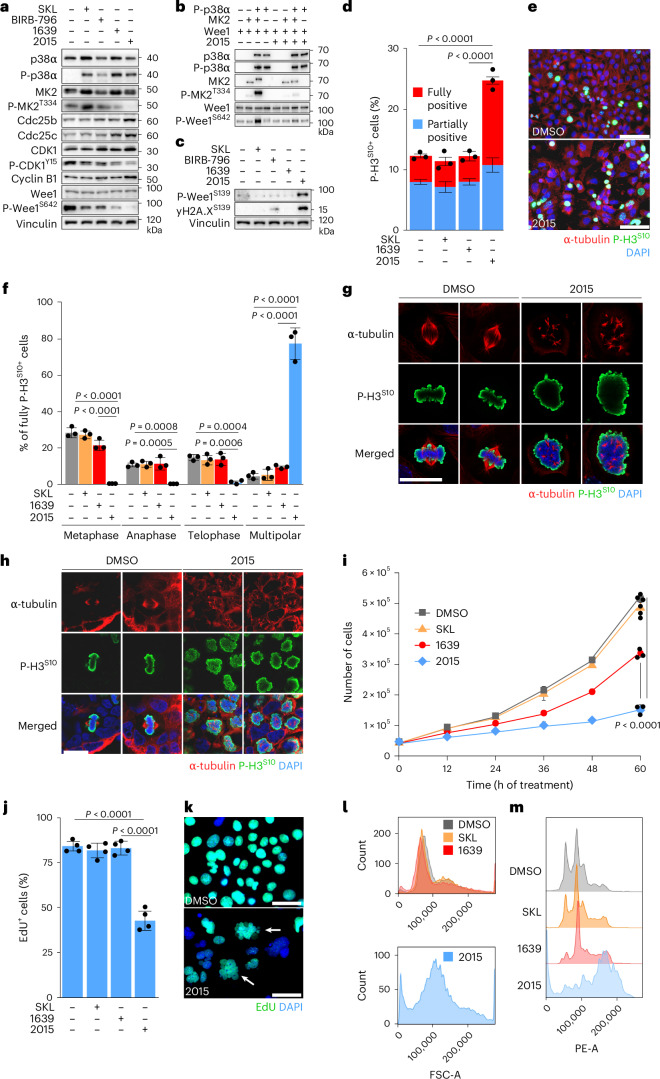
ULTR-p38i manipulate the mitotic entry and induce mitotic catastrophe in CRC. **a**, Representative western blot analysis in KAP^2D^ cells upon 1 day of treatment with 1 µM SKL, BIRB-796, 1639, 2015 or DMSO (cropped blot images; *n* = 3 biologically independent experiments). **b**, Representative western blot analysis for p38α–Wee1 kinase assay (cropped blot images; *n* = 3 independent kinase assays). **c**, Representative western blot analysis in KAP^2D^ cells upon 1 day of treatment with 1 µM SKL, BIRB-796, 1639, 2015 or DMSO (cropped blot images; *n* = 3 biologically independent experiments). **d**–**f**, Quantification (**d**), representative pictures (**e**) and determination of mitotic phases (**f**) of KAP^2D^ cells stained for P-H3^S10^ and α-tubulin after 1 day of treatment with 1 µM, SKL, 1639, 2015 or DMSO (*n* = 3 cultures per condition; data are presented as the mean ± s.d.). Statistical significance was calculated using an ANOVA and Tukey’s multiple-comparisons test (*P* < 0.001). Scale bars, 100 µm. **g**,**h**, Representative confocal microscopy pictures of stained KAP^2D^ (**g**) and PDO5 (**h**) after 1 day of treatment with 1 µM 2015 or DMSO (*n* = 3 cultures per condition). Scale bars, 15 µm. **i**, Quantification of viable KAP^2D^ upon treatment with 1 µM SKL, 1639, 2015 or DMSO (*n* = 3 cultures per condition; data are presented as the mean ± s.d.). Statistical significance was calculated using an ANOVA and Tukey’s multiple-comparisons test (*P* < 0.0001). **j**,**k**, Quantification (**j**) and representative pictures (**k**) of EdU-labeled KAP^2D^ after 2 days of treatment with 1 µM SKL, 1639, 2015 or DMSO (*n* = 4 cultures per condition; values represent the mean ± s.d.). Statistical significance was calculated using an ANOVA and Tukey’s multiple-comparisons test (*P* < 0.0001). Scale bars, 50 µm. **l**, Representative size analysis of KAP^2D^ upon 2 days of treatment with 1 µM SKL, 1639, 2015 or DMSO (measurements without gating; *n* = 3 cultures per condition). **m**, Representative DNA content analysis in KAP^2D^ upon 1 day of treatment with 1 µM SKL, 1639, 2015 or DMSO (gating strategy in Extended Data Fig. 6c; *n* = 3 cultures per condition). The experiments in **a**–**c** were independently performed three times and the experiments in **d**–**m** were independently performed twice, all with similar results. Source data

To understand how ULTR-p38i affect the viability of CRC cells, we analyzed p38α signaling in KAP^2D^ cells upon 24 h of treatment with 2015. While 2015 did not prevent paradoxical p38α hyperactivation, it nonetheless strongly reduced MK2^T334^ phosphorylation (Fig. 4a).

It was shown that p38α–MK2 signaling can block the cell-cycle-regulatory phosphatases Cdc25b and Cdc25c by inhibitory phosphorylation events, leading to their degradation in the cytoplasm^31–33^. Cdc25b and Cdc25c initiate the entry into mitosis by activating the cyclin dependent kinase 1 (CDK1)–cyclin B1 complex by dephosphorylation^34^. We found that 2015 increased the protein abundance of Cdc25b and Cdc25c and consequently activated CDK1 by reducing CDK1^Y15^ phosphorylation under conditions of high cyclin B1 levels (Fig. 4a).

Because CDK1^Y15^ inhibitory phosphorylation is carried out by the Wee1 kinase^35^, we also analyzed the activity of Wee1, by blotting for the activating phosphorylation site Wee1^S642^. Surprisingly, we found strongly reduced Wee1^S642^ phosphorylation upon treatment with 2015 (Fig. 4a), indicating that Wee1 represents a target of p38α signaling. Indeed, a kinase assay with recombinant MK2, Wee1 and active p38α protein confirmed that p38α–MK2 signaling can efficiently phosphorylate Wee1 at S642 and that this function can be blocked by 2015 (Fig. 4b). These data show that 2015-mediated p38α inhibition activates Cdc25b and Cdc25c and simultaneously inactivates Wee1, which results in a pronounced activation of the CDK1–cyclin B1 complex.

Upon activation of CDK1–cyclin B1, the mitotic entry is reinforced by a positive feedback loop that is induced by CDK1-mediated inhibitory phosphorylation of Wee1 (for example, at S139), which further blocks Wee1 and, in turn, activates CDK1 activity^36^. Because we found strongly increased Wee1^S139^ phosphorylation upon treatment with 2015 (Fig. 4c), our data show that this feedback loop is initiated by 2015. Overall, our data point out that p38α represents a major regulator of the mitotic entry in CRC and that this function is efficiently blocked by 2015. In line with this, we found a pronounced induction of DNA damage (increased yH2A.X^S139^ level) in CRC cells upon treatment with 2015 (Fig. 4c).

**Extended Data Fig. 6:**
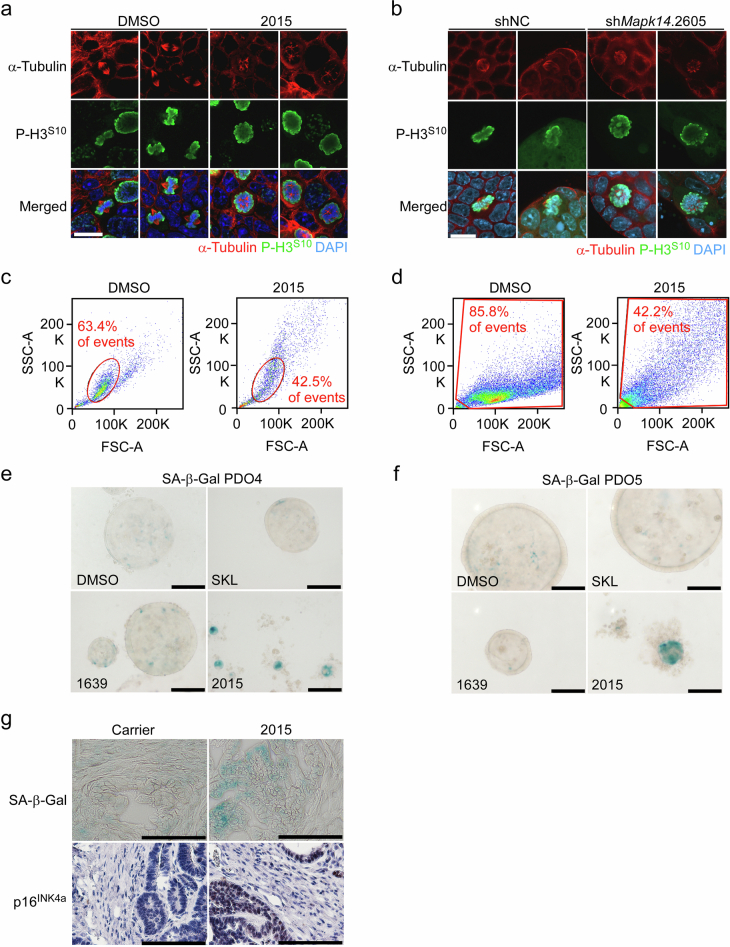
Analyzing mitotic catastrophe, DNA content, apoptosis and senescence in CRC. **a**, Representative confocal microscopy pictures of KAP organoids stained for P-H3^S10^ and α-Tubulin after 1 d of treatment with 1 µM 2015 or DMSO (*n* = 3 cultures per condition). Scale bar, 15 µm. **b**, Representative confocal microscopy pictures of KAP-sh*Mapk14*.2605 or KAP-shNC organoids stained for P-H3^S10^ and α-Tubulin after 6 d of treatment with doxycycline (*n* = 3 cultures per condition). Scale bar, 15 µm. **c**, Representative pictures of the gating strategy for FACS-based DNA content analysis in KAP^2D^ cells (see Fig. 4m, *n* = 3 cultures per condition). **d**, Representative pictures of the gating strategy for FACS-based quantification of Annexin V^+^ KAP^2D^ cells (see Fig. 5a, *n* = 3 cultures per condition). **e,f**, Representative pictures of PDO4 (**e**) and PDO5 (**f**) organoids stained for SA-β-Gal activity after 5 d of treatment with 1 µM SKL, 1639, 2015 or DMSO (*n* = 3 cultures per condition). Scale bars, 100 µm. **g**, Representative pictures of KAP subcutaneous tumors (see Fig. 3k) stained for SA-β-Gal activity and p16^INK4a^ after 12 d of treatment with 2015 or carrier (*n* = 4 tumors per group). Scale bars, 100 µm. The experiments in Extended Data Fig. 6a, b, c, d, e, f and the stainings in Extended Data Fig. 6g were independently performed twice, all with similar results.

To determine the effect of 2015 on mitosis, we stained KAP^2D^ cells under therapy for α-tubulin and the mitotic marker p-H3^S10^. We found an increased number of p-H3^S10^-positive CRC cells (especially fully positive cells) upon treatment with 2015 in comparison to cells treated with DMSO, SKL or 1639 (Fig. 4d,e). However, 2015-treated p-H3^S10^-positive cells did not show classical metaphase, anaphase and telophase characteristics as was the case with DMSO-treated or SKL-treated cells (Fig. 4e,f). In contrast, 2015 treatment induced multipolar and abnormal spindle formations (Fig. 4f,g). Confocal microscopy revealed similar severe mitotic abnormalities in 2015-treated, murine organoids (KAP) and patient-derived CRC organoids (PDO5), as well as in KAP organoids with shRNA-mediated knockdown of *Mapk14* (Fig. 4h and Extended Data Fig. 6a,b).

To explore the consequences of this mitotic catastrophe, we analyzed the cell division and replication rate of 2015-treated KAP^2D^ cells. We performed cell quantification over time (60 h) and analyzed EdU incorporation within a 24-h time period. The 2015-treated cells showed a strongly reduced proliferation rate (fold change under 2015 = 3.49, fold change under SKL = 11.66; Fig. 4i) but still a certain level of EdU incorporation (Fig. 4j,k). This indicates that these cells underwent endoreplication; in line with this, we found enlarged, EdU-positive nuclei and a strongly increased cell size of 2015-treated CRC cells (Fig. 4k,l). On the basis of these results, we performed DNA content staining in CRC cells and found strong induction of aneuploidy and polyploidy in 2015-treated cells (Fig. 4m and Extended Data Fig. 6c).

In summary, our data show that p38α represents a major regulator of the mitotic entry in CRC and that sustained inhibition of p38α leads to mitotic catastrophe, DNA damage and polyploidy in CRC cells. This effect of p38α in mitosis was found to be mediated by MK2 activation, a p38 downstream target with very high affinity for p38α (refs. ^37,38^). Consequently, only ULTR-p38i were able to effectively prevent MK2 activation upon p38α hyperactivation.

### ULTR-p38i trigger cellular senescence in CRC

**Fig. 5:**
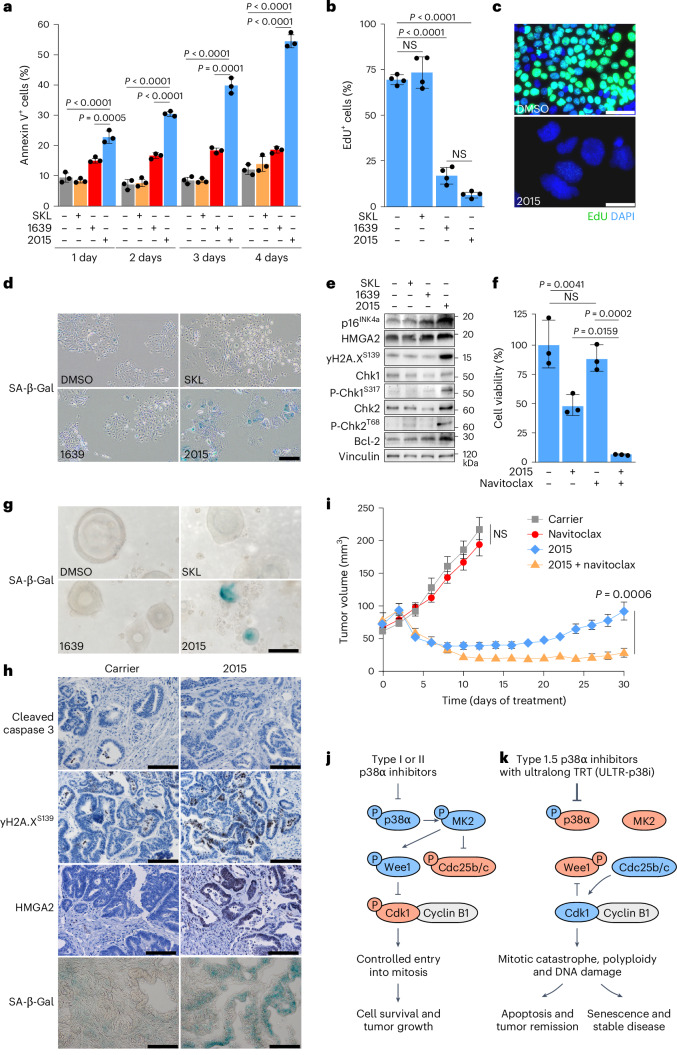
ULTR-p38i induce apoptosis and cellular senescence in CRC. **a**, Annexin V^+^ KAP^2D^ treated with SKL, 1639, 2015 or DMSO (gating strategy in Extended Data Fig. 6d; *n* = 3 cultures per condition; data are presented as the mean ± s.d.). Statistical significance was calculated using an ANOVA and Tukey’s multiple-comparisons test (*P* ≤ 0.0005). **b**,**c**, Quantification (**b**) and representative pictures (**c**) of EdU-labeled KAP^2D^ after 5 days of treatment with 1 µM SKL, 1639, 2015 or DMSO (*n* = 4 cultures per condition; data are presented as the mean ± s.d.). Statistical significance was calculated using an ANOVA and Tukey’s multiple-comparisons test (*P* < 0.0001). Scale bars, 50 µm. **d**, Representative pictures of KAP^2D^ stained for SA-β-Gal activity after 5 days of treatment with 1 µM SKL, 1639, 2015 or DMSO (*n* = 3 cultures per condition). Scale bar, 100 µm. **e**, Representative western blot analysis in KAP^2D^ upon 5 days of treatment with 1 µM SKL, 1639, 2015 or DMSO (cropped blot images; *n* = 3 biologically independent experiments). **f**, Viability of KAP organoids upon 5 days of treatment with DMSO, 0.3 µM 2015, 0.5 µM navitoclax or their combination (*n* = 3 biologically independent experiments; data are presented as the mean ± s.d.). Statistical significance was calculated using an ANOVA and Tukey’s multiple-comparisons test (*P* < 0.05). **g**, Representative pictures of PDO3 stained for SA-β-Gal activity after 5 days of treatment with 1 µM SKL, 1639, 2015 or DMSO (*n* = 3 cultures per condition). Scale bar, 50 µm. **h**, Representative pictures of stained KAP subcutaneous tumors after 12 days of treatment with 2015 or carrier (*n* = 4 tumors per group). Scale bars, 100 µm. **i**, Treatment of subcutaneous KAP tumors with 2015, navitoclax or their combination (*n* = 10 tumors per group; data are presented as the mean ± s.e.m.). Statistical significance was calculated using a two-tailed Student’s *t*-test (*P* = 0.0006). Treatment was started 1 week after organoid transplantation. **j**,**k**, Schematics of type I or II p38α inhibitor’s (**j**) or ULTR-p38i’s (**k**) mode of action. Blue, activated proteins; red, inactivated proteins. The experiments in **e** and **f** were independently performed three times, and the experiments in **a**–**d** and **g** and the stainings in **h** were independently performed twice, all with similar results. Source data

To determine the impact of mitotic catastrophe on CRC cells, we analyzed apoptosis induction by annexin V staining in KAP^2D^ cells over time. In contrast to SKL or 1639, 2015 treatment strongly increased annexin V positivity, particularly after 3–4 days of treatment (Fig. 5a and Extended Data Fig. 6d). However, a fraction of cells survived a 4-day treatment with 2015 (Fig. 5a). Because these cells showed further enlarged nuclei and no longer incorporated EdU (Fig. 5b,c), we hypothesized that these cells underwent cellular senescence. Indeed, staining for senescence-associated β-galactosidase (SA-β-Gal) activity and western blot analyses for the senescence markers p16^Ink4a^ and high-mobility group AT-hook 2 (HMGA2) revealed a clear senescence phenotype in KAP^2D^ cells that were treated over 5 days with 2015 (Fig. 5d,e). These senescent cells remained strongly positive for yH2A.X^S139^ and showed increased phosphorylation of the DNA damage response factors and cell-cycle regulators Chk1 and Chk2, indicating a DNA damage-induced cellular senescence response (Fig. 5e). Furthermore, we found upregulation of the antiapoptotic protein Bcl-2 in 2015-treated senescent cells (Fig. 5e). To determine the impact of pharmacological senolysis in these cells, we cotreated KAP organoids with 2015 and the established Bcl-2/Bcl-xl inhibitor navitoclax (ABT-263)^39^. While navitoclax alone showed no therapeutic response in KAP organoids, it efficiently killed 2015-treated, senescent cells (Fig. 5f).

To see whether senescence also occurs in 2015-treated human CRCs, we performed SA-β-Gal staining in PDOs. Importantly, we found high SA-β-Gal activity in remaining cells of all tested cultures after 5 days of treatment with 2015 (Fig. 5g and Extended Data Fig. 6e,f).

Because 2015-treated CRC mice showed stable disease after an initial tumor remission phase (Fig. 3k), we investigated whether these tumors showed a senescence phenotype as well. Histological staining of tumor samples revealed that 2015-treated tumors lacked expression of the apoptosis marker cleaved caspase 3 after a 12-day therapy. Instead, we found increased abundance of yH2A.X^S139^, HMGA2 and p16^INK4a^ and strong SA-β-Gal activity (Fig. 5h and Extended Data Fig. 6g), demonstrating a senescence phenotype in 2015-treated CRCs in vivo. A combinatorial therapy of 2015 and navitoclax further reduced subcutaneous tumor development and efficiently blocked regrowth of murine tumors (Fig. 5i).

In summary, our data show that ULTR-p38i induce apoptosis in the majority of CRC cells and senescence in cancer cells with apoptosis resistance. Treatment with 2015 manipulates the mitotic entry by inactivating Wee1 and activating Cdc25, leading to mitotic catastrophe, aneuploidy or polyploidy and DNA damage. This therapeutic effect was not seen with established type I or type II p38α inhibitors (Fig. 5j,k).

### ULTR-p38i block metastatic CRC development

**Fig. 6:**
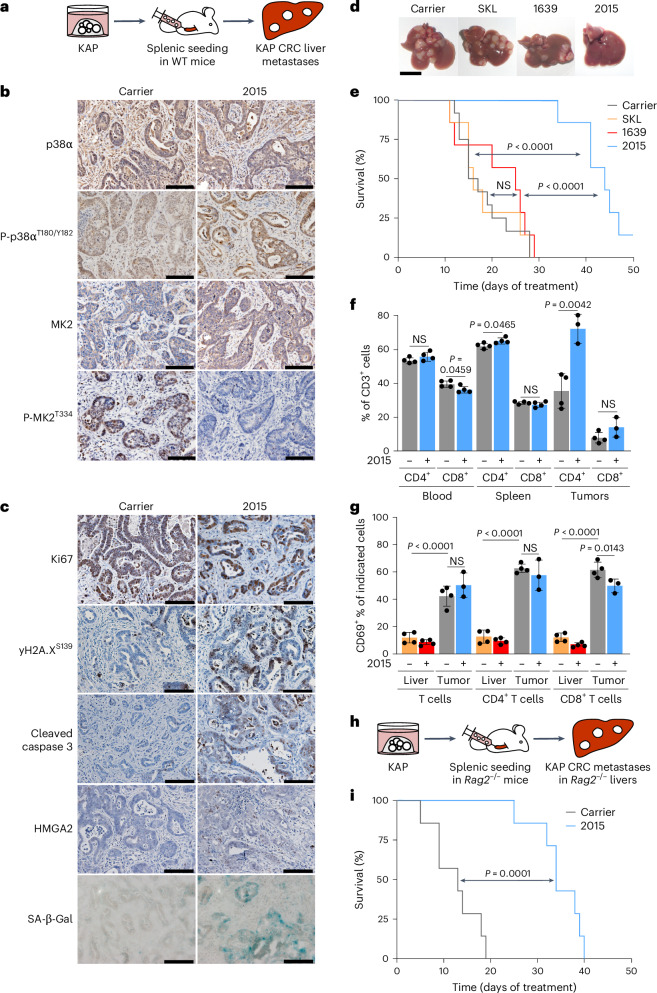
The ULTR-p38i 2015 inhibits CRC liver metastases. **a**, Generation of an organoid-based CRC liver metastasis mouse model by splenic injection of KAP organoids into WT mice. **b**, Representative pictures of KAP metastases stained for p38α, P-p38α^T180/Y182^, MK2 and P-MK2^T334^ after 4 days of treatment with 2015 or carrier (*n* = 4 mice per group). Scale bars, 100 µm. **c**, Representative pictures of KAP metastases stained for Ki67, yH2A.X^S139^, cleaved caspase 3, HMGA2 and SA-β-Gal activity after 4 days of treatment with 2015 or carrier (*n* = 4 mice per group). Scale bars, 100 µm. **d**,**e**, Representative pictures of livers (**d**) and survival analysis of mice (**e**) with KAP liver metastasis that were treated with 2015, 1639, SKL or carrier (Kaplan–Meier curve; *n* = 7 (p38α inhibitor groups) or *n* = 12 (carrier) mice). Statistical significance was calculated using a log-rank test (*P* < 0.0001). Treatment was started 5 weeks after organoid transplantation. Scale bar, 1 cm. **f**, Quantification of CD4^+^ and CD8^+^ T cells in the CD3^+^ cell fraction in blood, spleen and tumors of mice with KAP metastases upon 4 days of treatment with 2015 or carrier (determined by FACS measurements; gating strategy in Extended Data Fig. 8a; *n* = 4 (tissues) or *n* = 3 (2015-treated tumors); data are presented as the mean ± s.d.). Statistical significance was calculated using a two-tailed Student’s *t*-test (*P* < 0.05). **g**, Quantification of CD69^+^ cells in tumor-free livers or KAP tumors of mice upon 4 days of treatment with 2015 or carrier (determined by FACS measurements; gating strategy in Extended Data Fig. 8a; *n* = 4 (tissues) or *n* = 3 (2015-treated tumors); data are presented as the mean ± s.d.). Statistical significance was calculated using an ANOVA and Tukey’s multiple-comparisons test (*P* < 0.05). **h**, Generation of a KAP liver metastasis model in *Rag2*^*−/−*^ mice. **i**, Survival analysis of *Rag2*^−/−^ mice with KAP liver metastasis that were treated with 2015 or carrier (Kaplan–Meier curve; *n* = 7 mice). Statistical significance was calculated using a log-rank test (*P* = 0.0001). The stainings in **b** and **c** were independently performed twice, with similar results. Source data

**Extended Data Fig. 7:**
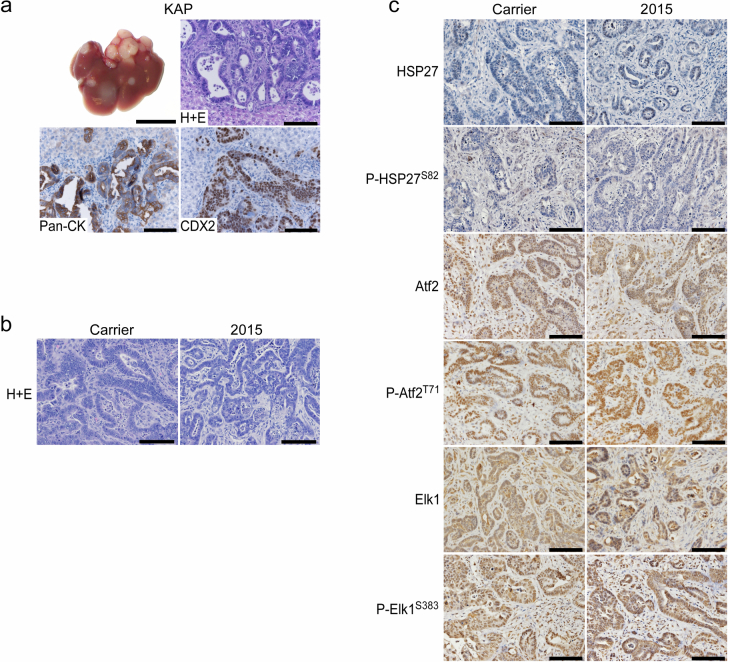
Generation and treatment of an organoid-based CRC liver metastasis mouse model. **a**, Representative pictures of liver, H + E and immunohistochemical staining for Pan-CK and CDX2 on hepatic KAP metastases (*n* = 4 mice). Scale bars, 1 cm (macroscopic picture) or 100 µm (microscopic pictures). **b**, Representative H + E stained pictures of KAP metastases after 4 d of treatment with 2015 or carrier (n = 4 mice per group). Scale bars, 100 µm. **c**, Representative pictures of KAP metastases stained for HSP27, P-HSP27^S82^, Atf2, P-Atf2^T71^, Elk1, P-Elk1^S383^ after 4 d of treatment with 2015 or carrier (n = 4 mice per group). Scale bars, 100 µm. The stainings in Extended Data Fig. 7a, b, c were independently performed twice with similar results.

To evaluate whether 2015 allows for an efficient treatment of metastatic CRC, we generated an organoid-based CRC liver metastasis model. We performed splenic injection of dissociated KAP organoids into wild-type mice, resulting in multinodular hepatic tumor development (Fig. 6a and Extended Data Fig. 7a). A histopathological analysis revealed well to moderately differentiated CRC liver metastasis (Extended Data Fig. 7a).

We commenced a 4-day treatment with 2015 in these tumors and analyzed the treatment response by immunohistological stainings. Compound 2015 efficiently reduced MK2 signaling in metastatic tumor cells while no effect on Atf2 and Elk1 was again observed (Fig. 6b and Extended Data Fig. 7b,c). Ki67 levels were only slightly reduced upon 2015 administration. However, we identified strongly increased abundance of yH2A.X^S139^ and cleaved caspase 3 in 2015-treated tumors, illustrating 2015-mediated induction of DNA damage and apoptosis in CRC metastasis. We also found elevated levels of HMGA2 and SA-β-Gal activity in a fraction of 2015-treated metastatic cells, indicating the onset of senescence (Fig. 6c).

We next performed a long-term treatment study, comparing the individual treatment responses of 2015, 1639 and SKL in metastatic CRC (starting 5 weeks after organoid inoculation). While SKL did not exert therapeutic efficacy in this model, a slight but nonsignificant survival benefit was seen upon treatment with 1639. Strikingly, 2015 strongly reduced the growth and progression of CRC metastasis and significantly increased the overall survival of tumor-bearing animals (Fig. 6d,e).

**Extended Data Fig. 8:**
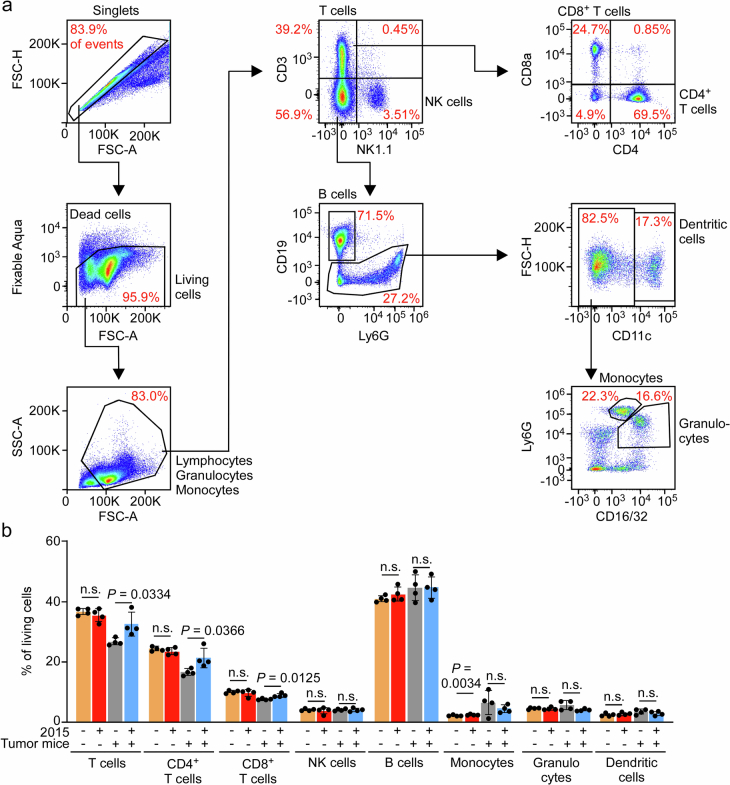
Analyzing immune cells in ULTR-p38i-treated mice. **a**, Representative pictures of the gating strategy for FACS-based quantification of immune cells (for Fig. 6f, g and Extended Data Fig. 8b, *n* = 4 tissues). **b**, Quantification of T cells, CD4^+^ T cells, CD8^+^ T cells, NK cells, B cells, monocytes, granulocytes and dendritic cells in the spleen of mice with or without KAP metastases upon 4 d of treatment with 2015 or carrier (determined by FACS measurements, gating strategy is depicted in Extended Data Fig. 8a, *n* = 4 tissues, data are presented as mean values +/- SD, statistical significance was calculated using two-tailed Student´s t-test, *P* < 0.05). Source data

Because p38α has a substantial impact on the immune system^16,40,41^, we next analyzed the effect of the ULTR-p38i 2015 on immune cells. We treated mice with and without liver metastases for 4 days with 2015 or carrier and subsequently performed detailed fluorescence-activated cell sorting (FACS) analyses (Extended Data Fig. 8a). By analyzing immune cell proportions in the spleen, we found nearly no differences in the abundance of natural killer (NK) cells, B cells, monocytes, granulocytes and dendritic cells. However, an increased number of T cells (particularly CD4^+^ T cells) was detected in the spleen of 2015-treated tumor-bearing mice (Extended Data Fig. 8b).

We therefore sought to analyze T cell abundance in more detail and compared the number of CD4^+^ and CD8^+^ T cells in the CD3^+^ cell fraction of the blood, the spleen and the tumors. Interestingly, we found strongly increased infiltration of CD4^+^ T cells in CRC liver metastases (Fig. 6f). The majority of these tumor-infiltrating CD4^+^ T cells were activated (CD69^+^); however, the activation was not influenced by 2015 (Fig. 6g). These data show that 2015 increases the number of CD4^+^ T cells in CRC liver metastases and modifies the immune system of mice.

To determine the impact of the immune system on the treatment response, we performed a treatment study in *Rag2*^−/−^ C57BL/6 mice that lack mature T cells or B cells^42^ (Fig. 6h). A strong treatment response in immunodeficient mice revealed that the therapeutic effect of 2015 in CRC is mainly mediated by a cell-intrinsic effect and induction of mitotic catastrophe (Fig. 6i).

### ULTR-p38i show strong activity in different CRC models

**Fig. 7:**
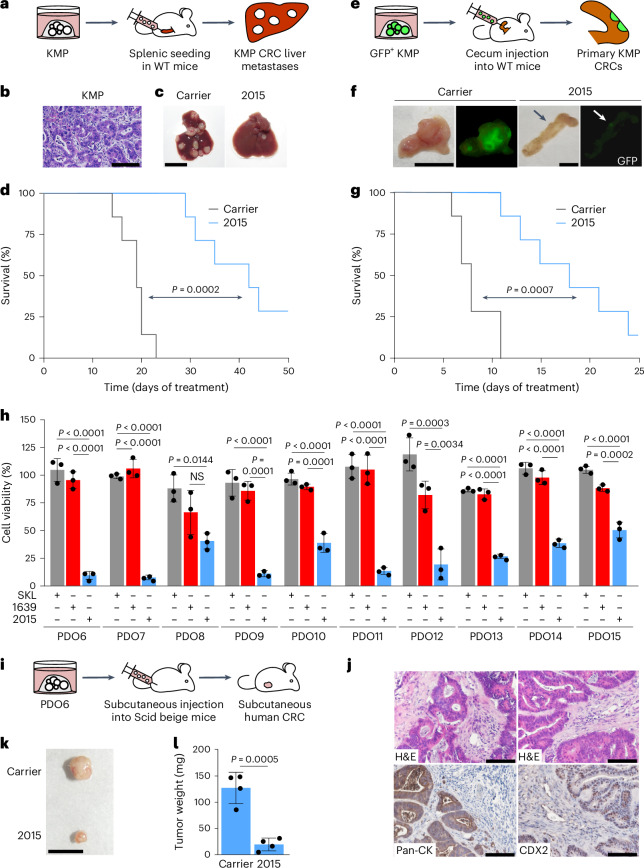
The ULTR-p38i 2015 shows strong activity in different CRC models. **a**, Generation of an KMP CRC liver metastasis mouse model. **b**, Representative picture of H&E-stained KMP metastases 27 days after tumor initiation (*n* = 4 mice). Scale bar, 100 µm. **c**,**d**, Representative pictures of livers (**c**) and survival analysis of mice (**d**) with KMP liver metastasis that were treated with 2015 or carrier (Kaplan–Meier curve; *n* = 7 mice per group). Statistical significance was calculated using a log-rank test (*P* = 0.0002). Treatment was started 1 week after organoid transplantation. Scale bar, 1 cm. **e**, Generation of an organoid-based primary CRC mouse model by injecting KMP organoids into the cecum wall of WT mice. **f**,**g**, Representative pictures of the cecum inside (**f**) and survival analysis of mice (**g**) with KMP primary CRCs that were treated with 2015 or carrier (Kaplan–Meier curve; *n* = 7 mice per group). Statistical significance was calculated using a log-rank test (*P* = 0.0007). Treatment was started 9 days after organoid transplantation. Scale bars, 1 cm. **h**, Cell viability analysis in the patient-derived organoid cultures PDO6–PDO15 upon 4 days of treatment with 1 µM SKL, 1639, 2015 or DMSO (*n* = 3 biologically independent experiments; data are presented as the mean ± s.d.). Statistical significance was calculated using an ANOVA and Tukey’s multiple-comparisons test (*P* < 0.05). **i**, Generation of a human CRC mouse model based on subcutaneous injection of PDO6 into CB17 Scid beige mice. **j**, Representative pictures of H&E and immunohistochemical staining for pan-CK and CDX2 in subcutaneous PDO6 CRC 14 weeks after tumor initiation (*n* = 4 tumors per group). Scale bars, 100 µm. **k**,**l**, Representative pictures (**k**) and weight (**l**) of subcutaneous human PDO6 CRCs that were treated with 2015 or carrier (*n* = 4 tumors per group; data are presented as the mean ± s.d.). Statistical significance was calculated using a two-tailed Student’s *t*-test (*P* = 0.0005). Treatment was started 10 weeks after organoid transplantation. Scale bar, 1 cm. The experiments in **h** were independently performed three times and the stainings in **b** and **j** were independently performed twice, all with similar results. Source data

**Extended Data Fig. 9:**
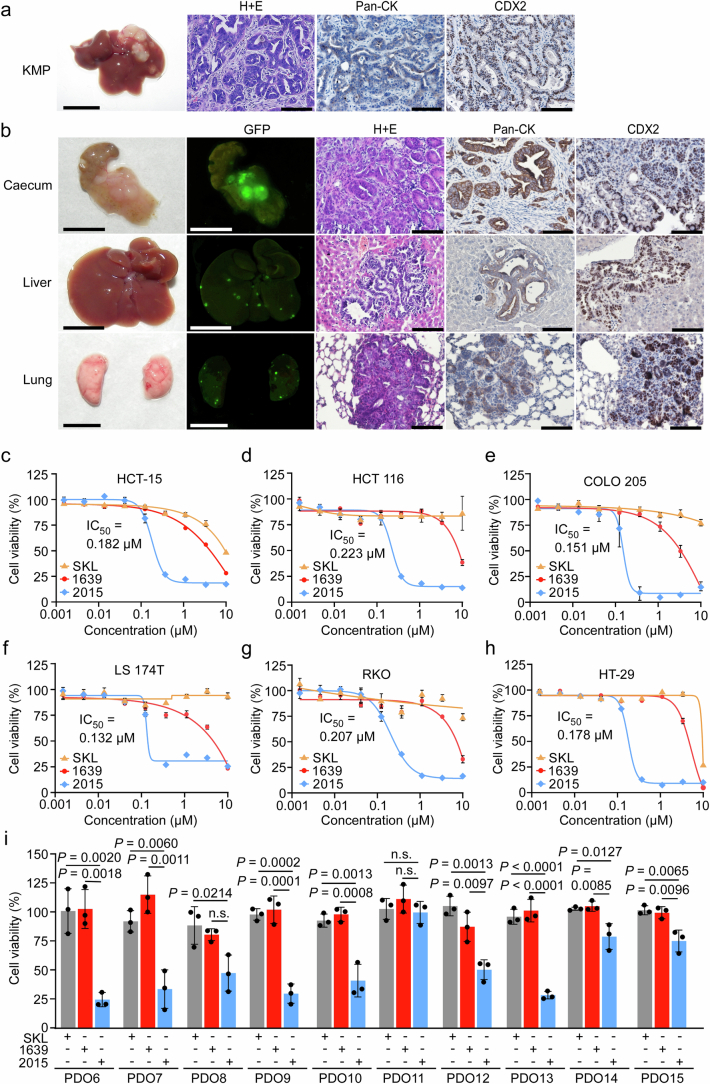
Generation of *Myc*-driven CRC mouse models and treatment of human CRC cultures. **a**, Representative pictures of hepatic KMP metastases and H + E and immunohistochemical staining for Pan-CK and CDX2 on tumor tissue (*n* = 4 mice). Scale bars, 1 cm (macroscopic picture) or 100 µm (microscopic pictures). **b**, Representative pictures of KMP primary tumors in the caecum and KMP liver and lung metastases (with GFP imaging of tumors) and H + E and immunohistochemical staining for Pan-CK and CDX2 on tumor tissues (*n* = 4 mice). Scale bars, 1 cm (macroscopic pictures) or 100 µm (microscopic pictures). **c-h**, Cell viability analysis in the human CRC cell lines HCT-15 (**c**), HCT 116 (**d**), COLO 205 (**e**), LS 174 T (**f**), RKO (**g**) and HT-29 (**h**) upon 4 d of treatment with SKL, 1639, 2015 or DMSO (drug response curves and IC_50_ values, data are presented as mean values +/- SD, *n* = 3 cultures per condition). **i**, Cell viability analysis in the patient-derived organoid cultures PDO6 – PDO15 upon 4 d of treatment with 0.5 µM of SKL, 1639, 2015 or DMSO (*n* = 3 biologically independent experiments, data are presented as mean values +/- SD, statistical significance was calculated using an ANOVA and Tukey’s multiple comparisons test, *P* < 0.05). The experiments in Extended Data Fig. 9c–h and the stainings in Extended Data Fig. 9a, b were independently performed twice, the experiments in Extended Data Fig. 9i were independently performed three times, all with similar results. Source data

To determine the treatment response of 2015 in highly aggressive CRC, we generated a mouse model of *Myc*-driven metastatic CRC. We performed splenic seeding of KMP organoids into wild-type mice (Fig. 7a), resulting in multinodular hepatic metastases that developed faster, showed a less differentiated phenotype (moderate to poor differentiation) and had a reduced stroma compartment when compared to KAP metastasis (Fig. 7b and Extended Data Figs. 7a and 9a). Importantly, *Myc*-driven tumors also showed a strong treatment response toward 2015, resulting in significantly prolonged survival of treated mice in a long-term treatment study (Fig. 7c,d).

To determine whether 2015 also allows for the treatment of primary CRC, we injected GFP^+^ KMP organoids into the cecum wall of wild-type mice (Fig. 7e). This injection rapidly triggered a multinodular CRC development that infiltrated the mucosa, the submucosa and the muscular layer of the cecum (Extended Data Fig. 9b). Furthermore, we found formation of GFP^+^ CRC liver metastases in 57% and lung metastases in 29% of mice (Extended Data Fig. 9b). We next performed a treatment study with 2015 in this model (starting 9 days after organoid injection) and found a strong reduction in tumor development and a significantly prolonged survival of tumor-bearing animals upon 2015 therapy (Fig. 7f,g).

To further assess the robustness and generality of the ULTR-p38i therapy in persons with CRC, we explored the effects of 2015 in additional human CRC cultures. We treated established human CRC cell lines (HCT-15, HCT 116, COLO 205, LS 174T, RKO and HT-29) and generated drug–response curves. While SKL showed no or only low therapeutic effects and 1639 could only slightly increase CRC cell death, 2015 strongly reduced cell viability in all tested cell lines (Extended Data Fig. 9c–h). Subsequently, we performed a 2015 treatment study in an additional set of human CRC organoids (PDO6–PDO15) that were generated from participants at the Robert Bosch Hospital (Stuttgart). We found a significant therapeutic effect of 2015 in all cultures (Fig. 7h and Extended Data Fig. 9i).

Lastly, we sought to treat PDO-derived CRCs with 2015 in vivo. We injected PDO6, driven by *KRAS*^Q61H^, *PIK3CA*^N345I^ and *APC*^G1288*^ mutations (*TP53* status = wild type), into the flanks of immunodeficient CB17 Scid beige mice, resulting in the development of tumors that resemble moderately differentiated human CRC (Fig. 7i,j). Upon tumor formation (10 weeks after organoid inoculation), these mice were treated for 4 weeks with 2015 or carrier and we found a strong reduction in tumor size upon 2015 therapy (Fig. 7k,l).

Overall, our data show that 2015 allows for efficient treatment of histologically and genetically diverse CRC metastasis, primary CRCs and human CRC in vitro and in vivo.

### ULTR-p38i have no adverse effects on mice

**Fig. 8:**
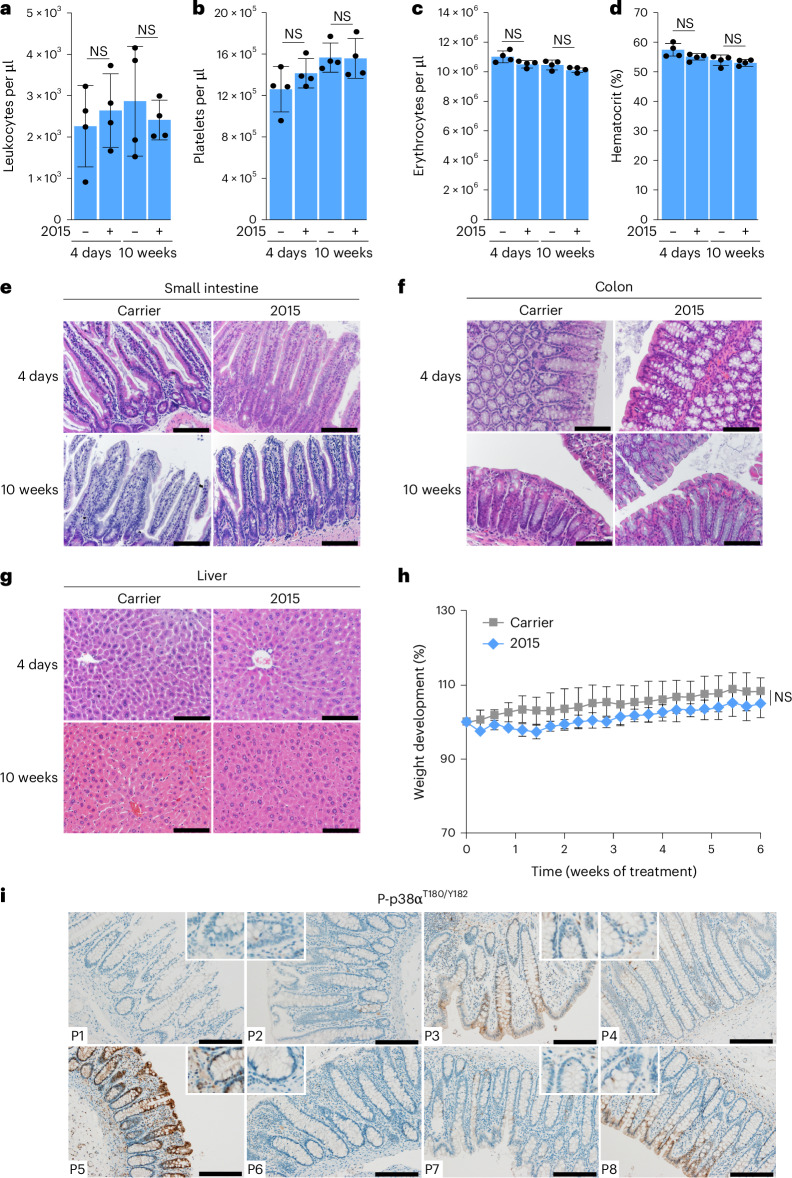
The ULTR-p38i 2015 is well tolerated by mice. **a**–**d**, Quantification of leukocytes (**a**), platelets (**b**), erythrocytes (**c**) and the hematocrit (**d**) in mice treated for 4 days or 10 weeks with 2015 or carrier (*n* = 4 mice per group; data are presented as the mean ± s.d.). Statistical significance was calculated using a two-tailed Student’s *t*-test. **e**,**f**, Representative pictures of H&E-stained small intestine (**e**) and colon (**f**) of mice treated for 4 days or 10 weeks with 2015 or carrier (*n* = 4 mice per group). Scale bars, 100 µm. **g**, Representative pictures of H&E-stained liver tissue in mice treated for 4 days or 10 weeks with 2015 or carrier (*n* = 4 mice per group). Scale bars, 100 µm. **h**, Weight development of mice treated with 2015 or carrier (*n* = 7 mice per group; data are presented as the mean ± s.d.). Statistical significance was calculated using a two-tailed Student’s *t*-test. **i**, Representative pictures of colon tissue of participants (*n* = 8) stained for P-p38α^T180/Y182^. Scale bars, 200 µm. The stainings in **e**–**g** and **i** were independently performed twice, with similar results. Source data

**Extended Data Fig. 10:**
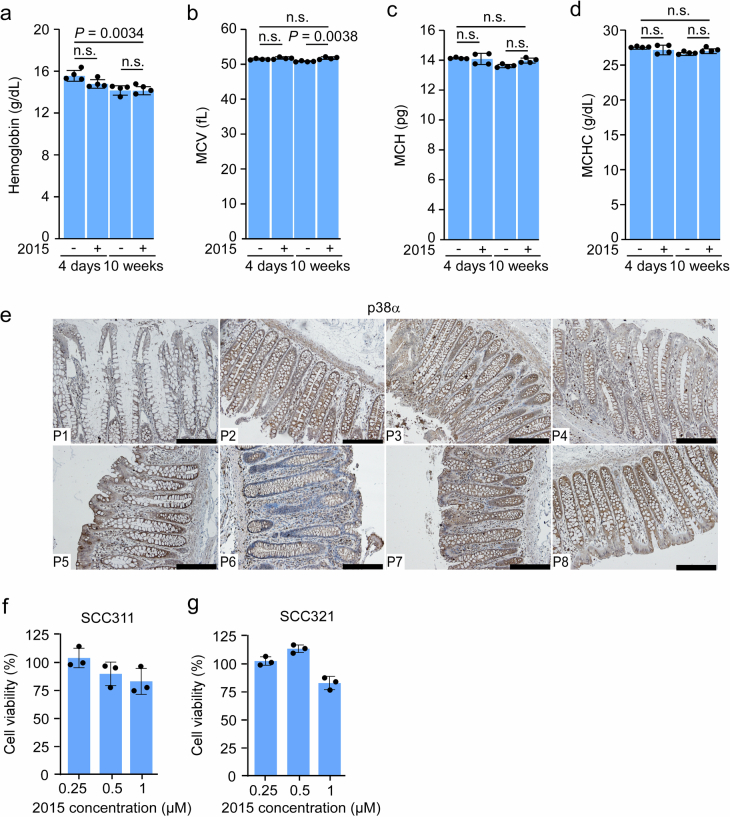
Analyzing the effect of ULTR-p38i on healthy tissues in mice. **a**, Hemoglobin concentration in mice treated for 4 d or 10w with 2015 or carrier (*n* = 4 mice per group, data are presented as mean values +/- SD, statistical significance was calculated using an ANOVA and Tukey’s multiple comparisons test, *P* = 0.0034). **b-d**, Determination of the mean corpuscular volume (MCV, **b**), the mean corpuscular hemoglobin (MCH, **c**) and the mean corpuscular haemoglobin concentration (MCHC, **d**) in mice treated for 4 d or 10w with 2015 or carrier (*n* = 4 mice per group, data are presented as mean values +/- SD, statistical significance was calculated using an ANOVA and Tukey’s multiple comparisons test, *P* < 0.005). **e**, Representative pictures of p38α stained colon tissue of patients (*n* = 8 patients). Scale bars, 200 µm. **f,g**, Cell viability analysis in SCC311 (**f**) and SCC321 (**g**) human colon organoids upon 4 d of treatment with 2015 or DMSO (*n* = 3 biologically independent experiments, data are presented as mean values +/- SD). The experiments in Extended Data Fig. 10f, g were independently performed three times and the stainings in Extended Data Fig. 10e were independently performed twice, all with similar results. Source data

To explore the translational potential of a therapy with 2015, we analyzed the effect of 2015 on healthy regenerating tissues and analyzed blood cells and gastrointestinal tissues after short-term and long-term treatment. We conducted a daily treatment of wild-type mice with 2015 or carrier for either 4 days or 10 weeks and subsequently quantified leukocytes, platelets and erythrocytes. Furthermore, we determined the hematocrit and the hemoglobin concentration and calculated routinely used red blood cell indices (mean corpuscular volume (MCV), mean corpuscular hemoglobin (MCH) and mean corpuscular hemoglobin concentration (MCHC)). Importantly, we found no relevant differences between 2015-treated and carrier-treated animals in all cellular fractions and detected no signs of anemia (Fig. 8a–d and Extended Data Fig. 10a–d). Histological evaluations of intestinal tissues revealed a normal architecture of small intestine and colon without any signs of intestinal mucosal atrophy, indicating that 2015 treatment did not influence the regeneration of intestinal epithelium (Fig. 8e,f).

To exclude any liver toxicity (which was reported upon treatment with first-generation p38α inhibitors^43^), we also analyzed the livers of mice upon 2015 short-term and long-term treatment. Importantly, 2015 did not affect the murine liver architecture and no signs for liver damage (inflammation, necrosis, cholestasis, steatosis or fibrosis) were seen after 2015 treatment (Fig. 8g). Overall, 2015-treated mice showed a normal behavior and did not lose body weight over time (Fig. 8h).

To assess whether the healthy colonic mucosa of participants might be affected by a ULTR-p38i therapy, we histologically analyzed p38 activation in human colon tissues. We stained healthy colon areas from eight different participants for p38α and P-p38α^T180/Y182^. While we found p38α activation at the luminal end of the colonic crypts in some participants (P3, P5 and P8), the base of the crypts, which is the area of regeneration of the intestinal epithelium^44^, was always P-p38α^T180/Y182^ negative (Fig. 8i and Extended Data Fig. 10e). These data indicate that the regeneration of colonic crypts is not controlled by p38α. To test whether healthy colonic PDOs are sensitive to ULTR-p38i, we treated two healthy human colon cultures with 2015. Importantly, both cultures showed only a very weak response to 2015 (Extended Data Fig. 10f,g), indicating that proliferating colon cells are much less sensitive to ULTR-p38i than tumor cells.

In summary, our data show a strong translational potential of ULTR-p38i such as 2015 for cancer therapies.

## Discussion

Here, we described the development of type 1.5 ULTR-p38i for the treatment of CRC. In contrast to previously described type I or type II p38α inhibitors, ULTR-p38i show unprecedented therapeutic effects in syngeneic CRC mouse models and patient-derived CRC organoids, already as a monotherapy.

The limited activity of small-molecule inhibitors against active targets often impedes the efficacy of targeted therapies in cancer. To improve the activity of pharmacological cancer drugs, the residence time of small molecules has become an important subject of cancer research over the past years. Thus, important advances were made with the development of next-generation inhibitors that bind covalently to their target, thereby allowing for sustained inhibition^45^. For example, the covalent mutant-selective EGFR inhibitor osimertinib showed striking therapeutic effects in *EGFR*-mutated lung cancer, which were less pronounced with reversible, first-generation EGFR inhibitors such as gefitinib and erlotinib^46^. Furthermore, efficient covalent inhibitors targeting Bruton’s tyrosine kinase were developed and successfully used for the treatment of different tumor entities^46^.

Although better tools that facilitate the design of efficient covalent inhibitors have emerged^46^, the development of efficient and specific covalent binders remains challenging and is not feasible for all kinases that represent promising targets for cancer therapies. Thus, covalent inhibitors are dependent on the availability of suitable binding residues (mainly cysteine residues) adjacent to the ATP-binding sites^45^. In fact, p38α and many other kinases lack available cysteine residues that allow for an interaction with actual covalent inhibitors^47^. Furthermore, the reactive structure of these inhibitors may cause adverse effects^45^.

We here report potent and specific kinase inhibitors that achieve ultralong TRTs without covalent binding to their target. These compounds show strong therapeutic effects without toxicity and may represent a promising therapeutic concept for cancer targets that are not druggable by covalent inhibitors. Hence, we believe that the concept of type 1.5 ultralong-TRT kinase inhibitors could be rolled out to other targets and suggest clinical trials for testing ULTR-p38i in persons suffering from advanced CRC. Because of the identified role of p38α as a general mitotic regulator, we also propose testing ULTR-p38i in other therapy-resistant tumor entities.

## Methods

### Ethical regulations

The research performed in this study complies with all relevant ethical regulations. The experiments with patient-derived CRC organoids were approved by the responsible ethics committee of the Medical Faculty of the University and the University Hospital Tübingen (ethic approval numbers 949/2021BO2, 117/2020BO1 and 696/2016BO2). All participants were deidentified and gave their written informed consent according to German law. The animal experiments in this study were approved by committees of the regional authority of the state of Baden-Wuerttemberg (Regierungspraesidium Tübingen, authorization number M17/18G).

### Vector design

Plasmids encoding *Cas9n* and gRNAs targeting *Apc* (*pX330.sgApc.2*) or *Trp53* (*pX330.sgTrp53*) were previously described and kindly provided by W. Xue and T. Jacks^48,49^. Transposon-based gene delivery of *Myc* was carried out by the *Myc*-encoding transposon plasmid pT-CAG-Myc (pT-CaMIG) and the *Sleeping Beauty transposase 13*-encoding plasmid pSB13 (refs. ^50–52^). Retroviral delivery of *BRAF*^V600E^ was performed with pR-BRAF^V600E^-Puro and *cre*-mediated gene editing with pPGK-cre (refs. ^50–52^). Tetracycline-inducible shRNA expression was achieved by using the retroviral vector pRT3GEPIR (ref. ^53^) that was kindly provided by J. Zuber. *Mapk14* shRNAs were designed using splashRNA^54^ and ordered as full-length oligos from Sigma-Aldrich. They were cloned into pRT3GEPIR using PCR with XhoI or EcoRI restriction sites. The shNC was described previously^22,52^. Plasmids were analyzed using SnapGene Viewer version 4.2.4. All primer, gRNA and shRNA sequences are listed in Supplementary Table 16.

### Murine CRC organoids

Murine colon organoids were isolated from 10-week-old female B6.129-*Kras*^*tm4Tyj*^ *Trp53*^*tm1Brn*^/J (NT-I) and C57BL/6-*Apc*^*tm1Tyj*^/J (*Apc*^fl/fl^) (NT-II) mice (obtained from Charles River), using established protocols^55^. The colon fragments were washed with ice-cold DPBS, transferred to 2 mM EDTA chelating buffer and incubated for 30–60 min at 4 °C on an orbital shaker. The supernatant was removed and the tissue fragments were resuspended in DPBS containing 10% FBS. Crypts were filtered (70 µm), centrifuged (300*g*, 5 min) and embedded in 50-µl domes of growth factor reduced (GFR) Matrigel (Corning). Following polymerization at 37 °C, the domes were covered with murine colon medium (Supplementary Table 17), supplemented with 10.5 µM Y-27632 dihydrochloride (Invitrogen).

Organoids were cultured at 37 °C and 7% CO_2_ in murine colon medium that was refreshed every 2–3 days and split in a ratio of 1:2 to 1:6 (once or twice per week) by mechanical dissociation.

For transfection, organoids were mechanically dissociated and digested using TrypLE with 100 µg ml^−1^ DNaseI and 10.5 µM Y-27632 dihydrochloride (Invitrogen) at 37 °C. The single cells were resuspended in 450 µl of murine colon medium supplemented with 10.5 µM Y-27632 dihydrochloride (Invitrogen) and plated in 24-well plates. Lipofectamine 2000 (Invitrogen) transfection was performed according to the manufacturers’ protocol using 1.5 µg of pPGK-cre, pX330 and pT-CaMIG or 0.3 µg of pSB13 plasmid DNA. After 6 h of incubation at 37 °C, organoids were plated in Matrigel and cultured with murine colon medium, supplemented with 10.5 µM Y-27632 dihydrochloride (Invitrogen). Selection of transfected organoids was performed by withdrawal of recombinant R-spondin-1, EGF and Wnt-3a (Extended Data Fig. 1c). Genetic modifications were determined by western blot or PCR (primer sequences in Supplementary Table 16) and the cultures were also authenticated by PCR (*Kras* locus, *Trp53* locus, *Myc* transposon and *Braf*^V600E^ complementary DNA).

### Patient-derived CRC organoids

Human CRC organoids were isolated from the tumor tissues or peritoneal fluid of adult participants from the University Hospital Tübingen or the Robert Bosch Hospital Stuttgart. There were no restrictions regarding participant selection and no bias based on sex and gender. Because CRC is a critical disease in the male and female populations, organoid cultures from male and female participants were established and applied for the treatment studies. The cultures were established from participants with CRC with the following sex and age: PDO1, male, 69 years; PDO2, female, 70 years; PDO3, male, 62 years; PDO4, female, 51 years; PDO5, male, 42 years; PDO6, male, 58 years; PDO7, male, 36 years; PDO8, female, 36 years; PDO9, male, 67 years; PDO10, male, 52 years; PDO11, female, 50 years; PDO12, female, 70 years; PDO13, female, 82 years; PDO14, male, 69 years; PDO15, female, 71 years. Because the treatment responses of the cultures were analyzed individually, no sex-based and gender-based analyses were performed.

Primary tumor material was chopped, washed in ice-cold medium and transferred into basic medium (Supplementary Table 17), supplemented with 2.5 mg ml^−1^ collagenase II (Thermo Fisher Scientific), 2.5 mg ml^−1^ collagenase XI (Sigma-Aldrich), 1 mg ml^−1^ Dispase II (Sigma-Aldrich), 100 µg ml^−1^ DNase I and 10.5 µM Y-27632 dihydrochloride (Invitrogen). Tumor digestion was performed at 37 °C and stopped by adding medium containing 10% FBS. The mixture was centrifuged for 5 min at 300*g*. Red blood cells were removed with ACK lysing buffer (Thermo Fisher Scientific; 5 min at room temperature) and the pellet was resuspended in medium. Approximately 1,000 cells were resuspended and plated in 50 µl of Cultrex RGF BME, type 2 (R&D Systems). The ascites material was pelleted by centrifugation at 300*g* and washed three times with ice-cold PBS. Human CRC organoids were cultured in human CRC medium (Supplementary Table 17) at 37 °C and 7% CO_2_. Within the first week, the medium was supplemented with 10.5 µM Y-27632 dihydrochloride (Invitrogen). The human colon organoids (SCC311 and SCC321) were purchased from Sigma-Aldrich and cultured in human colon medium (Supplementary Table 17) at 37 °C and 7% CO_2_. They were derived from participants with the following sex and age: SCC311, female, 52 years; SCC321, male, 21 years.

### Two-dimensional cell culture and retroviral gene delivery

To generate KAP^2D^ cells, KAP organoids were isolated from Matrigel, fragmented by mechanical dissociation and plated without any extracellular matrix on common cell culture dishes. Phoenix-Eco packaging cells (CRL-3214), HCT-15 (CCL-225), HCT 116 (CCL-247), COLO 205 (CCL-222), LS 174T (CL-188), RKO (CRL-2577) and HT-29 (HTB-38) were obtained from the American Type Culture Collection. The cells were cultured at 37 °C and 7% CO_2_ with RPMI (KAP^2D^ cells) or DMEM (other cell lines), supplemented with 10% FBS and penicillin–streptomycin. KAP^2D^ cells were authenticated using PCR (*Kras* locus and *Trp53* locus). Phoenix-Eco packaging cells and HCT-15, HCT 116, COLO 205, LS 174T, RKO and HT-29 cells were authenticated by the provider using short tandem repeat profiling. No commonly misidentified cell lines were used. *Mycoplasma* contamination was excluded by a PCR-based analysis.

To produce retroviral particles, Phoenix packaging cells were transfected with retroviral DNA (pRT3GEPIR and pR-BRAF^V600E^-Puro) through calcium phosphate transfection. The viral supernatant, supplemented with polybrene (5 µg ml^−1^) was applied on cells or dissociated organoids and the infected cultures were selected with puromycin (3 µg ml^−1^). Expression of shRNAs and GFP was induced with 5 µg ml^−1^ doxycycline (Sigma).

### In vitro treatment studies

For in vitro treatment studies, organoids were mechanically dissociated and digested using TrypLE supplemented with 100 µg ml^−1^ DNaseI and 10.5 µM Y-27632 dihydrochloride (Invitrogen) at 37 °C. A total of 1,000–2,000 single cells were plated per well in white 96-well plates (Greiner) in culture medium containing 10% GFR Matrigel or Cultrex BME. The treatment with doxycycline (5 µg ml^−1^), pharmacological inhibitors or corresponding volumes of DMSO was started 24 h after plating. Doxycycline was obtained from Sigma-Aldrich. PH-797804, LY2228820, BIRB-796, SB203580, SB202190 and navitoclax were obtained from Selleckchem. Cell viability was measured by means of CellTiter-Glo 3D or CellTiter-Glo 2.0 reagent (Promega) according to the manufacturers’ instructions and the Infinite M Plex reader (Tecan, i-control version 3.9.1 software). Results were normalized to vehicle (DMSO) or to shNC. For cell-doubling assays, the cell number was quantified at the corresponding time points using Guava EasyCyte flow cytometer (Luminex, CytoSoft v5.2 software).

### Animal studies

All mice were housed and maintained under pathogen-free conditions in accordance with the institutional guidelines of the University Hospital Tübingen (12-h day–night cycle; temperature, 20–22 °C; humidity, 50–60%). Because CRC is a critical disease in male and female populations, both male and female mice were used. The sex was not considered in the study design.

Subcutaneous injection, splenic seeding or cecum injection of organoids was performed in 8–10-week-old C57BL/6 N/Crl, *Rag2*^*−/−*^ (B6.Cg-*Rag2*^*tm1.1Cgn*^/J), or CB17 Scid beige (CB17.Cg-Prkdc^scid^Lyst^bg-J^/Crl) mice, obtained from Charles River. The organoids were dissociated into single cells, washed with PBS and injected in a 1:1 mixture of PBS and Matrigel. For murine organoids, 1 × 10^5^ cells were injected into the left and right flanks or the spleen capsule and 2 × 10^6^ cells were injected into the cecum wall. For human organoids, 4 × 10^5^ cells were injected into the flanks of CB17 Scid beige mice. For splenic seeding and cecum injection, mice were anaesthetized using ketamine (100 mg kg^−1^, intraperitoneally) and xylazin (10 mg/kg, intraperitoneally) in NaCl and a small laparotomy below the thorax was performed. Upon injection, the puncture region was washed with prewarmed H_2_O. Within the first week after operation, the mice were treated with the analgesics carprofen and metamizol. The maximum size of individual tumor nodules permitted by the regional authority of the state of Baden-Wuerttemberg (Regierungspraesidium Tübingen: subcutaneous tumor nodules, 1 cm; other tumor nodules, 0.5 cm) was not exceeded.

In vivo treatment studies were performed in randomized groups with 20 mg kg^−1^ body weight of SKL, 1639 or 2015 and 100 mg kg^−1^ of navitoclax (Chemieliva Pharmaceutical, custom synthesis, lot 20210918) every day (one dose per day). The treatment start times are indicated in the figure legends. No mice were excluded during treatment studies. The drugs were administered through oral gavage dissolved in Phosal 50PG, ethanol and PEG400 (6:1:3) for 2015, 1639 and navitoclax or Cremophor, ethanol and H_2_O (1:1:6) for SKL. GFP imaging of tumors was performed using a Hamamatsu imaging system. For quantification of compounds in the murine plasma, 20 µl of tail-vein blood was diluted with 80 µl of heparin solution, before 200 µl of ice-cold acetonitrile with a defined internal standard was added. The samples were sonicated and centrifuged and the supernatant was subjected to liquid chromatography–mass spectrometry quantification. Full blood analyses were performed with the Sysmex KX-21N hematology analyzer.

### Histopathology and immunohistochemistry

Histopathological evaluation of colorectal tumors and healthy tissues was performed by experienced pathologists (S.S. for murine or human tumors and tissues; G.O. for human tumors). Hematoxylin and eosin (H&E) staining was performed on paraffin-embedded sections using 0.5% eosin and 0.5% hematoxylin.

Immunohistochemistry on tissue and organoid samples was performed on paraffin-embedded sections. Heat-induced antigen retrieval was performed with citrate buffer (10 mM C_6_H_8_O_7_ pH 6.0 and 0.05% Tween-20) for 20 min. All used antibodies and dilutions are listed in the Nature Portfolio Reporting Summary. For in vivo samples, a hematoxylin counterstaining was performed.

SA-β-Gal staining was carried out on sections of snap-frozen tumor tissues^56^. The samples were fixed with 0.5% glutaraldehyde solution (in PBS pH 7.4) for 15 min at room temperature and then washed once with PBS and twice with 1 mM MgCl_2_ in PBS pH 5.5 (5 min each, at room temperature). Subsequently, the samples were incubated with X-Gal solution (2.45 mM X-Gal, 5 mM K_3_Fe(CN)_6_, 5 mM K_4_Fe(CN)_6_ and 1 mM MgCl_2_ in PBS pH 5.5) at 37 °C overnight protected from light. Upon staining, the samples were washed three times with PBS (5 min each), fixed with 4% formalin in PBS for 30 min and again washed with PBS (5 min each, at room temperature).

Immunofluorescence staining on cells and organoids was performed on glass coverslips or tissue culture chambers (Sarstedt). Organoids were plated in medium containing 10% Matrigel or Cultrex BME. The cultures were fixed with 4% paraformaldehyde and stained with specific antibodies and DAPI (Vector Labs). All used antibodies and dilutions are listed in the Nature Portfolio Reporting Summary. For analyzing DNA synthesis, the Molecular Probes Click-iT EdU Alexa Fluor 555 kit was used 24 h after EdU incorporation with DAPI counterstaining. SA-β-Gal staining of cells was performed in cell culture dishes (cells) or tissue culture chambers (organoids) after fixation with 0.5% glutaraldehyde as described above. Human cultures were incubated with X-Gal solution at pH 6.0 for ~4 h.

Microscopic analyses were performed using the Olympus BX63 or the Olympus CKX53 microscopes (Olympus cellSens Dimension version 1.17, Olympus cellSens Standard version 3.2 or Olympus OlyVIA version 3.2.1 software). Positive cells were quantified using ImageJ version 1.53a software. The analysis of mitosis was performed with an LSM800 Airyscan (Zeiss) microscope (ZEN version 3.0 software). An Airyscan autofilter processing was applied before image analysis.

### Protein expression analyses

Proteins were extracted from cells and organoids using radioimmunoprecipitation assay extraction buffer (50 mM Tris pH 8.0, 150 mM NaCl, 1% Triton X-100, 0.5% sodium deoxycholate, 0.1% SDS, 5 mM EDTA and 1 mM EGTA) supplemented with protease and phosphatase inhibitors (cOmplete and PhosSTOP, Roche). Protein concentrations were measured using the DC (detergent compatible) Protein Assay (Bio-Rad). Briefly, 20–30 µg of protein was separated by SDS–PAGE and transferred onto PVDF membranes (Immobilon-P, Millipore). Membranes were blocked in 5% BSA and Tris-buffered saline (TBS)–Tween buffer and then incubated with primary antibodies overnight at 4 °C and with horseradish peroxidase-conjugated secondary antibodies for 1 h at room temperature. All used antibodies and dilutions are listed in the Nature Portfolio Reporting Summary. The blots were visualized using the ChemiDoc MP Imaging System (BIO-RAD) and ImageLab version 5.2.1 software. Vinculin and β-actin were used as loading controls.

### Flow cytometry

For DNA content analysis, cells were fixed with ethanol, washed and resuspended in PBS containing 50 µg ml^−1^ propidium iodide (Invitrogen) and 250 µg ml^−1^ RNase A (Qiagen). Apoptosis was analyzed with the FITC annexin V apoptosis detection kit (Biolegend). Both measurements were performed with FACS Canto II (BD) and physical parameters were used to exclude cell debris and cell doublets.

To determine immune cells, cell suspensions of peripheral blood, spleen, liver or tumors were prepared. Cells were lysed with ammonium chloride buffer (0.150 mM NH_4_Cl, 0.1 mM EDTA and 0.150 mM KHCO_3_) to eliminate erythrocytes and were stained with antibodies from Biolegend and the live–dead fixable aqua dead cell stain kit (Thermo Fisher Scientific; dilution: 1:1,000). All used antibodies and dilutions are listed in the Nature Portfolio Reporting Summary. Cells were analyzed with the LSR Fortessa (BD). Samples were gated on single viable cells and the percentage of NK cells (NK1.1^+^CD3^−^), T cells (CD3^+^NK1.1^−^), CD4^+^ T cells (CD3^+^NK1.1^−^CD4^+^CD8a^−^), CD8^+^ T cells (CD3^+^NK1.1^−^CD8a^+^CD4^−^), B cells (CD3^−^NK1.1^−^CD19^+^Ly6G^−^), dendritic cells (CD3^−^NK1.1^−^CD19^−^CD11c^+^), monocytes (CD3^−^NK1.1^−^CD19^−^CD11c^−^Ly6Ghigh^+^CD16/32low^+^) and granulocytes (CD3^−^NK1.1^−^CD19^−^CD11c^−^Ly6G^+^CD16/32high^+^) was calculated.

All FACS data were acquired with DIVA version 9.0.1 and analyzed with FlowJo version 10.8.0 software. The gating strategies are depicted in Extended Data Figs. 6c,d and 8a.

### Sequencing

For sequencing, genomic DNA was isolated using the AllPrep DNA + RNA Mini kit (Qiagen). The human CRC organoid cultures were analyzed by panel sequencing (target enrichment) using the tumor panel TUM01 library (CeGaT). The sequencing was performed using a NovaSeq 6000 device. SNVs, indels and selected CNVs were analyzed by CeGaT.

### Biochemical assays

The Wee1 kinase assay was performed with recombinant human active p38α protein (Abcam, ab271647), mostly inactive MK2 protein (Abcam, ab79910) and mostly inactive Wee1 protein (Novus Biologicals, H00007465-P01). Briefly, 200 ng of corresponding proteins were preincubated with 1 µM 2015 or DMSO in a kinase assay buffer (Abcam, ab189135; total volume, 100 µL) for 30 min at 37 °C. Kinase activity was induced with 20 µM ATP. Following a 30-min incubation at 37 °C, the reaction was stopped by protein inactivation (95 °C, 5 min in Laemmli buffer). The antibodies and dilutions for immunoblotting are listed in the Nature Portfolio Reporting Summary.

Potential interactions between 2015 and 340 wild-type kinases were checked by means of a competition binding assay performed by Reaction Biology Europe (Supplementary Table 15).

For evaluating p38α inhibition on isolated enzyme, an ELISA assay was performed^57^. First, 96-well plates were coated with 10 µg ml^−1^ ATF2 protein (Reaction biology, 0594-0000-2) in TBS (50 µl per well) and stored at 4 °C overnight. After washing with water, remaining free binding sites were blocked with blocking buffer (0.05% Tween-20, 0.025% BSA and 0.02% NaN_3_ in TBS) for 30 min at room temperature. Compounds were dissolved in DMSO (10 mM) and diluted (0.01–10 µM) in a kinase buffer containing 12 ng per 50 µl p38α protein (provided by J. Schultz, University of Tübingen), 50 mM Tris pH 7.5, 10 mM MgCl_2_, 10 mM β-glycerophosphate, 100 µg ml^−1^ BSA, 1 mM dithiothreitol, 0.1 mM Na_3_VO_4_ and 100 µM ATP. Then, 50 µl of each sample was pipetted into the corresponding wells and incubated for 1 h at 37 °C. The plates were washed three times, blocked for 15 min and washed again. Next, 50 µl of P-ATF2^T69/T71^ peroxidase conjugate antibody (Sigma A6228; clone: ATF-22P; lot: 123K4886; dilution: 1:5,000 in blocking buffer without NaN_3_, pH 6.5) was added into each well and incubated for 1 h at 37 °C. Finally, 50 µl of TMB substrate (BD Bioscience) was added to the wells. After 5 min, the reaction was stopped by adding 25 µl of 1 N HCl and the samples were measured photometrically at 450 nm with an ELISA reader (Molecular Devices, FilterMax F5) using SOFTmax PRO software (version 6.5.1).

TRTs were determined using a fluorescence polarization assay kit (Transcreener ADP, BellBrook Labs)^29^. The compounds were preincubated in 50 mM Tris pH 7.5, 1 mM dithiothreitol, 10 mM MgCl_2_, 10 mM β-glycerophosphate and 0.1 mM Na_3_VO_4_ at a concentration of 20-fold IC_50_ with p38α (12.4 µg ml^−1^) at room temperature for 1 h to enable the formation of an enzyme–inhibitor complex. The formed complex was ‘jump-diluted’ 1:100 (0.5 µl in 49.5 µl) in a 96-half-area-well plate with ATF2 (82 μM), ATP (40 μM) and ADP as the kinase assay detection reagents. The plate was measured every 3 min for 4 h. Fluorescence polarization was detected using the plate reader VictorNivo (PerkinElmer). The data were analyzed using the integrated rate equation in GraphPad Prism version 7.03 software. The residence time was calculated from the reciprocal value of *k*_off_.

### Molecular docking

The human p38α structure in complex with 1639 (Fig. 1f) (Protein Data Bank (PDB) 5TBE) was prepared using PrepWizard (Schrödinger Drug Discovery Tools, version 2022.4) and its missing loops were generated using Prime (version 2022.4). Ligands were drawn in Maestro (Schrödinger Drug Discovery Tools, version 2022.4) and prepared with LigPrep/Epik (Schrödinger Drug Discovery Tools, v2022.4) to assign the protonation state (at pH 7.0 ± 1.0). Molecular docking was performed using Glide version 9.7 (refs. ^58,59^) with the XP scoring function. For all software, standard options were used. Docking poses were visually inspected for the interaction with the hinge region and occupancy of the spine by relevant chemical moieties.

### Statistics and reproducibility

Statistical analyses were performed using a two-tailed Student’s *t*-test, one-way analysis of variance (ANOVA), Tukey’s or Dunnett’s multiple-comparisons tests or a log-rank test (for mouse survival analyses) as described in the figure legend for each experiment using GraphPad Prism version 9.41 software. Statistical significance for all experiments was considered at *P* < 0.05. Graphs were generated with GraphPad Prism version 7.03 or 9.41 software. Data analysis was performed using GraphPad Prism version 7.03 or 9.41 and Microsoft Office Professional Plus 2019 software. All measurements were taken from distinct samples. The indicated sample size (*n*) represents biological replicates. Drug–response curves and IC_50_ values were calculated using GraphPad Prism version 9.41 software. Survival of mice was measured using the Kaplan–Meier method. Data distribution was assumed to be normal but this was not formally tested. No statistical methods were used to predetermine sample sizes but our sample sizes are similar to those reported in previous publications^22,52,60,61^. Mice (of matched age), tumor samples and cultures of cell lines were randomly allocated to the respective groups of the experiments. No data and mice were excluded from the analyses. Data collection was performed blinded and group allocation was performed afterward. The collection of animal samples was not blinded because comparable samples were collected and no analysis was performed at this point. The analyses of samples were performed afterward (in follow-up experiments) in a blinded manner.

### Reporting summary

Further information on research design is available in the Nature Portfolio Reporting Summary linked to this article.

## Supplementary information

Reporting Summary

Supplementary Table Supplementary Tables 1–17.

## Source data

Source Data Unprocessed western blots and gels.

Source Data Fig. 1 Statistical source data.

Source Data Fig. 2 Statistical source data.

Source Data Fig. 3 Statistical source data.

Source Data Fig. 4 Statistical source data.

Source Data Fig. 5 Statistical source data.

Source Data Fig. 6 Statistical source data.

Source Data Fig. 7 Statistical source data.

Source Data Fig. 8 Statistical source data.

Source Data Extended Data Fig. 1 Statistical source data.

Source Data Extended Data Fig. 8 Statistical source data.

Source Data Extended Data Fig. 9 Statistical source data.

Source Data Extended Data Fig. 10 Statistical source data.

## Data Availability

Sequencing data of patient-derived CRC organoids (Extended Data Fig. 4c) were deposited to the Sequence Read Archive under accession code PRJNA955282. Processed sequencing data are provided in Supplementary Tables 4–13. A structural model of the p38α–1639 complex (Fig. 1f) was deposited to the Zenodo repository (10.5281/zenodo.7827886)^62^. The human p38α structure for molecular docking was obtained from the PDB under accession code 5TBE. Associated raw data of Figs. 2a,b and 3g are provided as Supplementary Tables. Anonymized data of participants, composition of organoid media and oligo sequences are also provided as Supplementary Tables. The gating strategy for flow cytometry is depicted in Extended Data Figs. 6c,d and 8a. All other data supporting the findings of this study are available from the corresponding author on reasonable request. Source data are provided with this paper.

## Code availability

No code was used in this study.
