# Supplementary material for: First-in-class ultralong-target-residence-time p38α inhibitors as a mitosis-targeted therapy for colorectal cancer
Source: Nat Cancer. 2025 Jan 16;6(2):259–77. doi: 10.1038/s43018-024-00899-7 (PMC11864979; doi:10.1038/s43018-024-00899-7)
Supplement: Supplementary file 1 — Reporting Summary [file 43018_2024_899_MOESM1_ESM.pdf]

nature portfolio

Corresponding author(s): Daniel Dauch

Last updated by author(s): Dec 10, 2024

Reporting Summary

Nature Portfolio wishes to improve the reproducibility of the work that we publish. This form provides structure for consistency and transparency in reporting. For further information on Nature Portfolio policies, see our Editorial Policies and the Editorial Policy Checklist.

Statistics

For all statistical analyses, confirm that the following items are present in the figure legend, table legend, main text, or Methods section.

n/a	Confirmed	
☐	☒	The exact sample size (*n*) for each experimental group/condition, given as a discrete number and unit of measurement
☐	☒	A statement on whether measurements were taken from distinct samples or whether the same sample was measured repeatedly
☐	☒	The statistical test(s) used AND whether they are one- or two-sided<br>*Only common tests should be described solely by name; describe more complex techniques in the Methods section.*
☒	☐	A description of all covariates tested
☒	☐	A description of any assumptions or corrections, such as tests of normality and adjustment for multiple comparisons
☐	☒	A full description of the statistical parameters including central tendency (e.g. means) or other basic estimates (e.g. regression coefficient) AND variation (e.g. standard deviation) or associated estimates of uncertainty (e.g. confidence intervals)
☐	☒	For null hypothesis testing, the test statistic (e.g. F, t, r) with confidence intervals, effect sizes, degrees of freedom and P value noted<br>*Give P values as exact values whenever suitable.*
☒	☐	For Bayesian analysis, information on the choice of priors and Markov chain Monte Carlo settings
☒	☐	For hierarchical and complex designs, identification of the appropriate level for tests and full reporting of outcomes
☒	☐	Estimates of effect sizes (e.g. Cohen's *d*, Pearson's *r*), indicating how they were calculated

Our web collection on statistics for biologists contains articles on many of the points above.

Software and code

Policy information about availability of computer code

Data collection	Olympus cellSens Dimension v1.17, Olympus cellSens Standard v3.2, Tecan i-control v3.9.1, SOFTmax PRO v6.5.1, ImageLab v5.2.1, DIVA v9.0.1, CytoSoft v5.2, ZEN v3.0
Data analysis	GraphPad Prism v7.03/v9.41, Microsoft Office Professional Plus 2019, ImageJ v1.53a, SnapGene Viewer v4.2.4, FlowJo v10.8.0, ImageLab v5.2.1, Olympus OlyVIA v3.2.1, PrepWizard tool (Schrödinger Drug Discovery tools, Maestro, LigPrep/Epik) v2022.4, Prime v2022.4, Glide v9.7

For manuscripts utilizing custom algorithms or software that are central to the research but not yet described in published literature, software must be made available to editors and reviewers. We strongly encourage code deposition in a community repository (e.g. GitHub). See the Nature Portfolio guidelines for submitting code & software for further information.

Data

Policy information about availability of data

All manuscripts must include a data availability statement. This statement should provide the following information, where applicable:

- Accession codes, unique identifiers, or web links for publicly available datasets
- A description of any restrictions on data availability
- For clinical datasets or third party data, please ensure that the statement adheres to our policy

Sequencing data of patient-derived CRC organoids (Extended Data Fig. 4c) have been deposited in the Sequence Read Archive (SRA) under accession code PRJNA955282. Processed sequencing data are provided in Supplementary Tables 4-13. Structural model of p38α-1639 complex (Fig. 1f) has been deposited in

Zenodo Repository under accession code 7827887. The human p38α structure for molecular docking was obtained from Protein Data Bank (PDB ID: 5TBE). Associated raw data of Figs. 2a, 2b and 3g are provided as Supplementary Tables. Anonymized data of patients, composition of organoid media and oligo sequences are also provided as Supplementary Tables. The gating strategy for flow cytometry is depicted in Extended Data Figs. 6c,d and 8a. Source data for Figs. 1,2,3,4,5,6,7,8 and Extended Data Figs. 1,3,4,5,8,9,10 have been provided as Source Data files. All other data supporting the findings of this study are available from the corresponding author on reasonable request.

Research involving human participants, their data, or biological material

Policy information about studies with human participants or human data. See also policy information about sex, gender (identity/presentation), and sexual orientation and race, ethnicity and racism.

Reporting on sex and gender	There were no restrictions regarding patient selection and no bias based on the sex and gender. Since colorectal cancer is a critical disease in male and female patients, organoid cultures from male and female patients were established and applied for the treatment studies. Patient-derived CRC organoids were established from CRC patients with the following sex: PDO1 = male, PDO2 = female, PDO3 = male, PDO4 = female, PDO5 = male, PDO6 = male, PDO7 = male, PDO8 = female, PDO9 = male, PDO10 = male, PDO11 = female, PDO12 = female, PDO13 = female, PDO14 = male, PDO15 = female.<br>The human colon organoids have the following sex: SCC311 = female, SCC321 = male.<br>Since the treatment responses of the cultures were analyzed individually, no sex- and gender-based analyses have been performed.
Reporting on race, ethnicity, or other socially relevant groupings	Not applicable. The study does not use the constructs of race and ethnicity.
Population characteristics	Patient-derived CRC organoids were established from adult CRC patients (both sexes) with the following age: PDO1 = 69 years, PDO2 = 70 years, PDO3 = 62 years, PDO4 = 51 years, PDO5 = 42 years, PDO6 = 58 years, PDO7 = 36 years, PDO8 = 36 years, PDO9 = 67 years, PDO10 = 52 years, PDO11 = 50 years, PDO12 = 70 years, PDO13 = 82 years, PDO14 = 69 years, PDO15 = 71 years.<br>The human colon organoids have the following age: SCC311 = 52 years, SCC321 = 21 years.<br>The treatment responses of the cultures were analyzed individually.
Recruitment	Human CRC organoids were isolated from tumor tissues or peritoneal fluid of adult patients from the University Hospital Tuebingen or the Robert Bosch Hospital Stuttgart. All patients gave their informed consent according to German law.
Ethics oversight	The experiments complied with all ethical regulations and were approved by the responsible ethics committee of the Medical Faculty of the University of Tuebingen and the University Hospital Tuebingen (ethic approval numbers 949/2021BO2, 117/2020BO1 and 696/2016BO2). All patients were deidentified and gave their written informed consent according to German law.

Note that full information on the approval of the study protocol must also be provided in the manuscript.

Field-specific reporting

Please select the one below that is the best fit for your research. If you are not sure, read the appropriate sections before making your selection.

☒ Life sciences ☐ Behavioural & social sciences ☐ Ecological, evolutionary & environmental sciences

For a reference copy of the document with all sections, see nature.com/documents/nr-reporting-summary-flat.pdf

Life sciences study design

All studies must disclose on these points even when the disclosure is negative.

Sample size	Samples sizes were estimated from similar experiments in the following former publications of the group to enable significant statistical analyses:<br>Rudalska R. et al. LXRα activation and Raf inhibition trigger lethal lipotoxicity in liver cancer. Nat Cancer. 2,201-217. doi: 10.1038/s43018-020-00168-3 (2021)<br>Rudalska, R. et al. In vivo RNAi screening identifies a mechanism of sorafenib resistance in liver cancer. Nat. Med 20, 1138-1146, doi:nm.3679 [pii];10.1038/nm.3679 [doi] (2014).<br>Dauch, D. et al. A MYC-aurora kinase A protein complex represents an actionable drug target in p53-altered liver cancer. Nat. Med 22, 744-753, doi:nm.4107 [pii];10.1038/nm.4107 [doi] (2016).<br>Kang, T. W. et al. Senescence surveillance of pre-malignant hepatocytes limits liver cancer development. Nature, doi:nature10599 [pii];10.1038/nature10599 [doi] (2011).)
Data exclusions	No data and mice were excluded from the analyses.
Replication	All performed replications (2 - 3 independent experiments) were successful.
Randomization	Mice of matched age, tumor samples and cell/organoid cultures were randomly allocated to the experiments.

Blinding

Collection of animal samples was not blinded since comparable samples were collected and no analysis was performed at this point. The analyses of samples were performed afterwards (in follow-up experiments) in a blinded manner.

Reporting for specific materials, systems and methods

We require information from authors about some types of materials, experimental systems and methods used in many studies. Here, indicate whether each material, system or method listed is relevant to your study. If you are not sure if a list item applies to your research, read the appropriate section before selecting a response.

Materials & experimental systems

n/a	Involved in the study
☐	☒ Antibodies
☐	☒ Eukaryotic cell lines
☒	☐ Palaeontology and archaeology
☐	☒ Animals and other organisms
☒	☐ Clinical data
☒	☐ Dual use research of concern
☒	☐ Plants

Methods

n/a	Involved in the study
☒	☐ ChIP-seq
☐	☒ Flow cytometry
☒	☐ MRI-based neuroimaging

Antibodies

Antibodies used

β-actin; Company: Cell Signaling Technology 3700; Clone: 8H10D10; LOT: 20; Dilution: 1:1000 (WB)
APC; Company: Novus Biologicals NB100-91662; LOT: D-4; Dilution: 1:1000 (WB)
ATF2; Company: Novus Biologicals NBP1-85457; LOT: A106709; Dilution: 1:50 (IHC) and 1:1000 (WB)
P-ATF2T71; Company: Cell Signaling Technology 9221; LOT: 9; Dilution: 1:50 (IHC)
P-ATF2T71; Company: Abcam ab32019; Clone: E268; LOT: GR3343271-5; Dilution: 1:1000 (WB)
P-Atf2T69/T71 Company: Sigma Aldrich A6228; Clone: ATF-22P; LOT: 123K4886; Dilution 1:5000 (ELISA)
Bcl-2; Company: Novus Biologicals NB100-56098; LOT: AR01A-092206E-0; Dilution: 1:1000 (WB)
BrafV600E; Company: Novus Biologicals NBP2-61503; Clone: RM8; LOT: U-11-04781; Dilution: 1:1000 (WB)
CD3-APC-Cy7; Company: Biolegend 100248; Clone: 17A2; LOT: B314610; Dilution: 1:80 (FACS)
CD4-APC; Company: Biolegend 100412; Clone: GK1.5; LOT: B340887; Dilution: 1:100 (FACS)
CD8a-FITC; Company: Biolegend 100706; Clone: 53-6.7; LOT: B347322; Dilution: 1:100 (FACS)
CD11c-BV650; Company: Biolegend 117339; Clone: N418; LOT: B376950; Dilution: 1:100 (FACS)
CD16/32-BV711; Company: Biolegend 101337; Clone: 93; LOT: B317054; Dilution: 1:250 (FACS)
CD19-PE-Dazzle; Company: Biolegend 115554; Clone: 6D5; LOT: B249535; Dilution: 1:100 (FACS)
CD69-BV421; Company: Biolegend 104545; Clone: H1.2F3; LOT: B398534; Dilution: 1:100 (FACS)
Cdc25B; Company: Cell Signaling Technology 9525; LOT: 2; Dilution: 1:1000 (WB)
Cdc25c; Company: MyBioSource MBS9602601; LOT: 39z5291; Dilution: 1:1000 (WB)
Cdk1; Company: Novus Biologicals NBP2-37626; Clone: 2G9; LOT: 121008; Dilution: 1:1000 (WB)
P-Cdk1/Cdc2Y15; Company: Cell Signaling Technology 4539; Clone: 10A11; LOT: 2; Dilution: 1:1000 (WB)
CDX2; Company: Abcam ab76541; Clone: EPR2764Y; LOT: GR300552-18; Dilution: 1:500 (IHC) and 1:500 (IF)
Chk1; Company: Novus Biologicals NB100-464; LOT: A2; Dilution: 1:1000 (WB)
Chk2; Company: BD Biosciences 611570; LOT: 32708; Dilution: 1:1000 (WB)
P-Chk2T68; Company: Abcam ab3501; LOT: GR302410-10; Dilution: 1:1000 (WB)
P-Chk1S317; Company: Cell Signaling Technology 12302; Clone: D12H3; LOT: 3; Dilution: 1:1000 (WB)
CK20; Company: Abcam ab97511; LOT: GR3398581-6; Dilution: 1:100 (IF)
Cleaved Caspase 3; Company: Cell Signaling Technology 9661; LOT: 47; Dilution: 1:200 (IHC)
Cyclin B1; Company: Cell Signaling Technology 4138; LOT: 3; Dilution: 1:1000 (WB)
pan-Cytokeratin; Company: Santa Cruz Biotechnology sc-81714; Clone: AE1/AE3; LOT: J1421; Dilution: 1:50 (IHC)
Elk1; Company: Cell Signaling Technology 9182; LOT: 5; Dilution: 1:50 (IHC) and 1:1000 (WB)
P-Elk1S383; Company: Abcam ab32799; LOT: GR159-4; Dilution: 1:200 (IHC)
P-Elk1S383; Company: Thermo Fisher Scientific MA5-15225; Clone: S.110.6; LOT: YK4130003; Dilution: 1:1000 (WB)
γH2A.XS139; Company: Cell Signaling Technology 9718; Clone: 20E3; LOT: 21; Dilution: 1:100 (IHC) and 1:1000 (WB)
P-Histone H3S10; Company: Abcam ab5176; LOT: GR11883-1; Dilution: 1:400 (IF)
HMGA2; Company: Abcam ab97276; LOT: GR256299-58; Dilution: 1:700 (IHC) and 1:1000 (WB)
HSP27; Company: Cell Signaling Technology 50353; Clone: E1J4D; LOT: 1; Dilution: 1:50 (IHC)
HSP27; Company: Cell Signaling Technology 2402; Clone: G31; LOT: 8; Dilution: 1:1000 (WB)
P-HSP27S82; Company: Cell Signaling Technology 2406; LOT: 3; Dilution: 1:50 (IHC)
P-HSP27S82; Company: Cell Signaling Technology 2401; LOT: 14; Dilution: 1:1000 (WB)
Ki67; Company: Abcam ab15580; LOT: GR3445756-1; Dilution: 1:450 (IHC)
Ly6G/Ly6C-PE; Company: Biolegend 108408; Clone: RB6-8C5; LOT: B220797; Dilution: 1:100 (FACS)
p38αMAPK; Company: Novus Biologicals NBP2-19662; LOT: 40100; Dilution: 1:250 (IHC)
p38αMAPK; Company: Cell Signaling Technology 9218; LOT: 9; Dilution: 1:1000 (WB)
P-p38αMAPKT180/Y182; Company: Abcam ab4822; LOT: 1008361-3; Dilution: 1:50 (IHC)
P-p38MAPKT180/Y182; Company: Cell Signaling Technology 4511; Clone: D3F9; LOT: 13; Dilution: 1:800 (IHC) and 1:1000 (WB)
MAPKAPK-2 (MK2); Company: Proteintech 13949-1-AP; LOT: 82438; Dilution: 1:100 (IHC)
MAPKAPK-2 (MK2); Company: Cell Signaling Technology 3042; LOT: 3; Dilution: 1:1000 (WB)
P-MAPKAPK-2T334; Company: Cell Signaling Technology 3007; Clone: 27B27; LOT: 12; Dilution: 1:100 (IHC)

P-MAPKAPK-2T334; Company: Cell Signaling Technology 3041, LOT: 6; Dilution: 1:1000 (WB)
MYC; Company: Abcam ab32072; Clone: Y69; LOT: GR3377350-15; Dilution: 1:5000 (WB)
NK1.1 BV785; Company: Biolegend 108749; Clone: PK136; LOT: B344098; Dilution: 1:166 (FACS)
p16INK4a; Company: Thermo Fisher Scientific MA5-17142; Clone: 1E12E10; LOT: WL3452077; Dilution: 1:1000 (WB)
p16Ink4a; Company: Santa Cruz Biotechnology sc-1207 (M-156); LOT: E0212; Dilution: 1:50 (IHC)
p53; Company: Novus Biologicals NBP2-62555; Clone: PAb421; LOT: T1928B22; Dilution: 1:1000 (WB)
α tubulin; Company: Novus Biologicals NB100-690; Clone: DM1A; LOT: G-3; Dilution: 1:1000 (IF)
Vinculin; Company: Sigma Aldrich V9131; Clone: hVIN-1; LOT: 079M4754V; Dilution: 1:5000 (WB)
Wee1; Company: Novus Biologicals NBP1-33506; LOT: 42837; Dilution: 1:1000 (WB for kinase assays)
Wee1; Company: Abcam ab203236; LOT: GR307738-1; Dilution: 1:1000 (WB)
P-Wee1S139; Company: Thermo Fisher Scientific PA5-105559; LOT: XH3676709; Dilution: 1:1000 (WB)
P-Wee1S642; Company: Cell Signaling Technology 4910; Clone: D47G5; LOT: 3; Dilution: 1:1000 (WB)

Validation

β-actin; Company: Cell Signaling Technology 3700; Clone: 8H10D10; LOT: 20. Application: Western Blotting in mouse cells. The antibody was validated by the manufacturer for the mentioned application in the mentioned species. Data are available on the manufacturer's website: (https://www.cellsignal.com/products/primary-antibodies/b-actin-8h10d10-mouse-mab/3700)
APC; Company: Novus Biologicals NB100-91662; LOT: D-4. Application: Western Blotting in mouse cells. The antibody was validated by the manufacturer for the mentioned application in the mentioned species. Data are available on the manufacturer's website: (https://www.novusbio.com/products/apc-antibody_nb100-91662)
ATF2; Company: Novus Biologicals NBP1-85457; LOT: A106709. Application: Immunohistochemistry in mouse tissue sections and Western Blotting in mouse cells. The antibody was validated by the manufacturer for the mentioned application in the mentioned species. Data are available on the manufacturer's website: https://www.novusbio.com/products/atf2-antibody_nbp1-85457
P-ATF2T71; Company: Cell Signaling Technology 9221; LOT: 9. Application: Immunohistochemistry in mouse tissue sections. The antibody was validated by the manufacturer for the mentioned application in the mentioned species. Data are available on the manufacturer's website: https://www.cellsignal.com/product/productDetail.jsp?productId=9221
P-ATF2T71; Company: Abcam ab32019; Clone: E268; LOT: GR3343271-5. Application: Western Blotting in mouse cells. The antibody was validated by the manufacturer for the mentioned application in the mentioned species. Data are available on the manufacturer's website: https://www.abcam.com/products/primary-antibodies/atf2-phospho-t71-antibody-e268-ab32019.html
P-Atf2T69/T71 Company: Sigma Aldrich A6228; Clone: ATF-22P; LOT: 123K4886. Application: ELISA for p38alpha. The antibody was validated in Goettert, M., Graeser, R. & Laufer, S.A. Optimization of a nonradioactive immunosorbent assay for p38alpha mitogen-activated protein kinase activity. Anal. Biochem 406, 233-234 (2010).
Bcl-2; Company: Novus Biologicals NB100-56098; LOT: AR01A-092206E-0. Application: Western Blotting in mouse cells. The antibody was validated by the manufacturer for the mentioned application in the mentioned species. Data are available on the manufacturer's website: (https://www.novusbio.com/products/bcl-2-antibody_nb100-56098)
BrafV600E; Company: Novus Biologicals NBP2-61503; Clone: RM8; LOT: U-11-04781. Application: Western Blotting in mouse cells with recombinant BRAFV600E expression. The antibody was validated by the manufacturer for the mentioned application in the mentioned species. Data are available on the manufacturer's website: (https://www.novusbio.com/products/b-raf-antibody-rm8_nbp2-61503)
CD3-APC-Cy7; Company: Biolegend 100248; Clone: 17A2; LOT: B314610. Application: FACS analysis in mouse cells. The antibody was validated by the manufacturer for the mentioned application in the mentioned species. Data are available on the manufacturer's website: https://www.biolegend.com/en-us/products/apc-fire-750-anti-mouse-cd3-antibody-13052
CD4-APC; Company: Biolegend 100412; Clone: GK1.5; LOT: B340887. Application: FACS analysis in mouse cells. The antibody was validated by the manufacturer for the mentioned application in the mentioned species. Data are available on the manufacturer's website: https://www.biolegend.com/en-us/products/apc-anti-mouse-cd4-antibody-245
CD8a-FITC; Company: Biolegend 100706; Clone: 53-6.7; LOT: B347322. Application: FACS analysis in mouse cells. The antibody was validated by the manufacturer for the mentioned application in the mentioned species. Data are available on the manufacturer's website: https://www.biolegend.com/en-us/products/fitc-anti-mouse-cd8a-antibody-153
CD11c-BV650; Company: Biolegend 117339; Clone: N418; LOT: B376950. Application: FACS analysis in mouse cells. The antibody was validated by the manufacturer for the mentioned application in the mentioned species. Data are available on the manufacturer's website: https://www.biolegend.com/en-us/products/brilliant-violet-650-anti-mouse-cd11c-antibody-8840
CD16/32-BV711; Company: Biolegend 101337; Clone: 93; LOT: B317054. Application: FACS analysis in mouse cells. The antibody was validated by the manufacturer for the mentioned application in the mentioned species. Data are available on the manufacturer's website: https://www.biolegend.com/en-us/products/brilliant-violet-711-anti-mouse-cd16-32-11835
CD19-PE-Dazzle; Company: Biolegend 115554; Clone: 6D5; LOT: B249535. Application: FACS analysis in mouse cells. The antibody was validated by the manufacturer for the mentioned application in the mentioned species. Data are available on the manufacturer's website: https://www.biolegend.com/en-us/products/pe-dazzle-594-anti-mouse-cd19-antibody-10071
CD69-BV421; Company: Biolegend 104545; Clone: H1.2F3; LOT: B398534. Application: FACS analysis in mouse cells. The antibody was validated by the manufacturer for the mentioned application in the mentioned species. Data are available on the manufacturer's website: https://www.biolegend.com/en-us/products/brilliant-violet-421-anti-mouse-cd69-antibody-7358
Cdc25B; Company: Cell Signaling Technology 9525; LOT: 2. Application: Western Blotting in mouse cells. The antibody was validated by the manufacturer for the mentioned application in the mentioned species. Data are available on the manufacturer's website: (https://www.cellsignal.com/products/primary-antibodies/cdc25b-antibody/9525)
Cdc25c; Company: MyBioSource MBS9602601; LOT: 39z5291. Application: Western Blotting in mouse cells. The antibody was validated by the manufacturer for the mentioned application in the mentioned species. Data are available on the manufacturer's website: (https://biocheminfo.org/mbs9602601-cdc25c-antibody/)
Cdk1; Company: Novus Biologicals NBP2-37626; Clone: 2G9; LOT: 121008. Application: Western Blotting in mouse cells. The antibody was validated by the manufacturer for the mentioned application in the mentioned species. Data are available on the manufacturer's website: (https://www.novusbio.com/products/cdc2-cdk1-antibody-2g9_nbp2-37626)
P-Cdk1/Cdc2Y15; Company: Cell Signaling Technology 4539; Clone: 10A11; LOT: 2. Application: Western Blotting in mouse cells. The antibody was validated by the manufacturer for the mentioned application in the mentioned species. Data are available on the manufacturer's website: (https://www.cellsignal.com/products/primary-antibodies/phospho-cdc2-tyr15-10a11-rabbit-mab/4539)
CDX2; Company: Abcam ab76541; Clone: EPR2764Y; LOT: GR300552-18. Application: Immunohistochemistry in mouse and human tissue sections and immunofluorescence in mouse and human cells. The antibody was validated by the manufacturer for the mentioned application in the mentioned species. Data are available on the manufacturer's website: (https://www.abcam.com/products/primary-antibodies/cdx2-antibody-epr2764y-ab76541.html)

Chk1; Company: Novus Biologicals NB100-464; LOT: A2. Application: Western Blotting in mouse cells. The antibody was validated by the manufacturer for the mentioned application in the mentioned species. Data are available on the manufacturer's website: (https://www.novusbio.com/products/chk1-antibody_nb100-464)
Chk2; Company: BD Biosciences 611570; LOT: 32708. Application: Western Blotting in mouse cells. The antibody was validated by the manufacturer for the mentioned application in the mentioned species. Data are available on the manufacturer's website: https://www.bdbiosciences.com/en-de/products/reagents/microscopy-imaging-reagents/immunofluorescence-reagents/purified-mouse-anti-chk2.611570
P-Chk2T68; Company: Abcam ab3501; LOT: GR302410-10. Application: Western Blotting in mouse cells. The antibody was validated by the manufacturer for the mentioned application in the mentioned species. Data are available on the manufacturer's website: https://www.abcam.com/products/primary-antibodies/chk2-phospho-t68-antibody-ab3501.html
P-Chk1S317; Company: Cell Signaling Technology 12302; Clone: D12H3; LOT: 3. Application: Western Blotting in mouse cells. The antibody was validated by the manufacturer for the mentioned application in the mentioned species. Data are available on the manufacturer's website: https://www.cellsignal.com/products/primary-antibodies/phospho-chk1-ser317-d12h3-xp-rabbit-mab/12302
CK20; Company: Abcam ab97511; LOT: GR3398581-6. Application: Immunofluorescence in mouse and human cells. The antibody was validated by the manufacturer for the mentioned application in the mentioned species. Data are available on the manufacturer's website: https://www.abcam.com/products/primary-antibodies/cytokeratin-20-antibody-cytoskeleton-marker-ab97511.html
Cleaved Caspase 3; Company: Cell Signaling Technology 9661; LOT: 47. Application: Immunohistochemistry in mouse tissue sections. The antibody was validated by the manufacturer for the mentioned application in the mentioned species. Data are available on the manufacturer's website: https://www.cellsignal.com/products/primary-antibodies/cleaved-caspase-3-asp175-antibody/9661
Cyclin B1; Company: Cell Signaling Technology 4138; LOT: 3. Application: Western Blotting in mouse cells. The antibody was validated by the manufacturer for the mentioned application in the mentioned species. Data are available on the manufacturer's website: https://www.cellsignal.com/products/primary-antibodies/cyclin-b1-antibody/4138
Elk1; Company: Cell Signaling Technology 9182; LOT: 5. Application: Immunohistochemistry in mouse tissue sections and Western Blotting in mouse cells. The antibody was validated by the manufacturer for the mentioned application in the mentioned species. Data are available on the manufacturer's website: https://www.cellsignal.com/products/primary-antibodies/elk-1-antibody/9182
P-Elk1S383; Company: Abcam ab32799; LOT: GR159-4. Application: Immunohistochemistry in mouse tissue sections. The antibody was validated by the manufacturer for the mentioned application in the mentioned species. Data are available on the manufacturer's website: https://www.abcam.com/products/primary-antibodies/elk1-phospho-s383-antibody-chip-grade-ab32799.html
P-Elk1S383; Company: Thermo Fisher Scientific MA5-15225; Clone: S.110.6; LOT: YK4130003. Application: Western Blotting in mouse cells. The antibody was validated by the manufacturer for the mentioned application in the mentioned species. Data are available on the manufacturer's website: https://www.thermofisher.com/antibody/product/Phospho-ELK1-Ser383-Antibody-clone-S-110-6-Monoclonal/MA5-15225
γH2A.XS139; Company: Cell Signaling Technology 9718; Clone: 20E3; LOT: 21. Application: Immunohistochemistry in mouse tissue sections and Western Blotting in mouse cells. The antibody was validated by the manufacturer for the mentioned application in the mentioned species. Data are available on the manufacturer's website: https://www.cellsignal.com/products/primary-antibodies/phospho-histone-h2a-x-ser139-20e3-rabbit-mab/9718
P-Histone H3S10; Company: Abcam ab5176; LOT: GR11883-1. Application: Immunofluorescence in mouse and human cells. The antibody was validated by the manufacturer for the mentioned application in the mentioned species. Data are available on the manufacturer's website: https://www.abcam.com/products/primary-antibodies/histone-h3-phospho-s10-antibody-ab5176.html
HMGA2; Company: Abcam ab97276; LOT: GR256299-58. Application: Immunohistochemistry in mouse tissue sections and Western Blotting in mouse cells. The antibody was validated by the manufacturer for the mentioned application in the mentioned species. Data are available on the manufacturer's website: https://www.abcam.com/products/primary-antibodies/hmga2-antibody-ab97276.html
HSP27; Company: Cell Signaling Technology 50353; Clone: E1J4D; LOT: 1. Application: Immunohistochemistry in mouse tissue sections. The antibody was validated by the manufacturer for the mentioned application in the mentioned species. Data are available on the manufacturer's website: https://www.cellsignal.com/products/primary-antibodies/hsp27-e1j4d-rabbit-mab/50353
HSP27; Company: Cell Signaling Technology 2402; Clone: G31; LOT: 8. Application: Western Blotting in mouse cells. The antibody was validated by the manufacturer for the mentioned application in the mentioned species. Data are available on the manufacturer's website: https://www.cellsignal.com/products/primary-antibodies/hsp27-g31-mouse-mab/2402
P-HSP27S82; Company: Cell Signaling Technology 2401; LOT: 14. Application: Western Blotting in mouse cells. The antibody was validated by the manufacturer for the mentioned application in the mentioned species. Data are available on the manufacturer's website: https://www.cellsignal.com/products/primary-antibodies/phospho-hsp27-ser82-antibody/2401
P-HSP27S82; Company: Cell Signaling Technology 2406; LOT: 3. Application: Immunohistochemistry in mouse tissue sections. The antibody was validated by the manufacturer for the mentioned application in the mentioned species. Data are available on the manufacturer's website: https://www.cellsignal.com/products/primary-antibodies/phospho-hsp27-ser82-antibody-ii/2406
Ki67; Company: Abcam ab15580; LOT: GR3445756-1. Application: Immunohistochemistry in mouse tissue sections. The antibody was validated by the manufacturer for the mentioned application in the mentioned species. Data are available on the manufacturer's website: https://www.abcam.com/products/primary-antibodies/ki67-antibody-ab15580.html
Ly6G/Ly6C-PE; Company: Biolegend 108408; Clone: RB6-8C5; LOT: B220797. Application: FACS analysis in mouse cells. The antibody was validated by the manufacturer for the mentioned application in the mentioned species. Data are available on the manufacturer's website: https://www.biolegend.com/en-us/products/pe-anti-mouse-ly-6g-ly-6c-gr-1-antibody-460
p38αMAPK; Company: Novus Biologicals NBP2-19662; LOT: 40100. Application: Immunohistochemistry in mouse and human tissue sections. The antibody was validated by the manufacturer for the mentioned application in the mentioned species. Data are available on the manufacturer's website: https://www.novusbio.com/products/p38-alpha-antibody_nbp2-19662
p38αMAPK; Company: Cell Signaling Technology 9218; LOT: 9. Application: Western Blotting in mouse cells and in assays with recombinant human protein. The antibody was validated by the manufacturer for the mentioned application in the mentioned species. Data are available on the manufacturer's website: https://www.cellsignal.com/products/primary-antibodies/p38a-mapk-antibody/9218
P-p38αMAPKT180/Y182; Company: Abcam ab4822; LOT: 1008361-3. Application: Immunohistochemistry in mouse tissue sections. The antibody was validated by the manufacturer for the mentioned application in the mentioned species. Data are available on the manufacturer's website: https://www.abcam.com/products/primary-antibodies/p38-phospho-t180--y182-antibody-ab4822.html
P-p38MAPKT180/Y182; Company: Cell Signaling Technology 4511; Clone: D3F9; LOT: 13. Application: Immunohistochemistry in human tissue sections and Western Blotting in mouse cells and in assays with recombinant human protein. The antibody was validated by the manufacturer for the mentioned application in the mentioned species. Data are available on the manufacturer's website: https://www.cellsignal.com/products/primary-antibodies/phospho-p38-mapk-thr180-tyr182-d3f9-xp-rabbit-mab/4511

MAPKAPK-2 (MK2); Company: Proteintech 13949-1-AP; LOT: 82438. Application: Immunohistochemistry in mouse tissue sections. The antibody was validated by the manufacturer for the mentioned application in the mentioned species. Data are available on the manufacturer's website: https://www.ptglab.com/products/MAPKAPK2-Antibody-13949-1-AP.htm
MAPKAPK-2 (MK2); Company: Cell Signaling Technology 3042; LOT: 3. Application: Western Blotting in mouse cells and in assays with recombinant human protein. The antibody was validated by the manufacturer for the mentioned application in the mentioned species. Data are available on the manufacturer's website: https://www.cellsignal.com/products/primary-antibodies/mapkapk-2-antibody/3042
P-MAPKAPK-2T334; Company: Cell Signaling Technology 3007; Clone: 27B27; LOT: 12. Application: Immunohistochemistry in mouse tissue sections. The antibody was validated by the manufacturer for the mentioned application in the mentioned species. Data are available on the manufacturer's website: https://www.cellsignal.com/products/primary-antibodies/phospho-mapkapk-2-thr334-27b7-rabbit-mab/3007
P-MAPKAPK-2T334; Company: Cell Signaling Technology 3041, LOT: 6. Application: Western Blotting in mouse cells and in assays with recombinant human protein. The antibody was validated by the manufacturer for the mentioned application in the mentioned species. Data are available on the manufacturer's website: https://www.cellsignal.com/product/productDetail.jsp?productId=3041
MYC; Company: Abcam ab32072; Clone: Y69; LOT: GR3377350-15. Application: Western Blotting in mouse cells. The antibody was validated by the manufacturer for the mentioned application in the mentioned species. Data are available on the manufacturer's website: https://www.abcam.com/products/primary-antibodies/c-myc-antibody-y69-chip-grade-ab32072.html
NK1.1 BV785; Company: Biolegend 108749; Clone: PK136; LOT: B344098. Application: FACS analysis in mouse cells. The antibody was validated by the manufacturer for the mentioned application in the mentioned species. Data are available on the manufacturer's website: https://www.biolegend.com/en-us/products/brilliant-violet-785-anti-mouse-nk-1-1-antibody-10367
pan-Cytokeratin; Company: Santa Cruz Biotechnology sc-81714; Clone: AE1/AE3; LOT: J1421. Application: Immunohistochemistry in mouse and human tissue sections. The antibody was validated by the manufacturer for the mentioned application in the mentioned species. Data are available on the manufacturer's website: https://www.scbt.com/p/pan-cytokeratin-antibody-ae1-ae3?requestFrom=search
p16INK4a; Company: Thermo Fisher Scientific MA5-17142; Clone: 1E12E10; LOT: WL3452077. Application: Western Blotting in mouse cells. The antibody was validated by the manufacturer for the mentioned application in the mentioned species. Data are available on the manufacturer's website: https://www.thermofisher.com/antibody/product/MA5-17142.html?gclid=EAIaIQobChMIn-u2gZWV_gIVGOh3Ch2MhwqKEAAYASAAEgIxRfD_BwE&ef_id=EAIaIQobChMIn-u2gZWV_gIVGOh3Ch2MhwqKEAAYASAAEgIxRfD_BwE:G:s&s_kwcid=AL!3652!3!459737518508!!!g!!!10950825775!106531320406&cid=bid_pca_aup_r01_co_cp1359_pjt0000_bid00000_0se_gaw_dy_pur_con
p16Ink4a; Company: Santa Cruz Biotechnology sc-1207 (M-156); LOT: E0212. Application: Immunohistochemistry in mouse tissue sections. The antibody was validated by the manufacturer for the mentioned application in the mentioned species. Data are available on the website: https://www.citeab.com/antibodies/809812-sc-1207-p16-m-156
p53; Company: Novus Biologicals NBP2-62555; Clone: PAb421; LOT: T1928B22. Application: Western Blotting in mouse cells. The antibody was validated by the manufacturer for the mentioned application in the mentioned species. Data are available on the manufacturer's website: https://www.novusbio.com/products/p53-antibody-pab421_nbp2-62555
α tubulin; Company: Novus Biologicals NB100-690; Clone: DM1A; LOT: G-3. Application: Immunofluorescence in mouse and human cells. The antibody was validated by the manufacturer for the mentioned application in the mentioned species. Data are available on the manufacturer's website: https://www.novusbio.com/products/alpha-tubulin-antibody-dm1a_nb100-690
Vinculin; Company: Sigma Aldrich V9131; Clone: hVIN-1; LOT: 079M4754V. Application: Western Blotting in mouse cells. The antibody was validated by the manufacturer for the mentioned application in the mentioned species. Data are available on the manufacturer's website: https://www.sigmaaldrich.com/DE/de/product/sigma/v9131
Wee1; Company: Novus Biologicals NBP1-33506; LOT: 42837. Application: Western Blotting in assays with recombinant human protein. The antibody was validated by the manufacturer for the mentioned application in the mentioned species. Data are available on the manufacturer's website: https://www.novusbio.com/products/wee1-antibody_nbp1-33506
Wee1; Company: Abcam ab203236; LOT: GR307738-1. Application: Western Blotting in mouse cells. The antibody was validated by the manufacturer for the mentioned application in the mentioned species. Data are available on the manufacturer's website: https://www.abcam.com/products/primary-antibodies/wee1-antibody-ab203236.html
P-Wee1S139; Company: Thermo Fisher Scientific PA5-105559; LOT: XH3676709. Application: Western Blotting in mouse cells. The antibody was validated by the manufacturer for the mentioned application in the mentioned species. Data are available on the manufacturer's website: https://www.thermofisher.com/antibody/product/Phospho-WEE1-Ser139-Antibody-Polyclonal/PA5-105559
P-Wee1S642; Company: Cell Signaling Technology 4910; Clone: D47G5; LOT: 3. Application: Western Blotting in mouse cells and in assays with recombinant human protein. The antibody was validated by the manufacturer for the mentioned application in the mentioned species. Data are available on the manufacturer's website: https://www.cellsignal.com/products/primary-antibodies/phospho-wee1-ser642-d47g5-rabbit-mab/4910

Eukaryotic cell lines

Policy information about cell lines and Sex and Gender in Research

Cell line source(s)

NT-I organoids were isolated from a female B6.129-Krastm4Tyj Trp53tm1Brn/J (LSL-KrasG12D+/- x Trp53fl/fl) mouse.
NT-II organoids were isolated from a female C57BL/6-Apctm1Tyj/J (Apcfl/fl) mouse.
KAP and KMP organoids are derived from NT-I organoids.
BAP organoids are derived from NT-II organoids.
KAP2D cells are derived from KAP organoids.
Human CRC organoids were isolated from tumor tissues or peritoneal fluid of adult patients from the University Hospital Tuebingen or the Robert Bosch Hospital Stuttgart (PDO1 = male, PDO2 = female, PDO3 = male, PDO4 = female, PDO5 = male, PDO6 = male, PDO7 = male, PDO8 = female, PDO9 = male, PDO10 = male, PDO11 = female, PDO12 = female, PDO13 = female, PDO14 = male, PDO15 = female).
Phoenix-Eco packaging cells (CRL-3214), HCT-15 (CCL-225), HCT 116 (CCL-247), COLO 205 (CCL-222), LS 174T (CL-188), RKO (CRL-2577) and HT-29 (HTB-38) were obtained from the American Type Culture Collection.
The human colon organoids SCC311, SCC321 were obtained from Sigma-Aldrich.

Authentication

Murine organoid cultures and the KAP2D cell line were authenticated by PCR (Kras locus, Trp53locus, Myc transposon,

Authentication	BrafV600E cDNA). Phoenix-Eco packaging cells, HCT-15, HCT 116, COLO 205, LS 174T, RKO and HT-29 cells were authenticated by the provider (STR profiling).
Mycoplasma contamination	Cell lines and organoid cultures were tested negative for mycoplasma contamination.
Commonly misidentified lines (See ICLAC register)	No commonly misidentified cell lines were used.

Animals and other research organisms

Policy information about studies involving animals; ARRIVE guidelines recommended for reporting animal research, and Sex and Gender in Research

Laboratory animals	Murine colon organoids were isolated from 10 weeks old female B6.129-Krastm4Tyj Trp53tm1Brn/J (LSL-KrasG12D+/- x Trp53fl/fl) and C57BL/6-Apctm1Tyj/J (Apcfl/fl) mice.<br>Subcutaneous injections, splenic seeding or caecum injections of CRC organoids were performed in 8 to 10 weeks old male or female C57BL/6N/Crl WT mice, Rag2-/- mice (B6.Cg-Rag2tm1.1Cgn/J) or CB17 Scid beige mice (CB17.Cg-PrkdcscidLystbg-J/Crl) (obtained from Charles River).<br>All mice were housed and maintained under pathogen free conditions in accordance with the institutional guidelines of the University Hospital Tuebingen (day/night cycle: 12h, temperature: 20-22 °C, humidity: 50 - 60%).
Wild animals	The study did not involve wild animals.
Reporting on sex	The findings in our study do not apply to only one sex. Since colorectal cancer is a critical disease in male and female patients, mice of both sexes were used in the study. The sex was not considered in the study design.
Field-collected samples	The study did not involve field-collected samples.
Ethics oversight	All animal experiments in this study complied with all relevant ethical regulations and were approved by committees of the regional authority of the state of Baden-Wuerttemberg (Regierungspraesidium Tuebingen, authorization number: M17/18G).<br>The maximum size of individual tumor nodules, permitted by the regional authority of the state of Baden-Wuerttemberg (Regierungspraesidium Tuebingen, subcutaneous tumor nodules = 1 cm/other tumor nodules = 0.5 cm) was not exceeded.

Note that full information on the approval of the study protocol must also be provided in the manuscript.

Flow Cytometry

Plots

Confirm that:

☒ The axis labels state the marker and fluorochrome used (e.g. CD4-FITC).

☒ The axis scales are clearly visible. Include numbers along axes only for bottom left plot of group (a 'group' is an analysis of identical markers).

☒ All plots are contour plots with outliers or pseudocolor plots.

☒ A numerical value for number of cells or percentage (with statistics) is provided.

Methodology

Sample preparation	For flow cytometric analysis of DNA content, KAP2D cells were trypsinized, washed, fixed with ethanol and left overnight at 4° C. The fixed cells were washed and resuspended in PBS containing 50 µg/ml propidium iodide (Invitrogen) and 250 µg/ml RNase A (Qiagen).<br>To analyze apoptosis, KAP2D cells were trypsinized and stained with Annexin V using the FITC Annexin V Apoptosis Detection Kit (Biolegend).<br>To determine immune cells, cell suspensions of blood, spleen, liver or liver metastases were prepared and peripheral blood was collected. Cells were lysed with ammonium chloride buffer (0.150 mM NH4Cl, 0.1 mM EDTA, 0.150 mM KHCO3) to eliminate erythrocytes. The cells were stained with CD3-APC-Cy7 (Biolegend 100248; Clone: 17A2; LOT: B314610; Dilution: 1:80), CD4-APC (Biolegend 100412; Clone: GK1.5; LOT: B340887; Dilution: 1:100), CD8a-FITC (Biolegend 100706; Clone: 53-6.7; LOT: B347322; Dilution: 1:100), CD11c-BV650 (Biolegend 117339; Clone: N418; LOT: B376950; Dilution: 1:100), CD16/32-BV711 (Biolegend 101337; Clone: 93; LOT: B317054; Dilution: 1:250), CD19-PE-Dazzle (Biolegend 115554; Clone: 6D5; LOT: B249535; Dilution: 1:100), CD69-BV421 (Biolegend 104545; Clone: H1.2F3; LOT: B398534; Dilution: 1:100), Ly6G/Ly6C-PE (Biolegend 108408; Clone: RB6-8C5; LOT: B220797; Dilution: 1:100), NK1.1 BV785 (Biolegend 108749; Clone: PK136; LOT: B344098; Dilution: 1:166), and LIVE/DEAD™ Fixable Aqua Dead Cell Stain Kit (Thermo Fisher, 1:1.000).
Instrument	FACSCanto flow cytometer (BD) (DNA content staining, Annexin V staining), LSR Fortessa flow cytometer (BD) (Immune cell analyses).
Software	FlowJo v10.8.0, DIVA v9.0.1.
Cell population abundance	DNA content staining (Fig. 4m): Cell population abundance = 40-63%, dependent on the treatment (as shown in Extended Data Fig. 6c).<br>Annexin V staining (Fig. 5a): Cell population abundance = 41-92%, dependent on the treatment (as shown in Extended Data

Fig. 6d).
Immune cell analyses (Fig. 6f,g, Extended Data Fig. 8b): Cell population abundance of single, viable cells ~80%. Further details are shown in Extended Data Fig. 8a.

Gating strategy	DNA content staining (Fig. 4m): Physical parameters were used to exclude cell debris and cell doublets as shown in Extended Data Fig. 6c.<br>Annexin V staining (Fig. 5a): Physical parameters were used to exclude cell debris as shown in Extended Data Fig. 6d.<br>Immune cell analyses (Fig. 6f,g, Extended Data Fig. 8b): Data samples were gated on single, viable cells. Within this population percentage of NK cells (NK1.1+CD3-), T cells (CD3+NK1.1-), CD4+ T cells (CD3+NK1.1-/CD4+ CD8a-), CD8+ T cells (CD3+NK1.1-/CD8a+CD4-), B cells (CD3-NK1.1-/CD19+Ly6G-), DCs (CD3-NK1.1-/CD19-/CD11c+), Monocytes (CD3-NK1.1-/CD19-/CD11c-/Ly6Ghigh+CD16/32low+), Granulocytes (CD3-NK1.1-/CD19-/CD11c-/Ly6G+CD16/32high+) was calculated (as shown in Extended Data Fig. 8a.

☒ Tick this box to confirm that a figure exemplifying the gating strategy is provided in the Supplementary Information.
