# Supplementary material for: First-in-class ultralong-target-residence-time p38α inhibitors as a mitosis-targeted therapy for colorectal cancer
Source: Nat Cancer. 2025 Jan 16;6(2):259–77. doi: 10.1038/s43018-024-00899-7 (PMC11864979; doi:10.1038/s43018-024-00899-7)

Figure 1b:

p38α

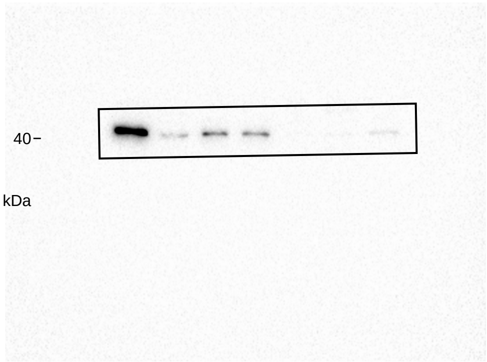

Vinculin

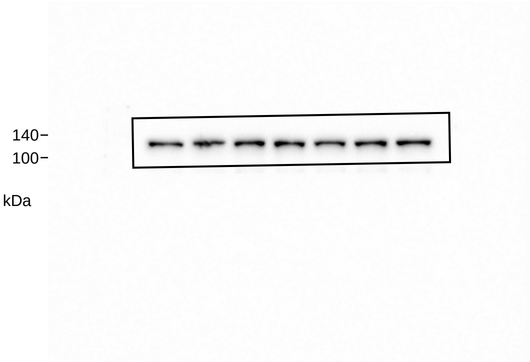

Figure 1e:

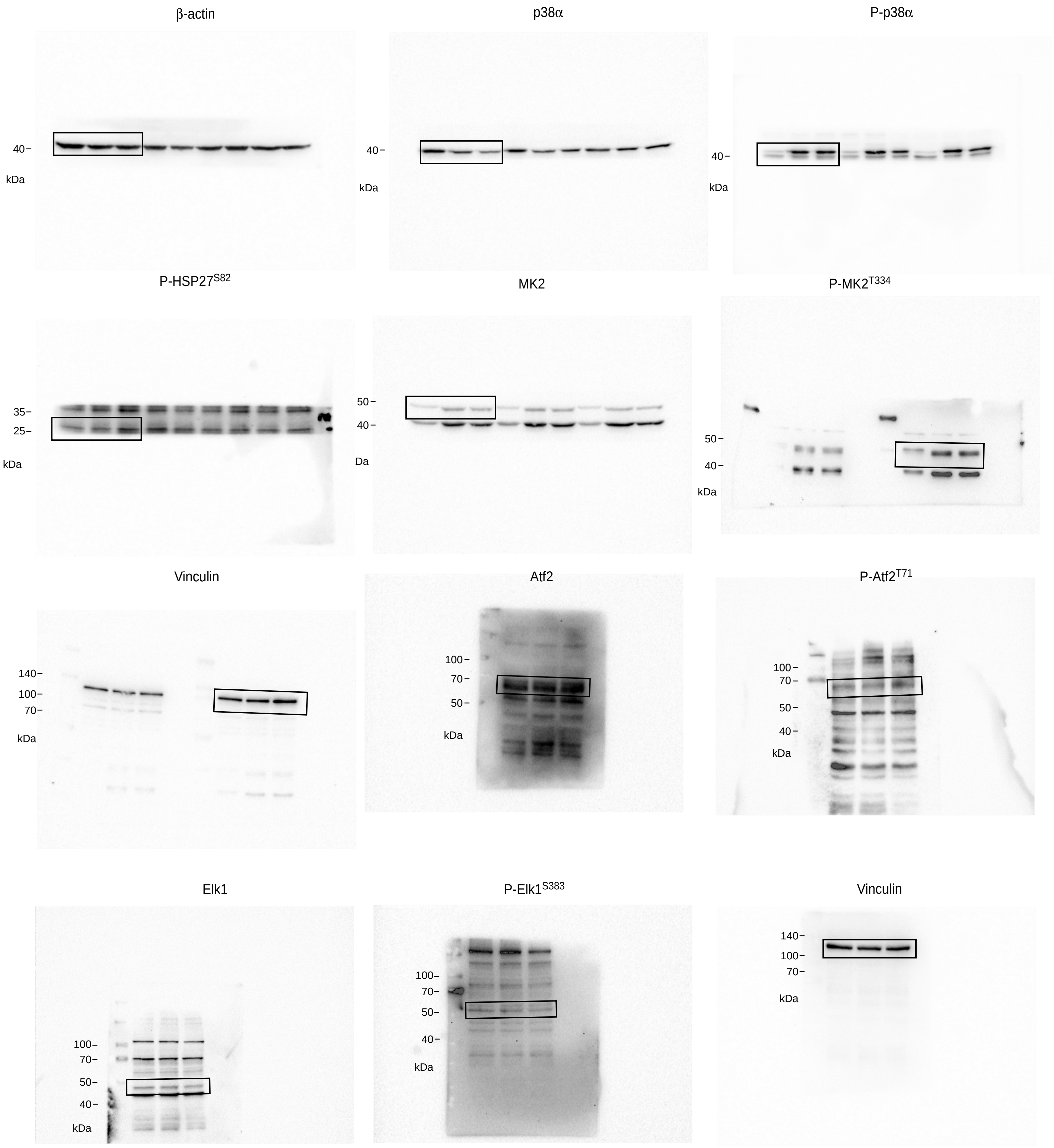
β-actin
40–
kDa
p38α
40–
kDa
P-p38α
40–
kDa
P-HSP27^{S82}
35–
25–
kDa
MK2
50–
40–
Da
P-MK2^{T334}
50–
40–
kDa
Vinculin
140–
100–
70–
kDa
Atf2
100–
70–
50–
kDa
P-Atf2^{T71}
100–
70–
50–
40–
kDa
Elk1
100–
70–
50–
40–
kDa
P-Elk1^{S383}
100–
70–
50–
40–
kDa
Vinculin
140–
100–
70–
kDa

Figure 4a:

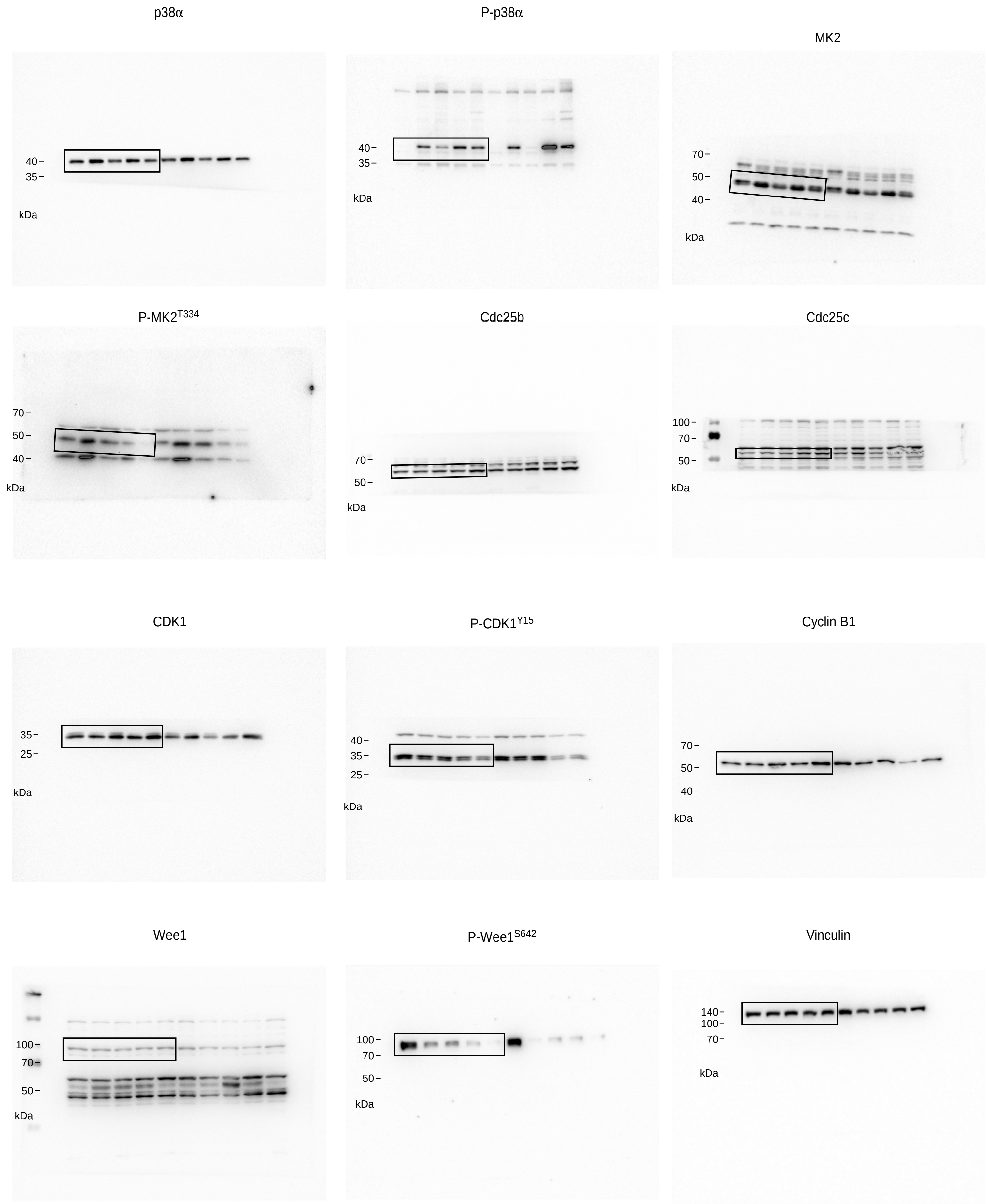
p38α
40
35
kDa
P-p38α
40
35
kDa
MK2
70
50
40
kDa
P-MK2T334
70
50
40
kDa
Cdc25b
70
50
kDa
Cdc25c
100
70
50
kDa
CDK1
35
25
kDa
P-CDK1Y15
40
35
25
kDa
Cyclin B1
70
50
40
kDa
Wee1
100
70
50
kDa
P-Wee1S642
100
70
50
kDa
Vinculin
140
100
70
kDa

Figure 4b:

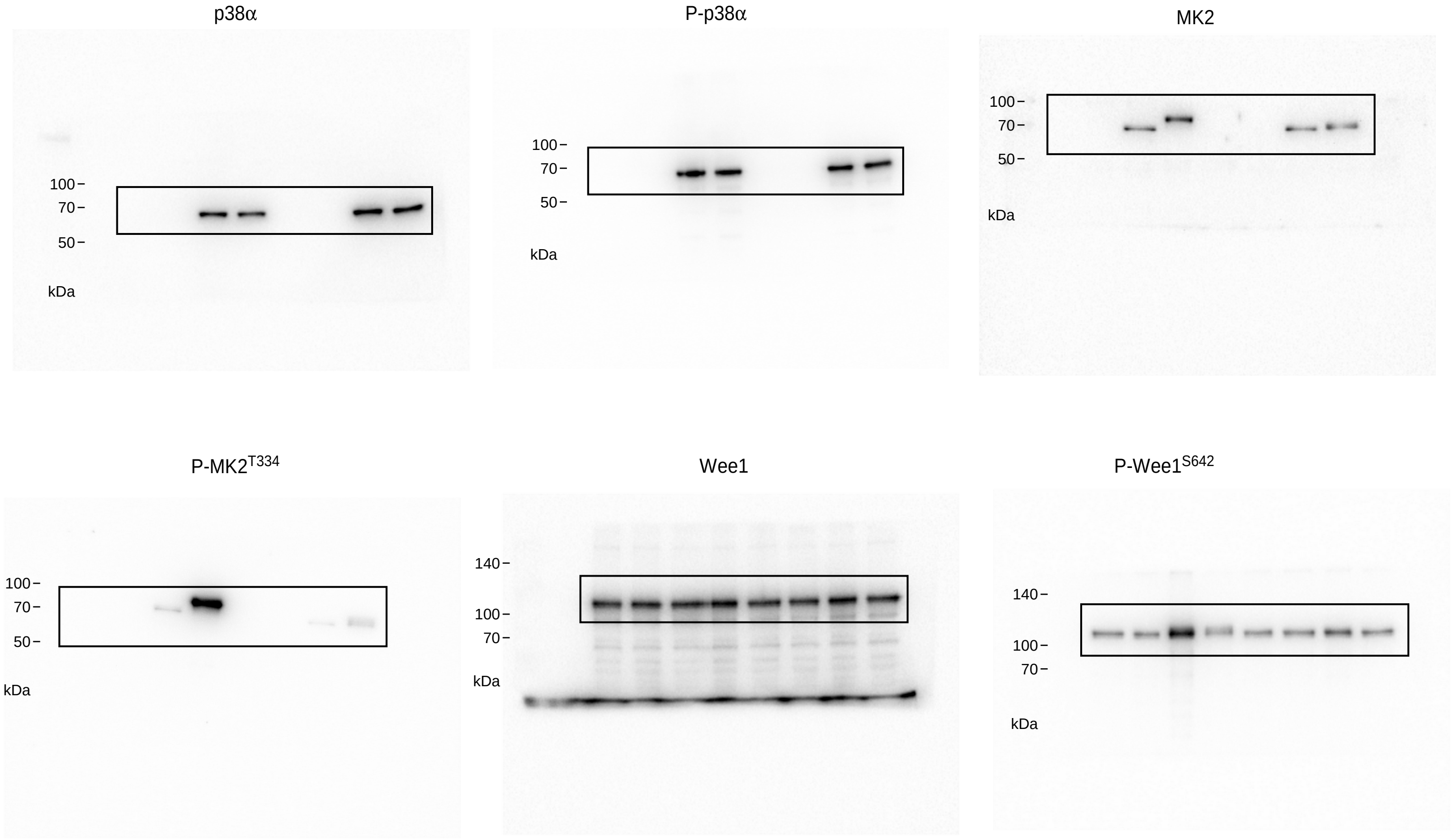

p38α
100
70
50
kDa
P-p38α
100
70
50
kDa
MK2
100
70
50
kDa
P-MK2T334
100
70
50
kDa
Wee1
140
100
70
kDa
P-Wee1S642
140
100
70
kDa

Figure 4c:

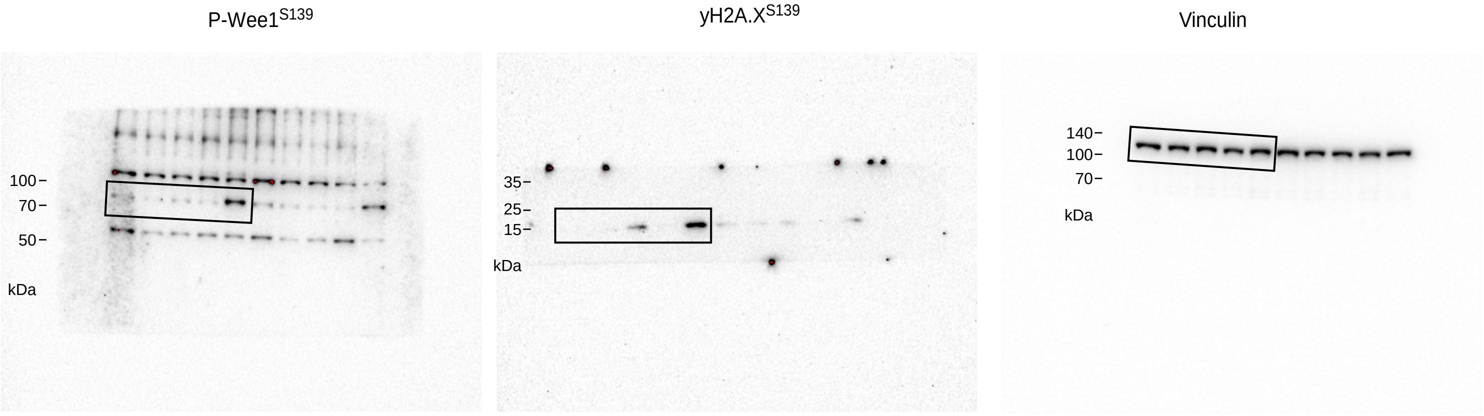
P-Wee1S139
100
70
50
kDa
yH2A.XS139
35
25
15
kDa
Vinculin
140
100
70
kDa

Figure 5e:

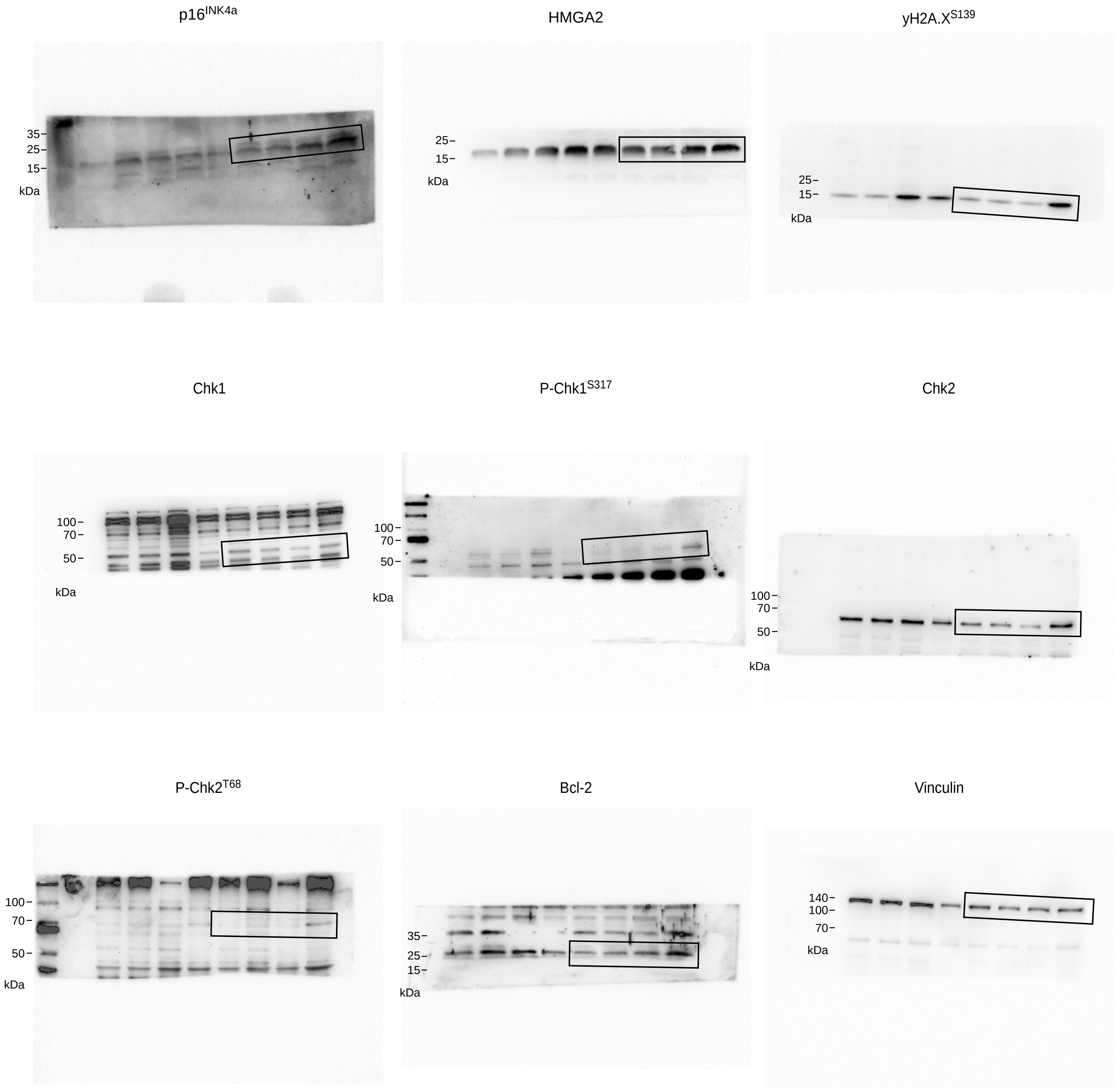
p16INK4a
35–
25–
15–
kDa
HMGA2
25–
15–
kDa
yH2A.XS139
25–
15–
kDa
Chk1
100–
70–
50–
kDa
P-Chk1S317
100–
70–
50–
kDa
Chk2
100–
70–
50–
kDa
P-Chk2T68
100–
70–
50–
kDa
Bcl-2
35–
25–
15–
kDa
Vinculin
140–
100–
70–
kDa

Extended Data Figure 1d,e:

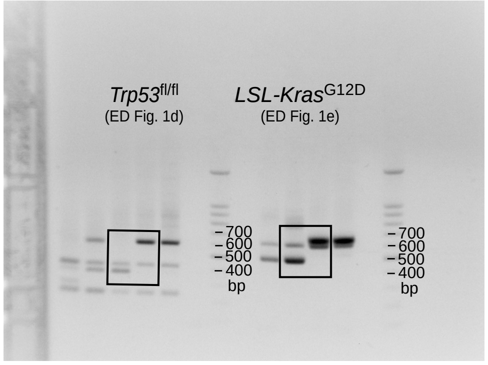

Extended Data Figure 1f:

APC

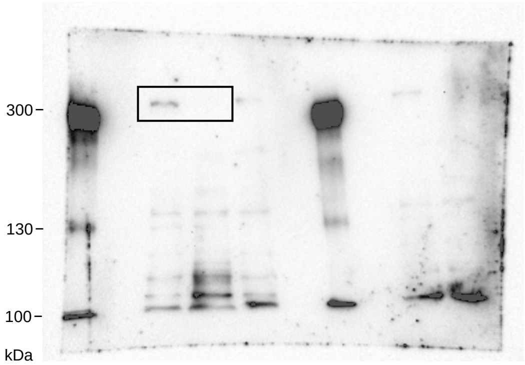

Vinculin

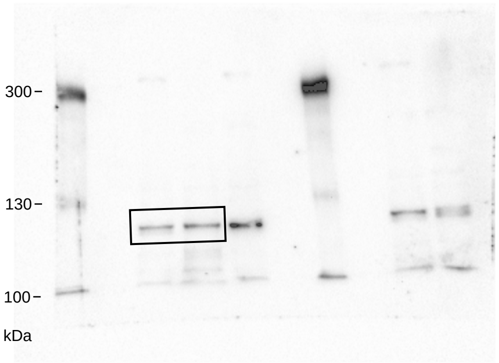

Extended Data Figure 1j:

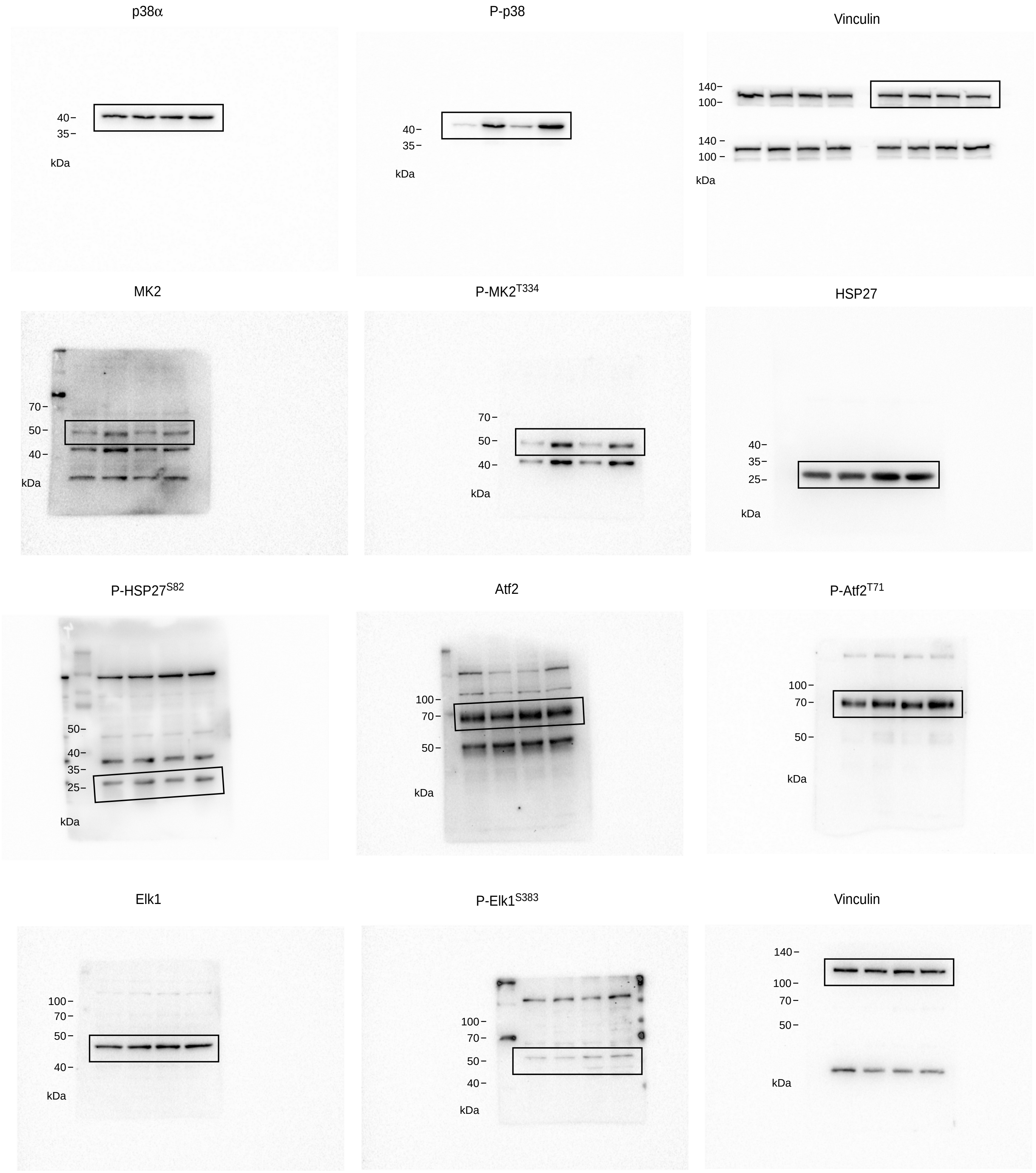

Extended Data Figure 1l:

p38α

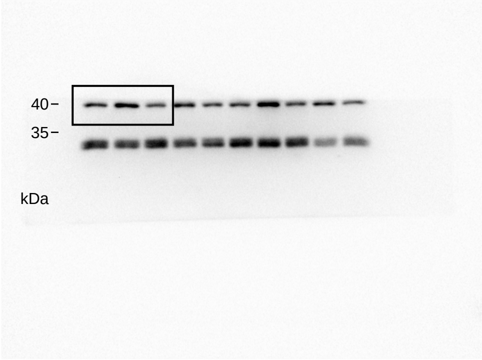

P-p38

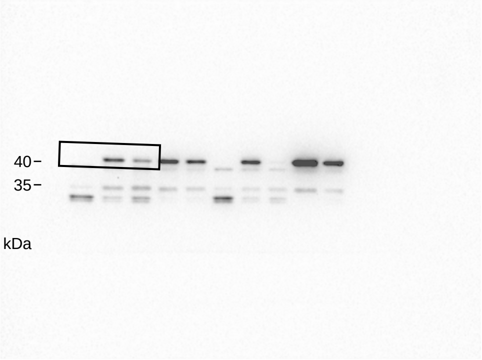

MK2

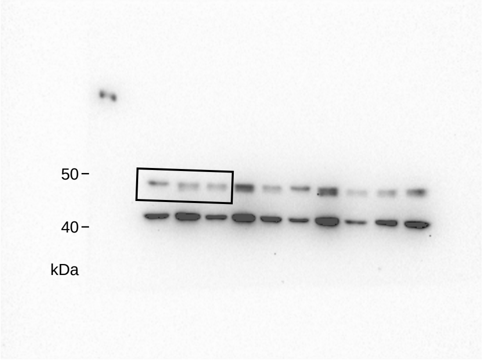

P-MK2^{T334}

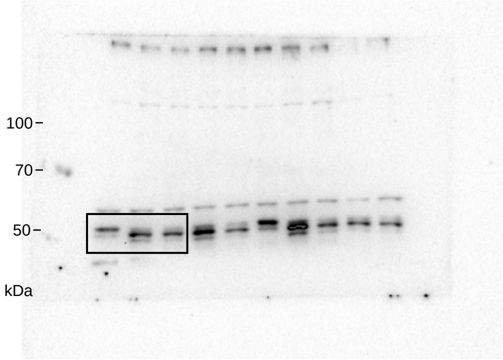

Vinculin

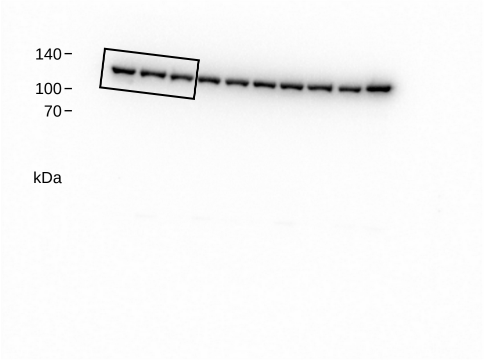

Extended Data Figure 1m:

p38α

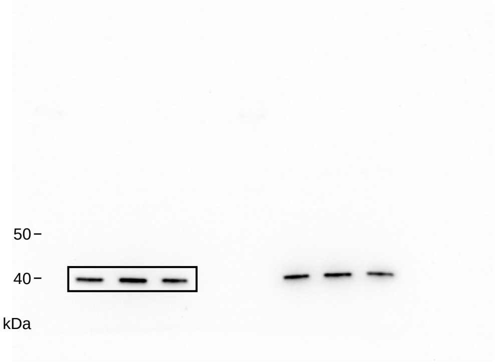

P-p38

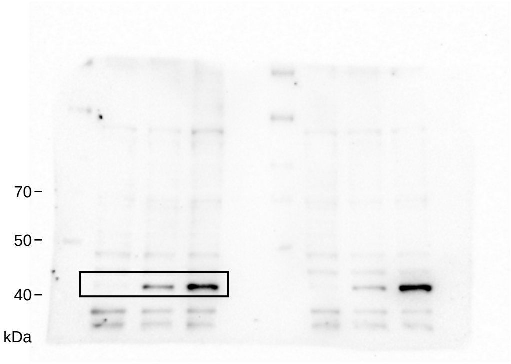

MK2

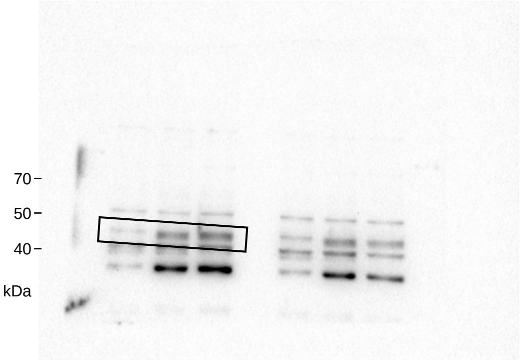

P-MK2^{T334}

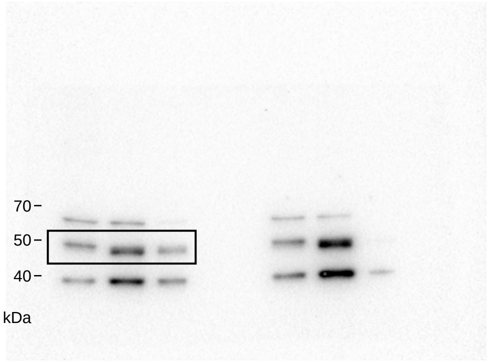

Vinculin

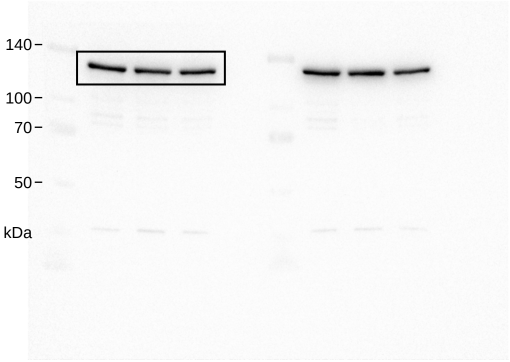

Extended Data Figure 3b:

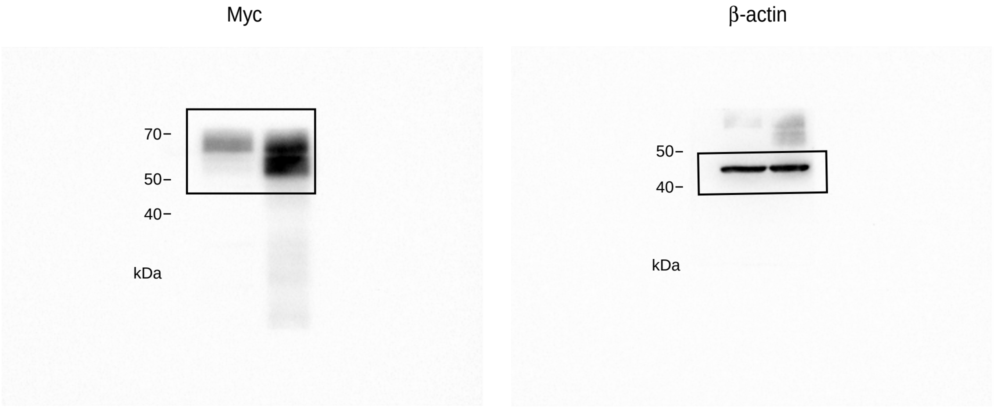

Extended Data Figure 3d:

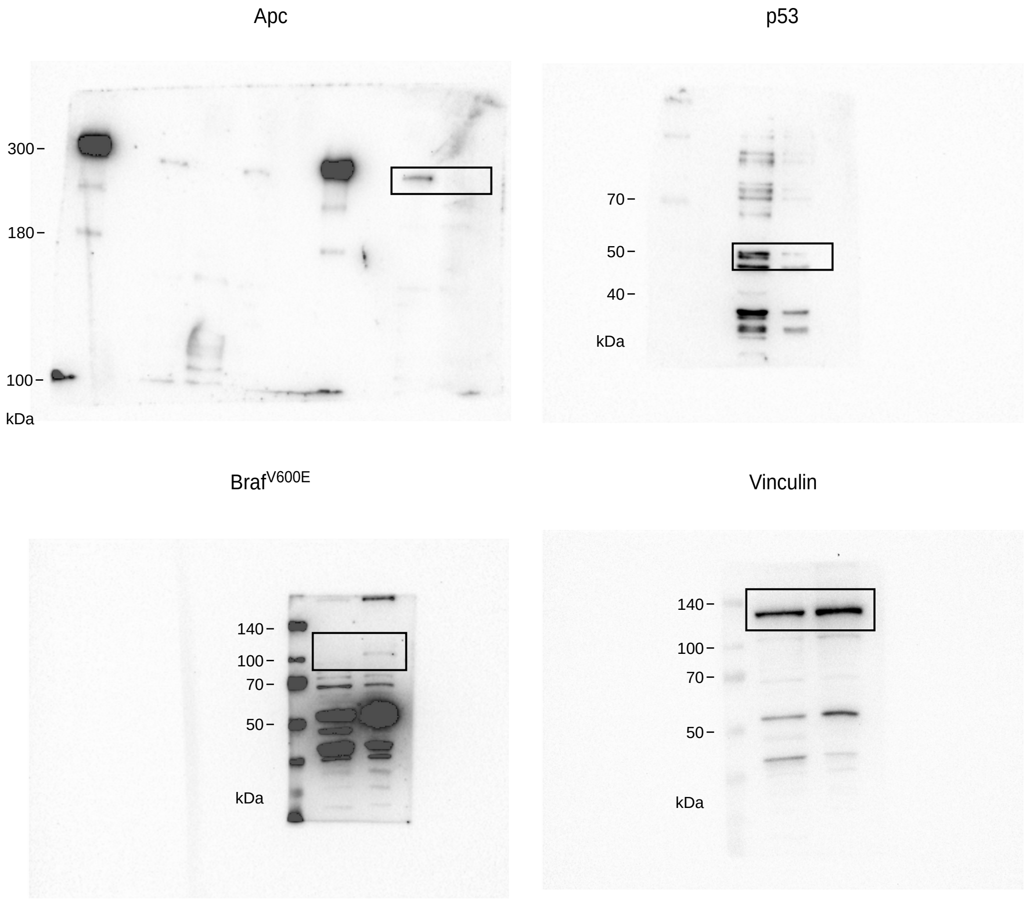

Supplement: Supplementary file 3 — Unprocessed western blots and gels. [file 43018_2024_899_MOESM3_ESM.pdf]
